# *EPDR1* up-regulation in human colorectal cancer is related to staging and favours cell proliferation and invasiveness

**DOI:** 10.1038/s41598-020-60476-7

**Published:** 2020-02-28

**Authors:** F. Gimeno-Valiente, Á. L. Riffo-Campos, G. Ayala, N. Tarazona, V. Gambardella, F. M. Rodríguez, M. Huerta, C. Martínez-Ciarpaglini, J. Montón-Bueno, S. Roselló, D. Roda, A. Cervantes, L. Franco, G. López-Rodas, J. Castillo

**Affiliations:** 1 Institute of Health Research, INCLIVA, Valencia, Spain; 2 Present Address: Departamento de Anatomía Patológica. Universidad de La Frontera, Temuco, Chile; 3 Department of Statistics and Operational Research, University of Valencia, Burjassot, Valencia, Spain; 4 Department of Medical Oncology, University Hospital, Universitat de València, Valencia, Spain; 5 Instituto De Ciencias Veterinarias Del Litoral (ICIVET Litoral), Universidad Nacional Del Litoral (UNL)/Consejo Nacional De Investigaciones Científicas Y Técnicas (CONICET), Santa Fe, Argentina; 6 Centro de Investigación Biomédica en Red en Cáncer (CIBERONC), Madrid, Spain; 7 Department of Biochemistry and Molecular Biology, Universitat de València, Valencia, Spain

**Keywords:** Transcription, Colorectal cancer

## Abstract

The finding of novel molecular markers for prediction or prognosis of invasiveness in colorectal cancer (CRC) constitutes an appealing challenge. Here we show the up-regulation of *EPDR1* in a prospective cohort of 101 CRC patients, in a cDNA array of 43 patients and in *in silico* analyses. *EPDR1* encodes a protein related to ependymins, a family of glycoproteins involved in intercellular contacts. A thorough statistical model allowed us to conclude that the gene is significantly up-regulated in tumour tissues when compared with normal mucosa. These results agree with those obtained by the analysis of three publicly available databases. *EPDR1* up-regulation correlates with the TNM staging parameters, especially T and M. Studies with CRC cell lines revealed that the methylation of a CpG island controls *EPDR1* expression. siRNA knocking-down and overexpression of the gene following transient plasmid transfection, showed that *EPDR1* favours cell proliferation, migration, invasiveness and adhesion to type I collagen fibres, suggesting a role in epithelial to mesenchymal transition. Both statistical and functional analysis correlated *EPDR1* overexpression with invasiveness and dissemination of tumour cells, supporting the inclusion of *EPDR1* in panels of genes used to improve molecular subtyping of CRC. Eventually, *EPDR1* may be an actionable target.

## Introduction

Colorectal cancer (CRC) is one of the most common malignancies worldwide^1^. In spite of the progresses of precision medicine, the long-term survival of patients with advanced metastatic disease is still poor^2^ and the burden of CRC is expected to increase by 60% in the next decade^1^. Nevertheless, this disease is a good candidate for screening programmes^3^ and its early detection improve treatment and survival. Most of CRC-related death is due to tumour metastasis^4^ and, therefore, finding of molecular markers for prediction or prognosis of invasiveness, which could be added to the available repertoire of potential markers^5–7^, constitutes an appealing challenge. This is particularly attractive in view of the growing interest in improving the clinical stratification of CRC^8,9^. This reason, together with the risks of metastatic dissemination, makes especially interesting the identification of potential actionable targets in CRC.

The *EPDR1* gene codes for a protein related to ependymins, a family of piscine brain glycoproteins^10^. Ependymins, encoded by *Epd* genes, are transmembrane proteins that play a role in intercellular contacts between neural cells^11^. It was early suggested that their extracellular domain may display antiadhesive properties^12^ and it shows a calcium-dependent ability to interact with collagen fibrils^13^.

The first report on the presence of ependymin-related proteins in mammals was published in 2001. Nimmrich *et al*., in a search for genes differentially expressed in tumour tissues, found a transcript in two CRC cell lines, which was absent from normal mucosa cells. The sequence of its cDNA displayed some similarity to the ependymin genes, and the gene, which was designed as *UCC1* (upregulated in colon cancer), was also found to be overexpressed in two out of the three analysed human cancer tissues when compared with paired normal mucosa^14^. Soon afterwards, Kirkland and his co-workers reported the presence of a gene related to ependymins highly expressed in hematopoietic cells, in some non-hematopoietic tissues, and in several malignant tissues and cell lines^15,16^. The gene, called *MERP1* (after mammalian ependymin-related protein), turned out to be the same as *UCC1*; both names are now used as aliases of *EPDR1* (*Epd*-related) gene.

Several other reports on the presence of *EPDR1* in humans have appeared in the literature. Alterations in the expression level or single-nucleotide polymorphisms in the *EPDR1* locus have been described in a variety of pathological or developmental processes, which involve in most, if not all the cases, a dysfunction of cell adhesion^17–26^. In 2016 we reported that *EPDR1* and its splicing isoforms are differentially expressed in human CRC cell lines^27^ and in this context, exploring the participation of *EPDR1* in the onset and/or development of CRC is particularly interesting.

Here we describe an analysis of *EPDR1* expression in a cohort of 101 CRC patients, as well as in a cDNA array of 43 patients. Our experimental results were checked with bioinformatic analyses of publicly available databases. When compared with normal mucosa, the gene is significantly up-regulated in tumour tissues and a significant relationship has been observed between *EPDR1* expression and TNM staging parameters of cancer, especially T and M stages. The mechanisms involved in these features were studied *in vitro* using several human CRC cell lines, to find that *EPDR1* increases cell proliferation, promotes cell migration, interaction with type I collagen fibrils and invasiveness.

## Results

According to the data retrieved from the Ensembl genome browser (www.ensembl.org), human *EPDR1* locus maps to chromosome 7 (37,683,843–37,951,936), and is transcribed from the forward strand. Alternative splicing gives rise to four isoforms, isoform 1 (201 according to Ensembl database) being the major one^27^. Isoform 2 (203), though minor, is interesting because it lacks the topogenic signal for the membrane location. A map of the locus is given in Supplementary Fig. S1.

### *EPDR1* is up-regulated in human CRC patients

**Figure 1:**
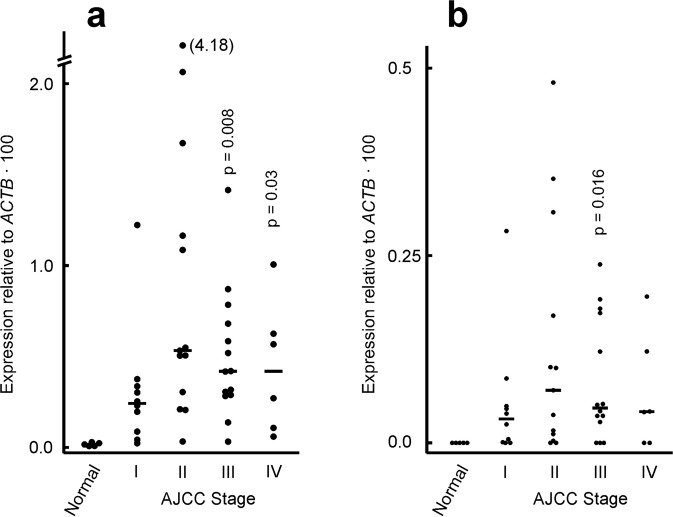
Expression of *EPDR1* in a cDNA array of normal colonic mucosa and tumours. The tumour samples were classified according to the AJCC stage. (**a**) Expression of total *EPDR1*. (**b**) Expression of *EPDR1* isoform 2. The p-values relative to normal samples, calculated by Student’s t-test, are given when a significant difference with normal mucosa is found. The position of median values is shown.

*EPDR1* expression was first analysed in a TissueScan cDNA array (OriGene) of human CRC patients (see Material and Methods). The level of whole *EPDR1* and of its isoform 2 was determined by RT-qPCR in all the samples. Expression of total *EPDR1* is detected in normal mucosa at a very low level, but is clearly expressed in almost all the tumour samples. Only in 5 out of the 43 tumour samples the fold change of *EPDR1* expression relative to the normal mucosa is less than 5. As shown in Fig. 1a, the up-regulation of the whole gene is found in all the CRC stages. The analysis of the gene expression using the usual ANOVA approach and multiple comparisons, revealed a significant difference (p = 0.041) between normal and tumour tissues. The behaviour of isoform 2 was somewhat different. First of all, as previously reported^27^, its expression level is low, and it is undetectable in normal tissues. Moreover, independently of the stage, the plots showed that in some CRC patients isoform 2 is not detected or expressed at a negligible level, while there are other patients showing a higher expression (Fig. 1b). Stage II patients show a large dispersion of expression values, both in whole *EPDR1* and in its isoform 2.

Then, a prospective cohort of 101 CRC patients who underwent surgery at the University Hospital (Valencia) in the last 3 years (Supplementary Table S1) was studied. The use of paraffin-embedded samples did not allow us determining the level of the minor isoform 2 and only the expression of the whole gene is reported here.

**Figure 2:**
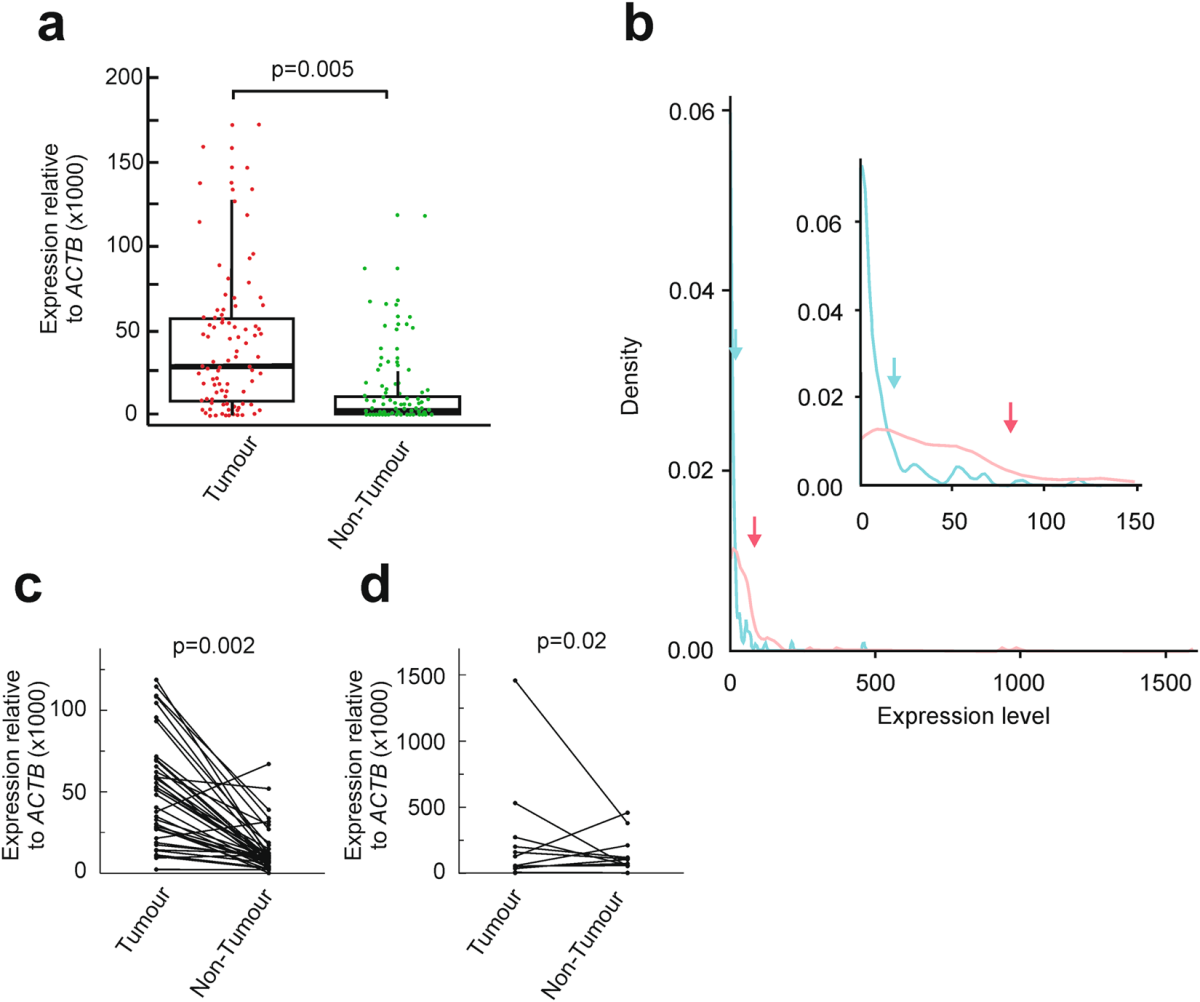
Expression of total *EPDR1* in a cohort of 101 CRC patients from our University Hospital. (**a**) Box plots with whiskers with maximum 1.5 IQR of total *EPDR1* expression measured by qPCR in tumour and non-tumour adjacent tissues. Outliers with expression values higher than 200 (4 from tumours and 3 from non tumour samples) are not included in the graph, but they are shown in panel **d**. Statistical analysis was carried out with Student’s t-test. (**b**) Density plot of the expression values for tumour (red) and non-tumour adjacent tissues (blue). The arrows point to the position of the means. The inset shows a magnification of the plot to appreciate the differences between the two tissue types for expression values lower than 150. (**c**) Up-regulation of *EPDR1* in localized tumour samples when compared with paired normal adjacent tissue. (**d**) As **c**, but with samples from metastatic patients. Statistical analysis was carried out with tailed Student’s t-test.

We first determined the means of *EPDR1* mRNA expression, measured by RT-qPCR, in 97 paired tumour and normal samples from the 101 patients’ cohort. The remaining 4 samples were not apt for processing. A paired t-test showed that the average expression in tumours is significantly higher (p = 0.005) than in normal tissues (Fig. 2a). This may be also observed in the density plot built with the experimental results obtained from the 97 paired samples (Fig. 2b). Most of the expression values of *EPDR1* in normal adjacent tissues group as a single skewed peak, with some outliers at high values. However, the expression of the gene in tumour samples results in a wider distribution. The arrows mark the positions of the means, clearly larger than the medians given in the box plot of Fig. 2a. This is related to the skewness to the right observed in the density plot. The plots for single paired samples obviously show that, in most cases, the expression of the gene is higher in tumour tissues than in adjacent mucosa in both non-metastatic and metastatic patients (Fig. 2c,d). Moreover, the up-regulation of the gene is observed irrespective of the AJCC stage of the patients. In the case of stage I patients the difference is close to significant, while the significance is clear for the remaining stages (Table 1). As a further control, we determined the expression of the gene in the only four benign colon polyps available from patients. The resulting value (0.013 ± 0.006 relative to β-actin gene) was not included in Table 1 because of the small sample size, but it falls in the range of non-tumour samples given in that Table.

**Table 1:** Expression of *EPDR1* gene in the local patients’ cohort (n = 97).

	1st Qu	Median	Mean	3rd Qu	95% CI of differences		p value
					Lower	Upper	
Total non-tumour	0.11	2.79	17.97	12.39			
Total tumour	9.34	32.84	81.43	62.11	16.67	7.79	0.005**
Stage I	15.05	27.45	54.33	63.83	−0.36	73.08	0.052
Stage II	14.26	28.79	35.93	51.33	0.11	35.93	0.049*
Stage III	8.53	32.84	40.81	62.11	6.35	39.33	0.007**
Stage IV	6.85	54.94	262.16	158.98	7.79	480.57	0.044*

mRNA expression values are given relative to the expression of ACTB gene (x1,000).

Statistical analysis was carried out as described in the Supplementary information.

Next, our statistical analysis focused on the study of the differential expression of *EPDR1* with respect to important predictors, using the data derived from our local patients’ cohort. It is usual to include marginal descriptive studies of the expression of a gene in tumour and adjacent tissues with respect to these predictors and these are included in the Supplementary Material. Nevertheless, a thorough statistical analysis is described here. Both the expression differences between tumour and adjacent tissues and the expression ratio tumour/adjacent tissue were calculated for all the 97 paired samples and their dependence with respect to the patients’ characteristics and other predictors (T, N, M and AJCC stages) was determined by using multiple regression. A stepwise variable selection has been performed as described under Materials and Methods.

When the analysis included the TNM and the AJCC stages, a significant coefficient in the ratio tumour/adjacent tissue expression was observed with T3 and T4 stages (p = 0.0021 and p = 0.0009, respectively), with M1 stage (p = 0.0004) and with AJCC stage II (p = 0.0005). No significant coefficients were observed between the ratios and N stages or with AJCC stage III. These results are summarized in Table 2. The p-value of the test for null coefficients corresponding to the variables T2, T3 and T4 is 0.00038. When the categorical predictors corresponding to N and M stages were removed from the model, a significant correlation with AJCC stages III and IV (p = 0.0013 and p = 4.8·10^−7^) was also observed (see Supplementary Material). All these results manifest that a strong relation exist between *EPDR1* expression in CRC tissues and the invasiveness of tumour cells.

**Table 2:** Summary of the results obtained in the statistical analysis of the *EPDR1* expression in the local patients’ cohort (n = 97).

Parameters		Fold-change (Means)	Model p-value^a^	Global test p-value^b^	Expression difference (Means)	Model p-value^a^	Global test p-value^b^
T	T1	32.003			36.457		
	T2	247.121	0.9969 ns	0.0003***	171.332	0.9292 ns	
	T3	65.057	0.0021**		45.833	0.0014**	8.0·10^−5^***
	T4	41.005	0.0009***		26.697	0.0005***	
N	N0	59.194			44.437		
	N1	135.319	0.1162 ns	0.2258 ns	95.265	0.0148*	0.0356*
	N2	117.561	0.0869 ns		80.234	0.0113*	
M	M0	31.200			33.084		
	M1	358.940	0.0004***	0.0004***	206.376	0.0066**	0.0066**
AJCC	I	31.707			40.776		
	II	28.262	0.0005***	0.0020**	28.839	0.0003***	
	III	33.087	0.2062 ns		31.172	0.5734 ns	0.0007***
	IV	358.940	NA		206.376	NA	

^a^This column gives the p-value of each item relative to the reference values (T1, N0, M0 and stage I).

^b^These p-values correspond to the global test for the respective set of parameter values.

ns: non-significant. NA: not available in the model used.

Statistical analyses were carried out as described in the Supplementary information.

When the analysis was done with the differences of expression between tumour and adjacent mucosa as response rather than with the ratios tumour/adjacent tissue, roughly similar results were obtained, but significant coefficients were also observed with the N stages (Table 2). The reasons for using this double system of comparing the expression in tumour and adjacent tissues will be commented under the Discussion.

Next we asked if there exist a dependence of the patients’ survival with respect to the level of gene expression. To answer this question, we have used a Cox regression analysis (see Supplementary Material, Statistical Analysis). The dependence of the survival time on *EPDR1* expression in tumours is close to significance (p = 0.085), but a very significant dependence was observed with respect to the expression of the gene in the histologically normal tumour borders (p = 0.0057). When a Cox regression including the AJCC stage was carried out, the survival time was also found significantly dependent on the *EPDR1* expression in tissues adjacent to the tumour (p = 0.0344) but not on the expression in the tumour itself (p = 0.1161). Similar results were obtained if the Cox regression included the T stage. In this case significant dependence on the expression in non-tumour adjacent tissue was found (p = 0.0127) but no significant dependence on the expression in tumour was found (p = 0.2188). In other words, the expression of *EPDR1* in the environment of the tumour tissues seems to be a better predictor of survival time than the expression in the tumour itself.

The results obtained with the local cohort of CRC patients were compared with those obtained *in silico* by using TCGA data. This database includes expression values for *EPDR1* in a cohort of 622 CRC patients. When a Welch two sample t-test was used to compare the expression means in both groups of samples, tumour and normal tissue, it was found that the expression in tumour is 2.08 fold higher than in normal tissues (p = 9.67·10^−9^). The comparison of the paired samples included in the TCGA cohort (n = 50) also allowed us to conclude that *EPDR1* expression is significantly higher in tumour than in paired adjacent mucosa (Supplementary Table S2). Finally, Kim *et al*.^28^ generated a series of RNA-seq data from 18 CRC metastatic patients in which they included samples from normal mucosa, primary tumours and metastasis. The database is publicly available (https://www.ncbi.nlm.nih.gov/bioproject/218851) and we used it to recover the *EPDR1* expression values. The gene expression in primary tumours is 2.15-fold increased over that in normal mucosa and, interestingly, the fold change increase is 2.518 when the expression in metastases is compared with that of normal mucosa (Supplementary Table S3). All the results acquired *in silico* both from TCGA and from the Kim *et al*.^28^ data agree with those obtained from our local patients’ cohort. A complete description of the model used for statistical analysis of the data sets is given in Supplementary Material.

In summary, the analysis of expression data in a large number of patients, both *in vivo* and *in silico*, allowed us to conclude that *EPDR1* is significantly up-regulated in tumour tissues when compared to adjacent mucosa. Moreover, the statistical analysis indicated that expression in tumour correlated with invasiveness and dissemination of tumour cells. Therefore, we next addressed the questions of the possible causes leading to a differential expression of the gene and of the mechanisms that might relate *EPDR1* up-regulation to CRC progression. To answer some of these questions we used *in vitro* models with human CRC cell lines, as described below.

### *EPDR1* expression is regulated by DNA methylation in CRC cell lines

**Figure 3:**
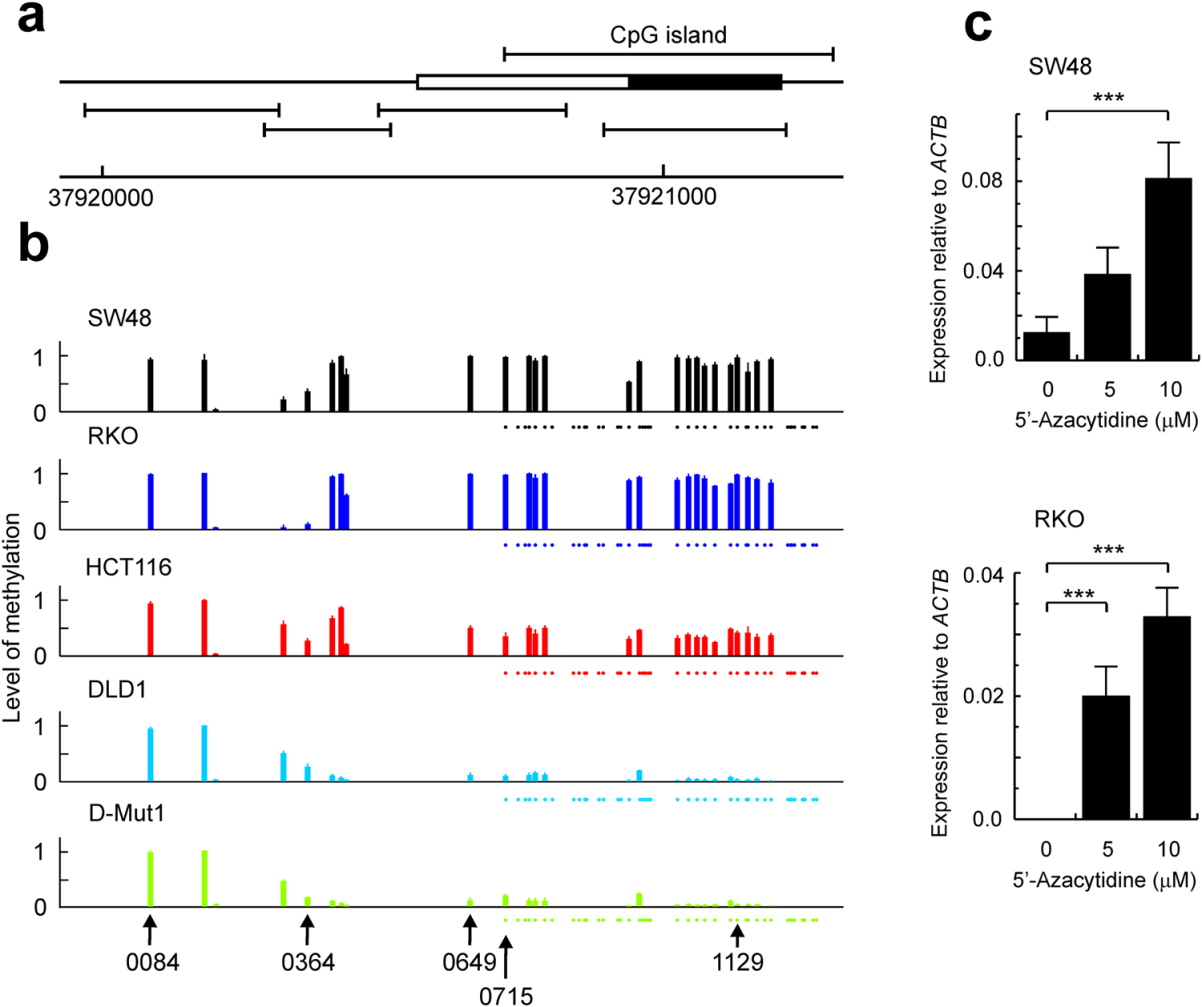
Epigenetic regulation of *EPDR1* expression in CRC cell lines. Quantitative DNA methylation analysis was carried out by a method using an AGENA’s MassARRAY platform as described in the text. (**a**) Map of the promoter and first transcribed exon in the major isoform 1 (see Fig. S1 for a map of the entire locus). The exon is depicted as a box, in which the filled region stands for its translatable region. The location of the CpG island encompassing this exon is shown. The bars below the map mark the location of the amplicons used for the methylation analysis. The scale gives the absolute position of the region, in bp, in chromosome 7. (**b**) Level of methylation of the analysed CpGs in different CRC cell lines. The dots below the graphs mark the position of the different CpGs within the island, even when their methylation level could not be determined with the method used. The results represent the average of 5–10 determinations with their SD. Some CpGs, identified at the bottom with their location in chromosome 7 (for simplicity, only the last four digits are given), have been analysed for their methylation status in CRC patients^29^ as commented in the main text. (**c**) Effects of 5′-azacytidine treatment on the expression of *EPDR1* in SW48 and RKO cell lines; in both cases, the demethylating agent causes an increase in gene expression. The Kruskal-Wallis statistical test was used to analyse the significance of the results. ***p < 0.001.

We have previously found that the expression of the whole *EPDR1* gene is not detected in RKO, almost negligible in SW48 (0.00012 ± 0.00007 relative to *ACTB*), low in HCT116 (0.0054 ± 0.0004), and high in DLD1 (0.0252 ± 0.0056) and D-Mut1 (0.0454 ± 0.0055)^27^. We hypothesize that epigenetic changes may cause these differences in *EPDR1* expression among the cell lines used. To check that hypothesis, the methylation of a segment of 1241 bp, which includes a CpG island encompassing *EPDR1* exon 3, the first transcribed exon except for isoform 3, and the first translatable exon in the major isoform, was determined. A schematic map of the analysed region, including the location of amplicons used, is depicted in Fig. 3a. Interestingly, the methylation level of the analysed CpGs is compatible with the idea that methylation of the CpG island is the cause of *EPDR1* repression in SW48 and RKO cells and of the low expression in HCT116 (Fig. 3b). The methylation of the distal CpGs upstream of the island does not vary from line to line and this serves as an internal control of the results. In a comparison of the methylomes of normal colon mucosa, tubular adenomas, and colorectal cancers. Luo *et al*. described the methylation status of 5 of the CpGs we have analysed^29^ and they are identified at the bottom of Fig. 3b. Interestingly, these authors showed that the CpG at location 37,920,084 is hypermethylated irrespective of the nature of the sample, and we found that this CpG is hypermethylated regardless of whether the gene is expressed or not in the cell lines tested. Therefore, the results presented here agree with the constitutive hypermethylation found in that CpG in human colonic tissues.

To further check that the CpG island methylation is related to *EPDR1* transcription, the expression of the gene was determined in RKO and SW48 cells grown in the presence of 5-azacitidine. A significant, dose-dependent increase in transcription of the gene was observed in both cell lines (Fig. 3c), showing that demethylation of DNA actually causes the activation of *EPDR1* expression. Anyway, the fact that the island is virtually non-methylated in both DLD1 and D-Mut1cells, which show high, but distinct, levels of *EPDR1* transcription, suggests that some additional mechanism must exist to account for that difference.

### *EPDR1* increases cell proliferation in CRC cell lines

**Figure 4:**
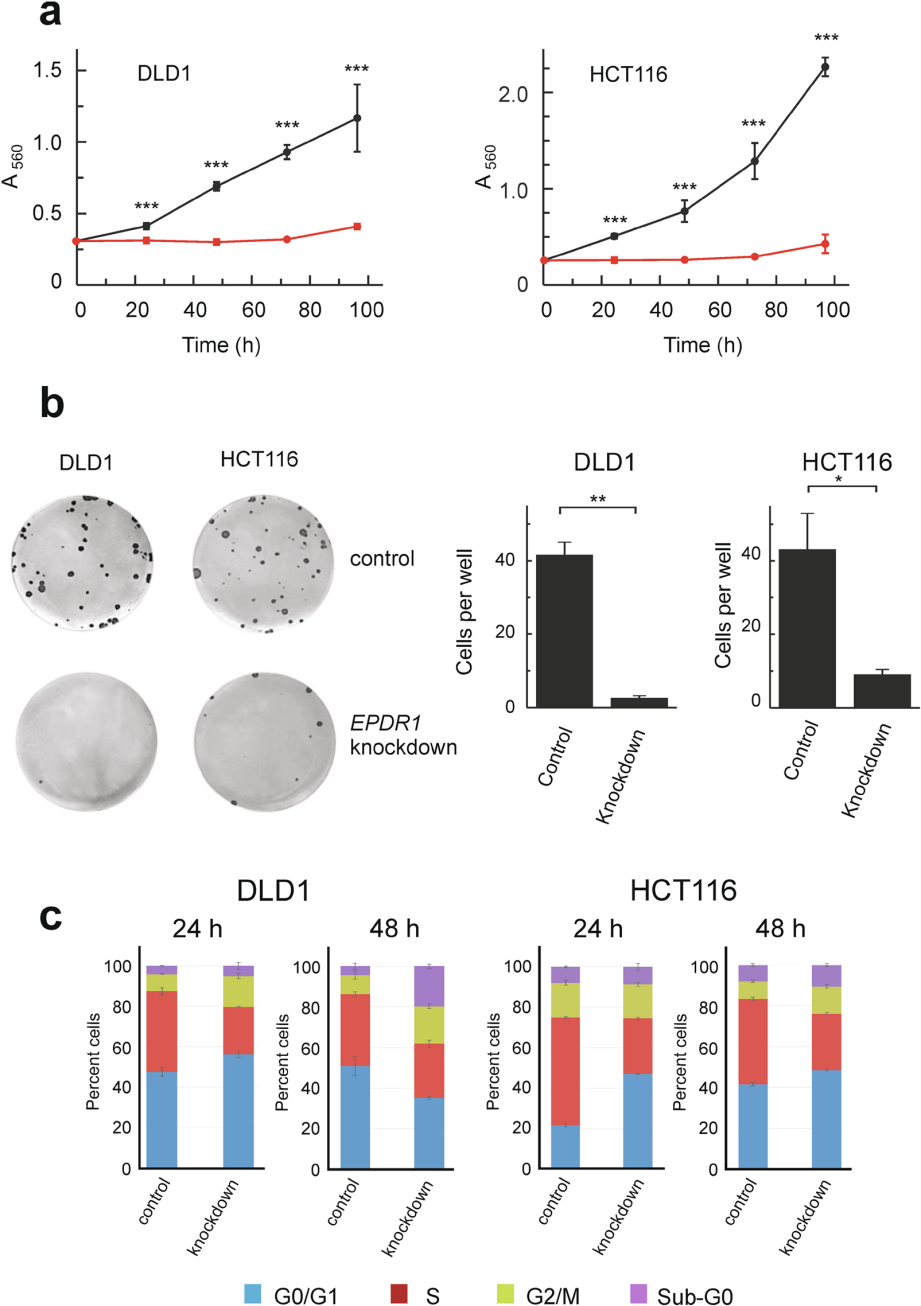
Effects of *EPDR1* knocking-down with si1 on the growing of CRC cell lines. (**a**) MTT assay of cell proliferation in two *EPDR1*-expressing cell lines. The graph shows the results of two independent experiments, each one with three independent transfections, as described under Materials and Methods. After *EPDR1* silencing (to the level shown in Fig. S2) the proliferation of cells (red lines) is almost negligible in comparison with that of the cells treated with scrambled siRNA (black lines). Student’s t-test was used to analyse the significance of the differences between silenced and control cells at every time point. (**b**) Colony formation assays of cells treated with scrambled siRNA (control) and with si1 (*EPDR1* knockdown) were carried out in 6-well plates as described under Materials and Methods. Two independent experiments (each one with three independent transfections) were done with every cell line. Both the photograph of representative plates and the averaged quantification of the number of colonies in each of the three wells are given. (**c**) Flow-cytometry cell cycle analysis (ModFit TL software) of cells treated with scrambled siRNA (control) and with si1 (*EPDR1* knockdown). The procedure was done with three independent transfection wells in two independent experiments with either siRNA and in both cell lines. Statistical analysis was done by Student’s t-test. *p < 0.05; **p < 0.01; ***p < 0.001.

To explore the function of *EPDR1* in human CRC progression, its effects on cell growth were first analysed. To do this, the gene was independently silenced with two *EPDR1* siRNAs, further referred to as si1 and si2, as mentioned under Material and Methods. Both siRNAs efficiently knocked down the expression of *EPDR1* both at the level of mRNA and protein (Supplementary Fig. S2). The consequences of this knockdown were evaluated by MTT, colony formation assays and flow cytometry (Fig. 4 and supplementary Fig S3). The assays show that *EPDR1* significantly favours proliferation of DLD1 and HCT116 cells. Silencing the gene with siRNAs affects the progression of the cell cycle in both DLD1 and HCT116 cells (Fig. 4c).

When the results obtained with si1 (Fig. 4) and si2 (supplemental Fig. S3) are compared some differences were observed, which may suggest that the cytotoxicity of both siRNAs is different in each cell line. For instance, the sub-G0 population at 48 h is higher in si1-treated DLD1 than in HCT116 cells, while an opposite result was obtained with si2. A reliable characterization of the sub-G0 population cannot be achieved by a simple cell cycle analysis. To overcome this difficulty, an analysis in the presence of 7-aminoactinomycin D and Annexin V was carried out to discriminate between apoptotic and necrotized cells. Supplementary Fig. S4 shows the results of an experiment with DLD1 in which untreated cells and cells transfected with scrambled siRNA or with si1 or si2 were analysed after 72 h of transfection. It is clear that the differences in cytotoxicity found between si1 and si2-silenced cells are mainly attributable to a greater necrosis induced by si1 in this cell line. The use of untreated DLD1 cells in the experiment of supplemental Fig. S4 shows that neither si2 nor control siRNA affect the viability of the cells in a significant way. Moreover, both si2 and scrambled siRNA induce apoptosis and necrosis to an extent similar to that of untreated cells. To further explore the differences in cytotoxicity between si1 and si2, we transfected RKO cells, in which *EPDR1* is not expressed^27^, with si1 and si2. No consequences in proliferation or cell migration were observed (Supplementary Fig. S5) and the sub-G0 population was similar between cells transfected with si1 and si2 (12.39 ± 1.66% and 13.29 ± 1.96% respectively). These results allowed us to discard the possibility that the phenotypic effects of si1 and si2 were due to off-target effects.

### *EPDR1* promotes cell migration, invasiveness and adhesion to collagen type I fibres

**Figure 5:**
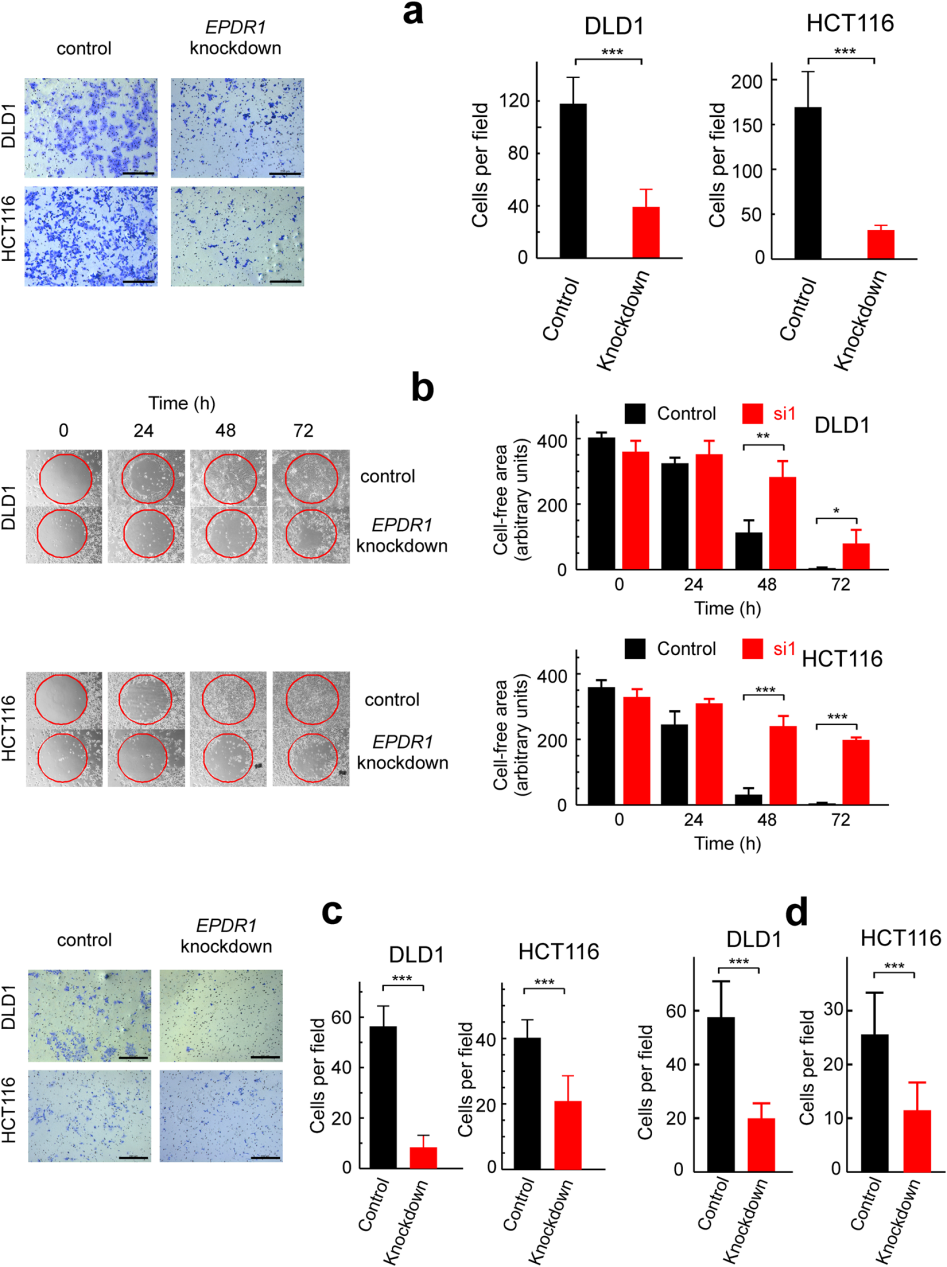
Effects of *EPDR1* knocking-down with si1 on the migration invasiveness and adhesion of CRC cell lines. (**a**) Transwell migration assay of cells treated with scrambled siRNA (control) and with si1 (*EPDR1* knockdown). A photograph of representative plates is shown (left). Cells were counted under microscope in 6 different areas of every plate and the counting values were averaged (right). (**b**) Wound-healing assay of cells treated with scrambled siRNA (control) and with si1 (*EPDR1* knockdown). A photograph of representative plates is shown at the left. The times shown were measured after removing the central gel layer (red circle). The experiment was carried out in triplicate and the average of three determinations of the cell-free areas, as measured by ImageJ is shown at the right. (**c**) Assay carried out as in **a**, but through a Matrigel layer. (**d**) Effects of knocking-down the gene *EPDR1* on the adhesion of cells to type I collagen-coated plates. The total number of cells were counted as in **a**. The size bars in **a** and **c** correspond to 200 µm. Statistical analyses were carried out by Student’s t-test. ***p < 0.001.

The effects of silencing *EPDR1* on cell migration were next studied in DLD1 and HCT116 cell lines. The results of the transwell analysis (Fig. 5a) showed that *EPDR1* knockdown significantly reduces cell migration, and the wound healing assay (Fig. 5b) confirmed these results. When the transwell analysis was carried out through a Matrigel layer, the results indicated that *EPDR1* is also involved in migration through extracellular matrix (Fig. 5c). It can be, therefore, concluded that *EPDR1* promotes cell migration and invasiveness.

As migration of cells is facilitated by binding of cells to collagen type I fibres^30,31^, the effects of silencing *EPDR1* on cell adhesion to collagen-coated plates was next examined. The results show that knocking-down the gene significantly reduces the adhesion of DLD1 and HCT116 cells to collagen type I fibres (Fig. 5d). When si2 was used to knock-down *EPDR1* similar results in cell migration, invasiveness and adhesion to collagen assays were obtained (Supplementary Figure S6).

**Figure 6:**
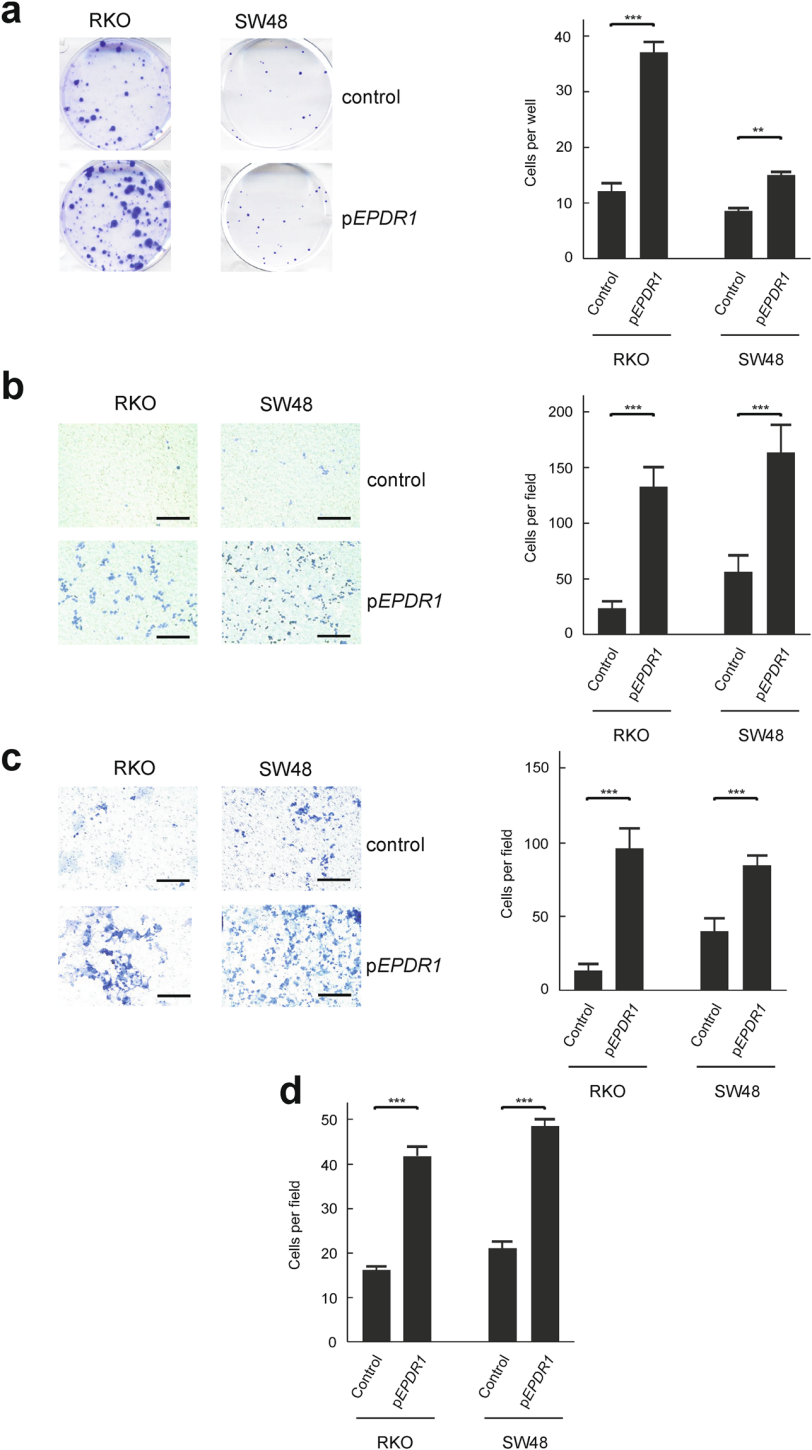
Effects of *EPDR1* overexpression on the phenotype of CRC cells. (**a**) Colony formation assays of cells transfected with control empty plasmid or with p*EPDR1* were carried out in 6-well plates as described under Materials and Methods. Two independent experiments (each one with three independent transfections) were done with every cell line as described under Materials and Methods. Both the photograph of representative plates and the averaged number of colonies in each of the three wells are given. (**b**) Transwell migration assay of cells transfected with control empty plasmid or with p*EPDR1*. Photographs of representative plates are shown and cell counting results are given. Cells were counted under microscope in 6 different areas of every plate and the counting values were averaged. (**c**) Assays carried out as in (**a**), but through a Matrigel layer. Photographs of representative plates are shown and cell counting results are given. Cells were counted and averaged as in (**b**). (**d**) Cell counting after adhesion of transfected cells to type I collagen-coated plates. Results were averaged as in (**b**). The size bars in (**b**,**c**) correspond to 200 µm. Statistical analyses were carried out with the Student’s t-test relative to control. **p < 0.01; ***p < 0.001.

Once studied the effects of knocking-down *EPDR1*, it can be wondered whether the opposite effects are observed by overexpressing the gene. To answer this question, we used the RKO and SW48 cells, which, as mentioned above, are characterised by a null or negligible expression of *EPDR1*. Supplementary Figure S7 shows that transfecting the cells with p*EPDR1* resulted in a dramatic increase of gene expression. In both, RKO and SW48, overexpression of *EPDR1* resulted in a significant increase in colony formation, migration, invasiveness and adhesion to type I collagen of the cells (Fig. 6). These results confirm the proposed role for *EPDR1* in increasing the dissemination of cancer cells.

## Discussion

Molecular subtyping based on gene expression is gaining interest in cancer stratification^32^ and proposals for CRC have been done^8^. It has been recently emphasized that transcriptional signatures may allow a refinement of the CRC subtyping^9^ and, in this sense, finding novel genes dysregulated in the disease, either in tumour itself or in its environment, may aid to construct gene panels useful for diagnosis, prognosis or targeted therapy selection.

In the present paper, we show that *EPDR1* gene is up-regulated in CRC. The first report in this sense was published long time ago but it was limited to three patients^14^. When all the experimental data described in the present paper had been obtained, Chu *et al*. reported that *EPDR1* is down-regulated in CRC tumour cells when compared with adjacent mucosa. They analysed a local cohort of 75 patients, but the mRNA expression of *EPDR1* mRNA was only determined in a sub-group of 23 paired samples^33^. The cohort analysed in the present work is large enough and the thorough statistical analysis carried out allows us to draw more significant conclusions. Moreover, the results presented here include as well an additional analysis of 43 patient samples from a cDNA array, in which we have also shown the overexpression of isoform 2, and all of our data agree with the huge number of values recovered *in silico* from publicly available databases. Nevertheless, the above comments do not imply that every single tumour express higher levels of *EPDR1* than normal mucosa cells. Figure 2a shows that the expression level of the gene in some individual tumours is as low as in normal adjacent tissues or in benign colon polyps. A similar comment can be done regarding the *EPDR1* expression in cell lines. In a previous paper we reported the analysis of *EPDR1* in five CRC cell lines and some of their isogenic derivatives, to show that the expression of the gene is negligible in some of them^27^, as it occurs with the colorectal normal NCM460 cells^14^.

Apart from the bioinformatic analysis of TCGA data reported above, the expression of *EPDR1* in other tumours was recovered from that database by using the facility Gene Expression Profile Interactive Analysis (http://gepia.cancer-pku.cn/). In some other cancers, such as hepatocellular carcinoma, kidney carcinoma, cholangiocarcinoma, pancreatic adenocarcinoma and stomach adenocarcinoma, the gene expression profile is also elevated when compared with paired normal tissues. Nevertheless, uterine carcinosarcoma, bladder urothelial carcinoma and lung squamous cell carcinoma, among others, behave in an opposite way.

The results of gene expression in tumour and adjacent normal tissues are usually given as the fold change, *i.e*., the ratio of expression in tumour and adjacent tissue. Though useful for comparative purposes, some flaws may exist in this procedure, especially when the expression in normal tissues is very low or even below the detection limit. In these cases, the absolute error of the determination may result in large errors in the ratio, abnormally high values in it, or even a senseless infinite value. To prevent these errors, in the present paper, apart from the customary fold change values, we have also statistically analysed the results subtracting the expression in normal tissues from that in tumour samples. Both procedures, fully presented in the Supplementary Material, roughly gave similar results, except in some instances, mentioned under Results.

Of note, high-level expression of *EPDR1* is associated with T and M parameters and, less significantly, with N. This is clearly in accordance with the results obtained after knocking-down or overexpressing the gene in CRC cell lines. As mentioned above, the *in silico* analysis of the data obtained by Kim *et al*.^28^ revealed that *EPDR1* expression in metastatic tumours is higher than that in the primary CRC tissues. These results are in accordance with the high level expression of the gene in metastatic tumours described here and with the proposed role for *EPDR1* in EMT.

In strong disagreement with the data of Chu *et al*.^33^ our results on the effects of silencing *EPDR1* clearly show that the gene favours not only cell proliferation, but also invasiveness (Fig. 4a and Supplementary Fig. 3a). These facts may be the cause of the involvement of *EPDR1* with tumour development *in vivo*. Moreover, our results strongly suggest that the ectodomain of EPDR1 possesses antiadhesive properties as the related ependymins do^12^. The dissemination of cancer cells may be facilitated by the effects of EPDR1 on adhesion to type I collagen fibres, which have been described as the “highways” for tumour cell migration^31^. The interaction of fish ependymins with collagen is calcium-dependent^13^; should this dependence also take place in EPDR1, an interesting consequence might result. *STIM1* has been found to be overexpressed in CRC and promotes cell motility and EMT^34,35^. The gene encodes an endoplasmic reticulum sensor, which regulates the concentration of Ca^2+^, in cancer cells^36^. Therefore, it is tempting to speculate that *EPDR1* might be a downstream target of *STIM1*, which would ultimately cause, in a Ca^2+^-dependent manner, the cell migration and EMT by affecting cell adhesion and/or interaction with type I collagen fibres. Experiments to check this hypothesis are currently being done in our laboratory.

We have also shown that *EPDR1* is regulated by DNA methylation at an island encompassing the first translatable exon in the major isoform, although some other regulatory mechanism must exist. Methylation-dependent regulation of *EPDR1*, using a region shorter than ours, but overlapping with it, has also been reported in the recent paper of Chu *et al*.^33^. Luo *et al*., in the previously mentioned methylome analysis, found that in most stage IV patients the methylation of CpGs located at positions 37,920,649 37,920,715 and 37,921,129 is very low, although there is a subgroup of patients in which these CpGs are hypermethylated^29^. Present results show that the highest expression values of *EPDR1* are found in some stage IV patients (Fig. 2d) and, therefore, our findings are in agreement with an important, though not unique, role of DNA methylation in the regulation of *EPDR1* expression.

It is worth noting that patients’ survival depends more on the expression of *EPDR1* in non-tumour adjacent mucosa than in tumour itself. The adjacent non-tumour tissues are defined as normal from a histological point of view, but they may be abnormal when their molecular characteristics are taken into account. For instance, it has been shown that histologically normal tissues surrounding breast cancer tissues exhibit some gene expression properties reflecting those of the cancer subtypes, probably causing in some instances the recurrence after conservative therapies. Consequently, the importance of studying biomarkers in adjacent tissues to predict the risk of recurrence has been emphasized^37^. Recently, Aran *et al*.^38^ have conducted an exhaustive transcriptomic study in which compared the tumour, non-tumour adjacent tissues and healthy tissues obtained by post-mortem collection. In some instances, specific gene activation occurs in adjacent tissues. In our case, as *EPDR1* overexpression is compatible with a diminution of cell adhesion and in higher cell association with type I collagen fibres, increased expression in adjacent tissues may facilitate dissemination and the risk of metastasis.

At any rate, present results may be useful to consider the inclusion of *EPDR1* in panels of genes used to improve molecular subtyping of CRC and perhaps to consider the gene as an actionable therapeutic target.

## Methods

### Human CRC samples

*EPDR1* expression was first analysed in TissueScan cDNA arrays (OriGene, Rockville, USA), containing 5 cDNA samples from histologically normal mucosa, 10 from CRC stage I, 13 from stage II, 14 from stage III and 6 from stage IV. They corresponded to 21 males and 27 females aged from 36 to 92 years and an average of 68.5 ± 13.3 years. Clinical samples were obtained from the INCLIVA biobank. 101 paraffin-embedded CRC paired samples from stages I-IV patients were used; their clinicopathological characteristics are given in Supplementary Table S1. Stage IV samples were taken from the metastatic tumours. Written informed consents were obtained from all the patients before surgery and all the procedures were carried out in accordance with the Declaration of Helsinki and other relevant guidelines. The study protocol was approved and followed by the Ethical Committee of the University Hospital (Comité Ético de Investigación Clínica del Hospital Clinic Universitari de València, No. 2017/229).

### Cell culture

The human CRC cell lines SW48, RKO (Horizon Discovery), HCT116 (ATCC CCL-247), DLD1 (ATCC CCL-221) and its isogenic derivative D-Mut1 (a gift from Dr B. Vogelstein), were grown as previously described^27^. To guarantee the continued quality of these cell lines, a short tandem repeat DNA profiling of the cells was performed. Genomic DNA was extracted at different steps of the study and Bioidentity (Elche, Spain) monitored and confirmed the identity of the cell lines, complying with international standards of authentication (ANSI/ATCC). Preparations of genomic DNA were routinely checked to discard mycoplasma contamination, by using the Mycoplasma detection kit (Biotools, 90021), according to the manufacturer’s instructions. This checking was done in the Cell Culture Service of the UCIM (Central Research Unit, INCLIVA-Faculty of Medicine). For passive demethylation of DNA, RKO and SW48 cell lines were cultured for 72 h in the presence of 5 or 10 μM 5′-azacytidine.

### *EPDR1* knockdown and overexpression

DLD1 and HCT116 cells were cultured in 6 well plates to 50–60% confluence. Then 9 μl Lipofectamine RNAi Max (Invitrogen #1875252) per well and 20 nM of either scrambled siRNA (Qiagen 1027280) or *EPDR1* siRNAs, si1 (Qiagen SI04235721) and si2 (Qiagen SI03238053), were added following the instructions of the manufacturer. These transfections were carried out independently in three wells each. The cells were then incubated in CO_2_ atmosphere for several times at 37 °C. The efficiency of knockdown was checked by RT-qPCR and by western blotting. The effects of *EPDR1* knockdown were analysed after 48 and 96 h (Supplementary Fig. S2).

For transient overexpression of *EPDR1*, a plasmid containing the full-length cDNA of the canonical isoform 1, under the control of immediate-early cytomegalovirus promoter (Sino Biological, HG13665-ACG), was transfected into RKO and SW48 cells. An empty plasmid was used as control. For transfection, 3·10^5^ cells were cultured in 6 wells plate, and grown to 80% confluence. Then, a mixture of 4 μg of plasmid and 10 μl of Lipofectamine 2000 Reagent (Invitrogen, #11668–027), solved in Opti-MEM Medium (Thermo Fisher Scientific, #31985047) were added independently to each three wells. After 6 hours, this medium was removed and standard medium was added. The effects of *EPDR1* overexpresion were analysed after 72 h (Supplementary Fig. S7).

All the experiments described under this heading were repeated twice.

### Cell proliferation assays

Cell proliferation was measured by the 3-(4,5-dimethylthiazol-2-yl)-2,5-diphenyltetrazolium bromide (MTT) assay. After three independent transfections, either with scrambled siRNA or *EPDR1* siRNAs, cells were trypsinized and re-plated in quadruplicate (3,000 cells per well) in 96-well plates. At several times after seeding (0, 24, 48, 72 and 96 h), 10 μl of a 1 mg/ml solution of MTT in phosphate buffered saline were added to four wells each time. Proliferation was spectrophotometrically determined as recommended by the manufacturer. The results were given as the average of the four readings against time for two independent experiments.

### Cell migration and invasion assays

The assays were carried out in 12 well plates (Corning, Falcon, Cell Culture Inserts, with 8 μm pore size). For invasion assays, 50 μl matrigel (Cultrex Reduced Growth Factor BME, Type 2 PathClear, Sigma-Aldrich #3533–005–02) were loaded in the upper chamber prior to the introduction of the cells. For both, migration and invasion assays, 10^5^ cells were seeded in 100 μl foetal bovine serum-free McCoy medium in the upper chamber and the lower chamber contained McCoy medium either with foetal bovine serum or without it as a negative control. The assay was stopped 24 h (for migration) or 48 h (for invasion) after seeding. Cells adhering to the lower surface were fixed with 70% methanol and stained with 800 μl 0.2% crystal violet, counted under microscope in 6 different areas and the counting values were averaged. For wound healing assays, the Radius 24 cell migration assay kit (Cell Biolabs, CBA-125) was used. Every well was loaded with 8,000 cells and, after 24 h, the central gel layer was removed following the manufacturer’s instructions and the migration was checked by photographing the plates at 24, 48 and 72 h.

### Colony formation assay

After three independent transfections with either *EPDR1* siRNAs or scrambled siRNA, cells were trypsinized and re-plated at a density of 150 per well in 6-well plates. The procedure was repeated for each siRNA and for each cell line. After culturing for two weeks, the medium was withdrawn, the cells washed, fixed and stained as described above for migration and invasion assays. The total number of colonies (approximately more than 50 cells) was counted. A similar procedure was used for the overexpression assays but transfections were done either with p*EPDR1* of with control empty plasmid.

### Cell adhesion assays

Collagen type I-coated 60 mm dishes (Corning BioCoat Collagen I Cellware) were loaded with 30,000 cells suspended in McCoy’s medium and after 30 min the medium was withdrawn, the dishes washed with PBS and the adhering cells were counted as above.

### Flow cytometry analysis of cell cycle distribution

To better evaluate the effects of *EPDR1* knockdown on the cell cycle progression, the analysis was carried out at two different times. To do this, cells were re-plated 24 h after transfection. Then, 24 or 48 h later (48 and 72 h, respectively, after knocking-down) cells were trypsinized and incubated with a hypotonic propidium iodide solution at 4 °C for 12 h. To detect the level of apoptosis, the cells were treated with 7-aminoactinomycin D (7-AAD, Immuno step) and Annexin V (Immuno step, BB 10×) in Annexin buffer (Immuno step, ANXVFK-100T) 72 h after knocking-down. The samples were analysed in a Gallios Flow Cytometer (Beckman Coulter) following the manufacturer’s protocol. The distribution of cells was calculated by using either FloJo (TOMY Digital Biology) or ModFit TL (Verity Software House) software as described in the corresponding figures. Each experiment was repeated at least three times.

### Analysis of DNA methylation

Quantitative DNA methylation analysis was carried out using a MassARRAY platform (Agena Bioscience) as previously described^39^. The primers used, their localisation and the size of defined amplicons are given in the Supplementary Table S4.

### RT-qPCR analysis of the expression of *EPDR1*

The transcription level of the whole *EPDR1* gene was determined by RT-qPCR with the primers given in the Supplementary Table S5, which define an amplicon at the 5′ end of exon 6, common to all the isoforms. The level of isoform 2 was determined with primers from exon 4, unique to this isoform^27^ (Supplementary Table S5). The *ACTB* gene was used as loading control.

### Western blot analyses

Western blot analyses were carried out essentially as previously described^40^. Briefly, cultured cells were extracted with RIPA buffer (50 mM Tris-HCl pH 7.5, 150 mM NaCl, 1% Triton X-100, 0.1% SDS, 0.5% deoxycholic acid sodium salt (w/v)) supplemented with 2 μl/ml protease inhibitor cocktail (Sigma). Samples were sonicated and centrifuged at 13,000 × g for 30 min at 4 °C. Total protein was determined by a BCA protein assay kit (Thermo Fisher Scientific, 23225). Electrophoresis was carried out in 12% polyacrylamide gels and the transfer was done overnight. Immunoblots were visualized using either the ECL Western Blotting detection kit reagent (GE Healthcare) or SuperSignal West Femto (Thermo, #34095) and the ImageQuant LAAS 400 system (Healthcare Bio-Sciences). The antibodies used were: anti-EPDR1 (Abcam, ab-197932); anti-β-actin (Abcam, ab-8227). For a semi-quantitative determination of protein in the western blots, four grey values relative to the loading control were measured with ImageJ and averaged.

### Statistical analyses

The differential expression between tumour tissue and control was quantified using the difference of the observed expressions or their fold-change between tumour and adjacent tissue. The expression of the gene for both types of tissues was compared using a paired t-test. Multiple regression was used to study the relationship between the difference of the expressions or the fold-change and the predictors (StT, StN, StM and StStage). A stepwise variable selection was performed using the function stepAIC^41^. Survival functions, estimated using the Kaplan-Meier estimators, were plotted using survminer^42^. The relationship between the survival time and the covariables (expressions in tumour and normal tissue and the covariables StT, StN, StM and StStage) was studied using a Cox regression performed using the R package survival^43^. The survival functions were compared using the log-rank test when a single categorical covariable had been used.

The *in silico* analysis of The Cancer Genome Atlas (TCGA) data included the *EPDR1* differential expression analysis using edgeR bioconductor package and a survival analysis using the R package coin^44^. The expressions measured in the OriGene arrays for different stages were compared using a unilateral Kolmogorov-Smirnov test. A detailed description can be found in the Supplementary Material.

All the statistical analyses were performed using the software R^45^. The plots have been made using ggplot2^46^. Other R packages used in the analysis are rcompanion^47^ and xlsx^48^. The code with detailed comments can be found in Supplementary Material.

## Supplementary information

Supplementary Material.

## Data Availability

Materials, protocols and data reported in this paper are available from the corresponding author on reasonable request.
