# Supplementary material for: EPDR1 up-regulation in human colorectal cancer is related to staging and favours cell proliferation and invasiveness
Source: Sci Rep. 2020 Feb 28;10:3723. doi: 10.1038/s41598-020-60476-7 (PMC7048834; doi:10.1038/s41598-020-60476-7)
Supplement: Supplementary file 1 — Supplementary Material. [file 41598_2020_60476_MOESM1_ESM.pdf]

EPDR1 up-regulation in human colorectal cancer is related to staging and favours cell proliferation and invasiveness

F Gimeno-Valiente, ÁL Riffo-Campos, G Ayala, N Tarazona, V Gambardella, FM Rodríguez, M Huerta, C Martínez-Ciarpaglini, J Montón-Bueno, S Roselló, D Roda, A Cervantes, L Franco, G López-Rodas and J Castillo

Supplementary Material

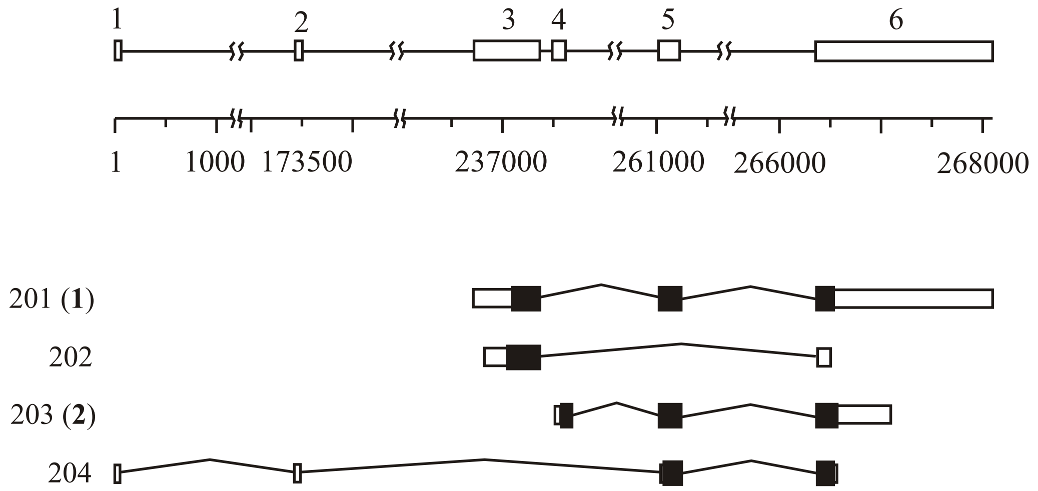

Supplementary Figure S1. Map of the *EPDR1* locus. The six exons of the gene are depicted as boxes and the scale gives the distance in base pairs to the first nucleotide of the first exon. The four isoforms resulting from alternative splicing are shown below. The potentially translatable exons are filled. The isoforms are numbered according to the ENSEMBL database and the two main isoforms are numbered in bold type in brackets. The latter numbering is followed in the main body of the paper.

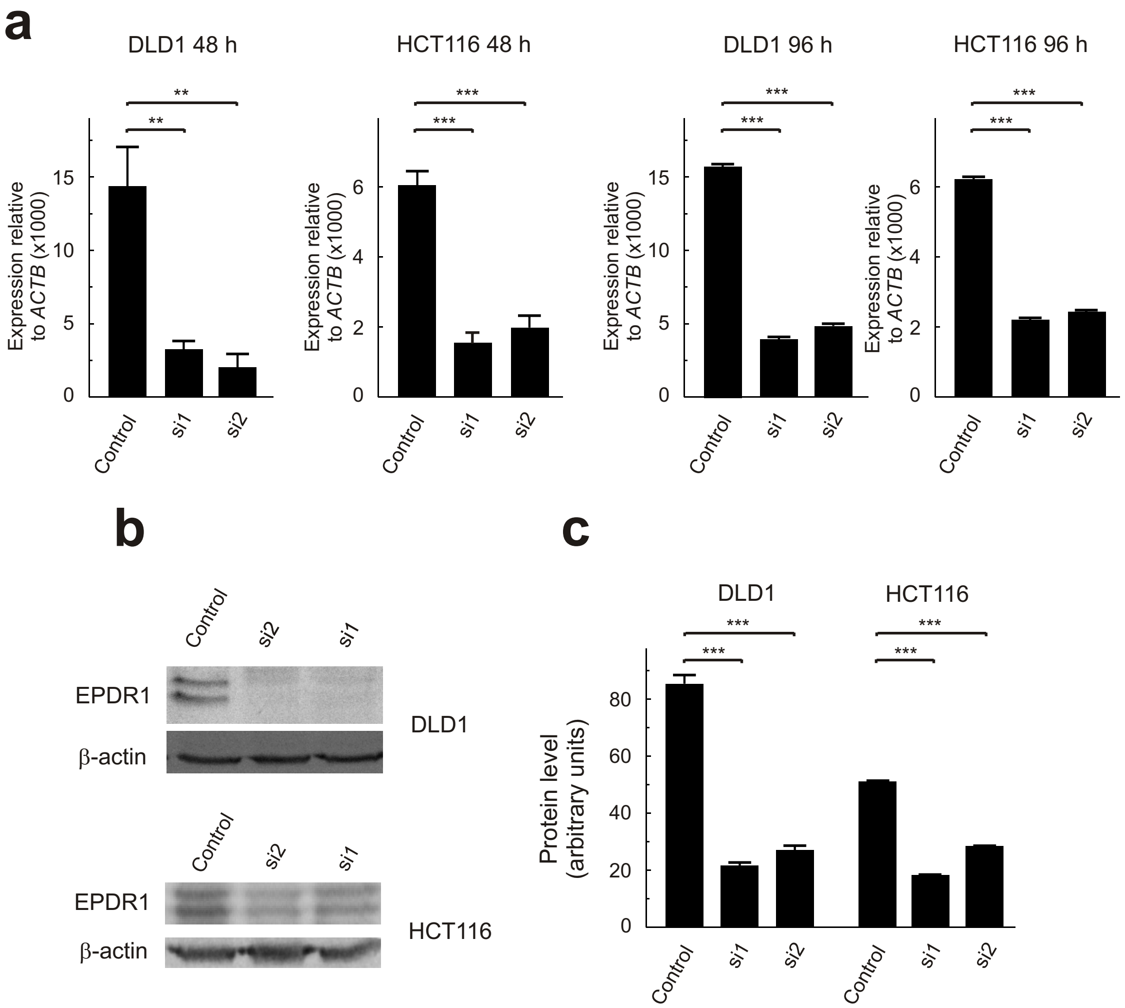

Supplementary Figure S2. Determination of the level of *EPDR1* silencing in DLD1 and HCT116 cells. (**a**) Transcription of the gene relative to that of the β-actin gene was measured by RT-qPCR after treating the cells either with scrambled siRNA (control) or with specific siRNAs for several times, as described under Materials and Methods. (**b**) Western blot showing the level of EPDR1 in DLD1 and HCT116 cells; β-actin was used as loading control. Ponceau-stained membranes were cropped according to the molecular weight of the corresponding proteins and the resulting strips were developed with the antibodies described under Materials and Methods. Note that in each panel the strips were independently developed but that the three lanes were simultaneously developed. In this way, the differences in exposure are irrelevant. (**c**) For a semiquantitative evaluation of the western blots in **b**, four gray values relative to the loading control were measured in every case and averaged. Statistical analysis was carried out with the Student's t-test relative to control. **, $p < 0.01$; ***, $p < 0.001$.

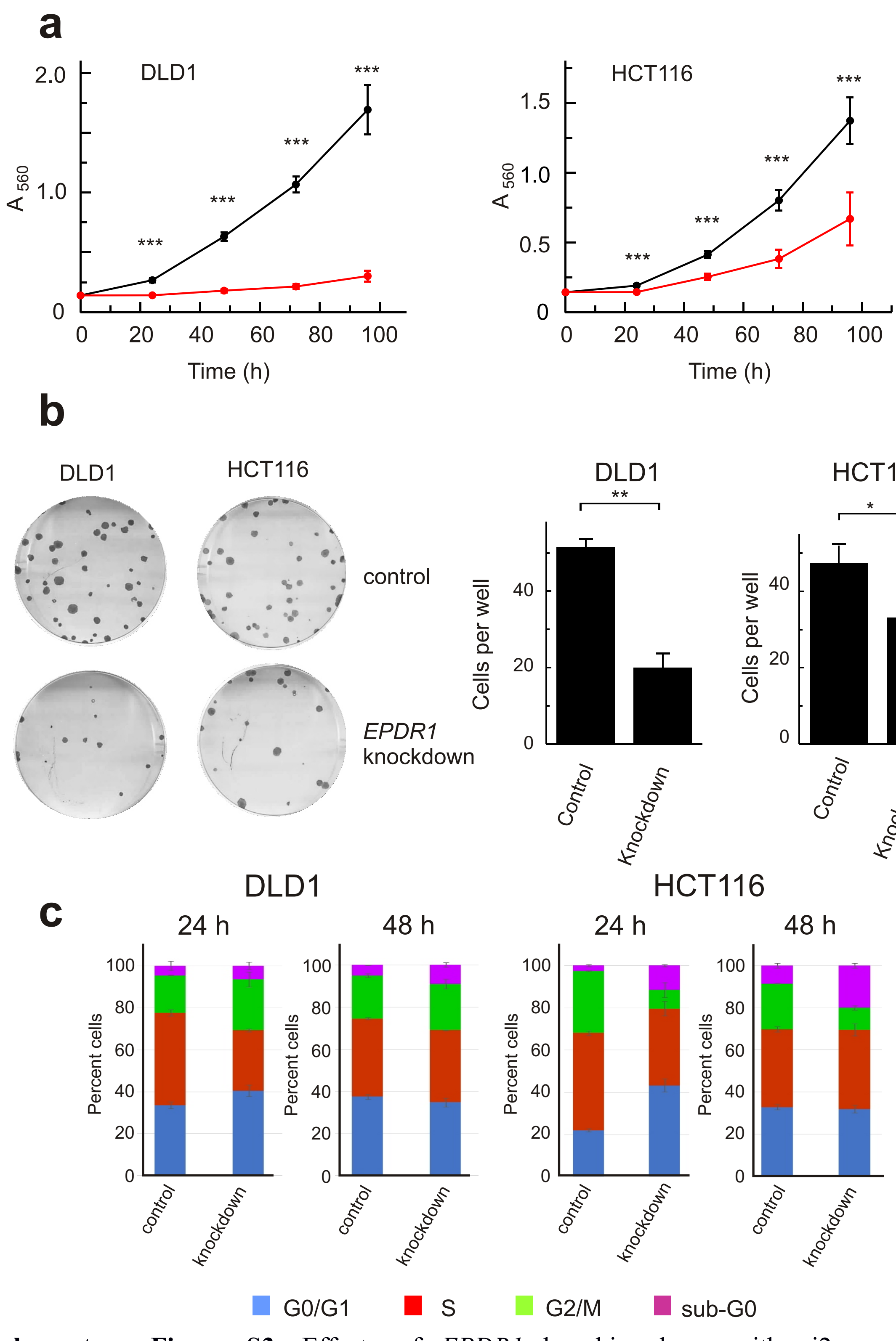

Supplementary Figure S3. Effects of *EPDR1* knocking-down with si2 on the growing of CRC cell lines. (**a**) MTT assay of cell proliferation in two *EPDR1*-expressing cell lines. The graph shows the results of a experiment, run in quintuplicate. The proliferation of cells treated with scrambled siRNA (black lines) is compared with that of cells transfected with p*EPDR1* (red lines). (**b**) Colony formation assays of cells treated with scrambled siRNA (control) and with si2 (*EPDR1* knockdown). Both the photograph of representative plates and the averaged quantification of 3 wells in each of 3 plates are given. (**c**) Flow-cytometry cell cycle analysis (ModFit software) of cells treated with scrambled siRNA (control) and with si2 (*EPDR1* knockdown). Statistical analysis was done by Student's t-test. *, $p < 0.05$; **, $p < 0.01$; ***, $p < 0.001$. .

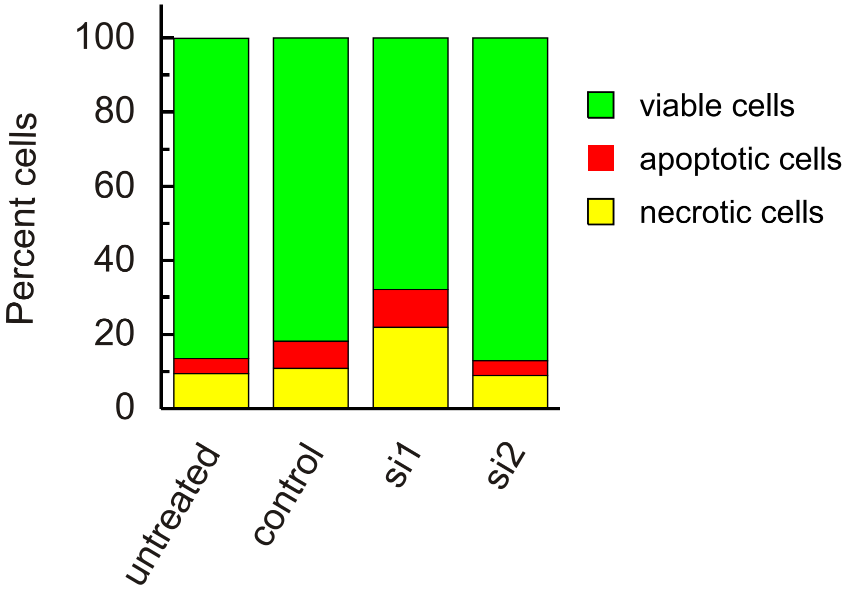

Supplementary Figure S4. Viability of untreated DLD1 cells and cells transfected with scrambled siRNA, or with the individual siRNAs, si1 and si2. The experiment was carried out in the presence of Annexin V/7-ADD. FloJo software was used to analyse the results.

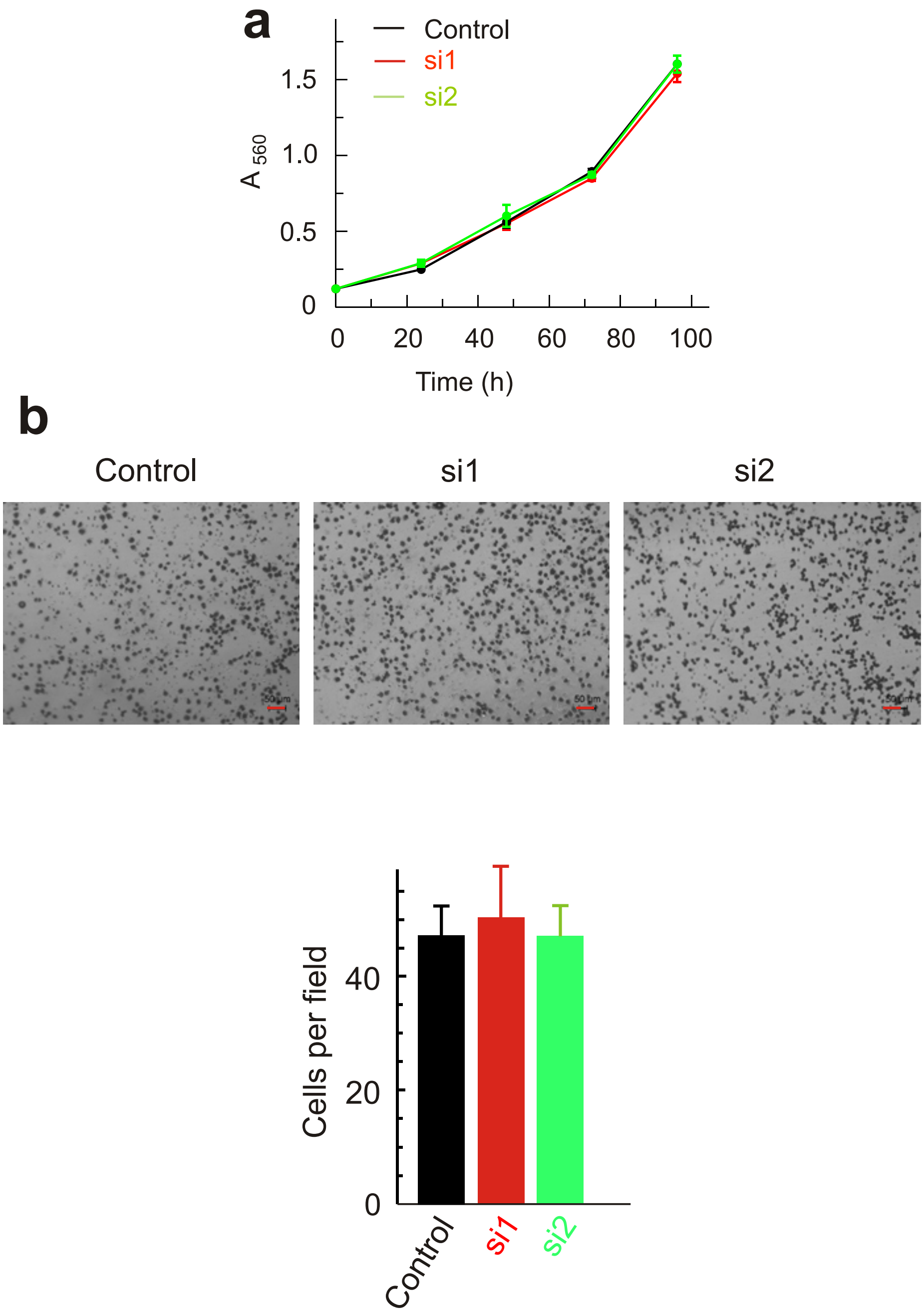

Supplementary Figure S5. Phenotypic effects of transfecting RKO cells with scrambled siRNA (control), si1 and si2. (**a**) MTT assay of cell proliferation. The graph shows the results of two independent experiments, each one with three independent transfections, as described under Materials and Methods. (**b**) Transwell migration assay of cells treated with the indicated siRNAs. Photographs of representavive plates are shown. Red bars correspond to 50 µm. Cells were counted under microscope in 6 different areas of every plate and the counting values were averaged and represented below.

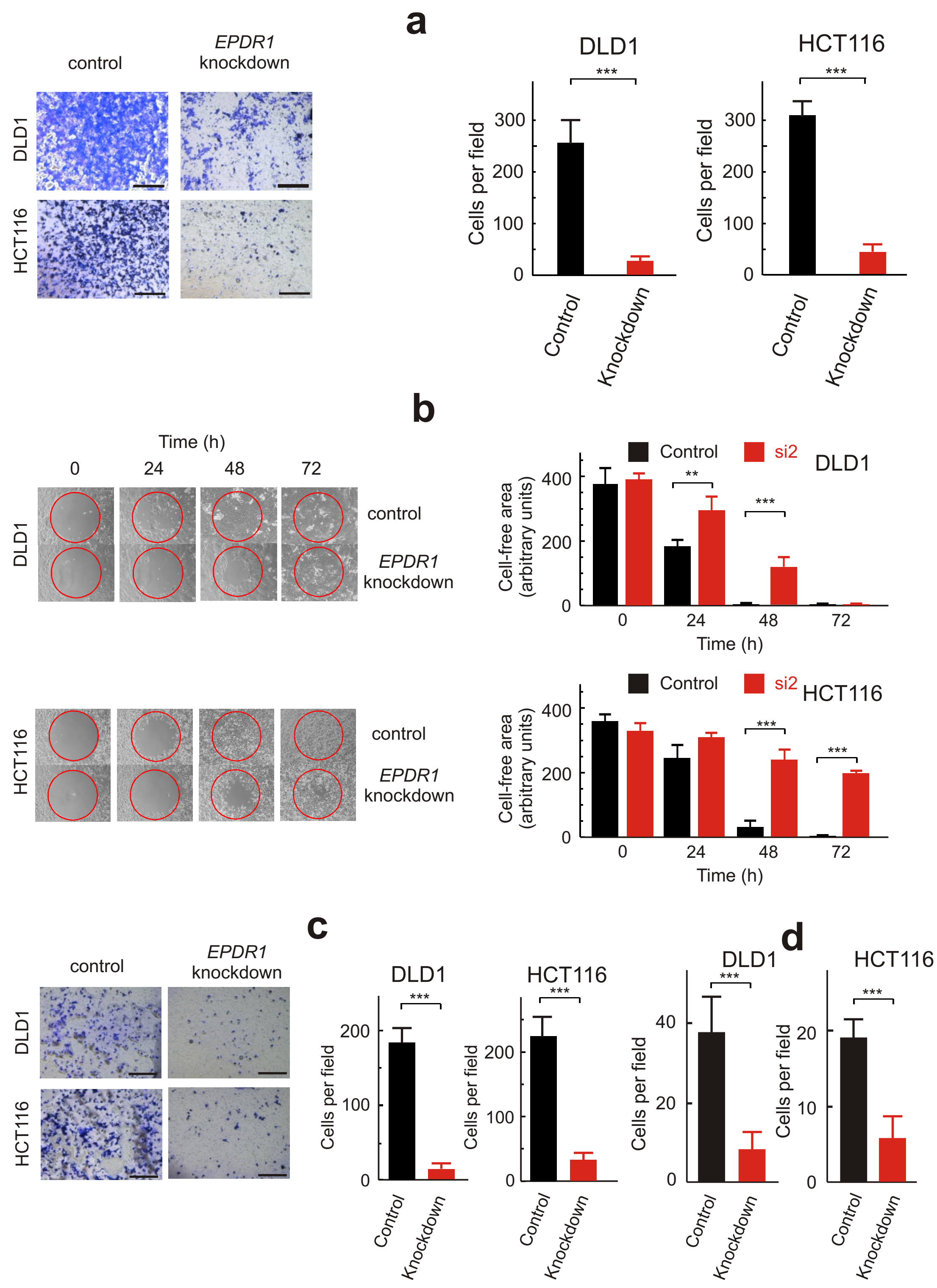

Supplementary Figure S6. Effects of *EPDR1* knocking-down with si2 on the migration, invasiveness and adhesion of CRC cell lines. (**a**) Transwell migration assay of cells treated with scrambled siRNA (control) and with si2 (*EPDR1* knockdown). A photograph of representative plates is shown (left). Cells were counted under microscope in 6 different areas of every plate and the counting values were averaged (right). (**b**) Wound-healing assay of cells treated with scrambled siRNA (control) and with si2 (*EPDR1* knockdown). A photograph of representative plates is shown at the left. The times shown were measured after removing the central gel layer (red circle). The average of three determination of the cell-free areas, as measured by ImageJ is shown at the right. (**c**) Assay carried out as in **a**, but through a Matrigel layer. (**d**) Effects of knocking-down the *EPDR1* gene on the adhesion of cells to type I collagen-coated plates (average of 6 determinations). The size bars in **a** and **c** correspond to 200 µm. Statistical analyses were carried out with the Student's t-test relative to control. **, $p < 0.01$; ***, $p < 0.001$.

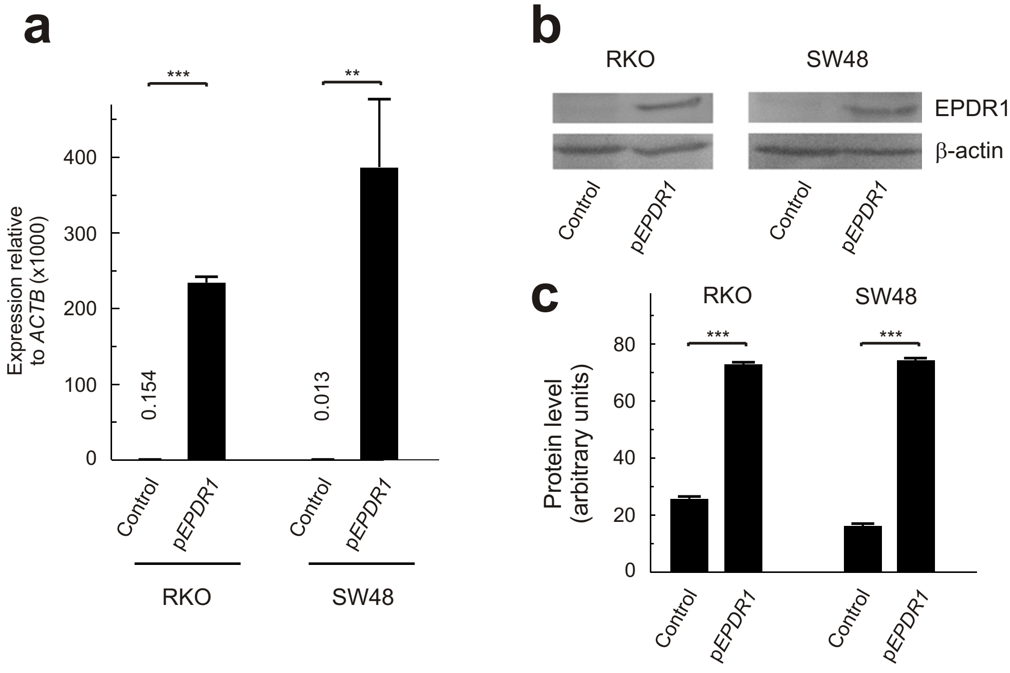

Supplementary Figure S7. Overexpression of *EPDR1* in RKO and SW48 cells. (**a**) Transcription of the gene relative to that of the β-actin gene was measured by RT-qPCR after transfecting the cells either with a plasmid carrying the whole *EPDR1* canonical isoform cDNA (p*EPDR1*) or with an empty plasmid (control), as described under Materials and Methods. The low expression values of the controls were given in numbers. (**b**) Western blot showing the level of EPDR1 in RKO and SW48 cells; β-actin was used as loading control. Ponceau-stained membranes were cropped according to the molecular weight of the corresponding proteins and the resulting strips were developed with the antibodies described under Materials and Methods. Note that in each panel the strips were independently developed but that the different lanes were simultaneously developed. In this way, the differences in exposure are irrelevant. (**c**) For a semiquantitative evaluation of the western blots in **b**, four gray values relative to the loading control were measured in every case and averaged. Statistical comparisons were done by Student's t-test. **, $p < 0.01$;***, $p < 0.001$.

Supplementary Table S1. Clinicopathological characteristics of the patients' cohort (n=101).

Patients' characteristics

Median Age	73<br>(range 37-96)	n (%)
Sex	Male	61 (60.3%)
	Female	40 (39.6%)
T stage	T1	9 (8.9 %)
	T2	17 (16.8%)
	T3	30 (29.7%)
	T4	26 (25.7%)
	nd	19 (18.8%)
N stage	N0	47 (46.5%)
	N1	22 (21.8%)
	N2	13 (12.9%)
	nd	19 (18.8%)
M stage	M0	82 (81.2%)
	M1	19 (18.8%)
AJCC Stage	I	12 (11.9%)
	II	35 (34.7%)
	III	35 (34.7%)
	IV	19 (18.8%)
Vascular invasion	Yes	35 (34.7%)
	No	56 (55.4%)
	nd	10 (9.9%)
Peritoneal invasion	Yes	30 (29.7%)
	No	61 (60.4%)
	nd	10 (9.9%)

The age of the patients range from 37 to 96 years, with a mean age of 73 years.
nd: not determined in the histological analysis.

Supplementary Table S2. *In silico* analysis of *EPDR1* expression in the paired samples of CRC recovered from TCGA database.

Cohort	No. of patients	FC*	P-value
All the patients	50	1.705	$3.631 \cdot 10^{-5}$
Stage I	8	1.421	0.328
Stage II	24	1.614	0.0068
Stage III	9	2.645	0.0038
Stage IV	9	1.743	0.0176

*This column gives the fold change of gene expression in tumour tissue relative to the adjacent normal mucosa.

Supplementary Table S3. *In silico* analysis of *EPDR1* expression in the paired samples of CRC recovered from the Kim *et al.* (ref. 28 in the main text) database (https://www.ncbi.nlm.nih.gov/bioproject/218851).

Paired samples	No. of patients	FC*	P-value
Primary tumour *vs* normal mucosa	18	2.150	$7.99 \cdot 10^{-5}$
Metastasis *vs* normal colonic mucosa	18	2.518	$4.04 \cdot 10^{-6}$

*This column gives the fold change of gene expression in tumour tissue relative to the normal mucosa adjacent to the primary tumour.

Supplementary Table S4. Sequence of the primers used to analyse DNA methylation in *EPDR1* intron 3, exon 3 (non-translatable and translatable regions) and intron 3.

Covered region	Primers (5'-3')		Amplicon size (bp)
	Forward	Reverse	
Intron 2	TAGGTTGGATGGAGTGTAGTGGTAT	ACTCCCATTCCCAATAAAAAATCTA	347
Non-translat. Exon 3	TTTAGATTTTTTATTGGGAATGGGA	TCAACAAATAATCACACAAAAAAAA	226
	TTTTTTTTGTGTGATTATTTGTTGA	AAACCAAAATACTTCCTATCCCTTC	338
Translat. Exon 3-Intron 3	GTTTTTTTGGGTTTGGGTTTGT	AACCTATACCTCTTACAAAAAATCAAC	326

Supplementary Table S5. Sequence of the primers used for the RT-qPCR analysis of the expression of whole *EPDR1* and of its isoform 2.

	**Primers (5'-3')**		**Amplicon size (bp)**
	**Forward**	**Reverse**	
Wole gene	TGAAACCTGGATTGGCATCTATAC	TGTAGTTTATGGTAAAGGTTTCCTG	71
Isoform 2	GAGGAGGGTCTCTTGGGGAT	GCTGGGTGTTACTGAGTCCC	96
ACTB (control)	GTGCTATCCCTGTACGCCTC	GAGGGCATACCCCTCGTAGA	99

Statistical analysis

Packages

```
pacman::p_load(survival,survminer,ggplot2,edgeR,SummarizedExperiment,MASS,
               coin,rcompanion,plyr,xlsx)
```

Incliva data

This section load and describe our own data named **Incliva data** from now on.

Data

First we load the data set from the directory `../data/`.

```
load("../data/yt.rda")
levels(yt$StM) = c("0","1","1")
```

Let us define a categorical version of the variable `Age`.

```
Age70 = cut(yt$Age,breaks=c(0,70,Inf))
table(Age70)
```

```
## Age70
##   (0,70] (70,Inf]
##       37       56
```

```
yt = data.frame(yt,Age70)
```

Descriptives

```
summary(yt[,-27]) ## Removing the `Notes` variables in the summary
```

```
##       NHC             DoBirth               Gender          Location
##  Min.   :  3376   Min.   :1922-05-13   Female:37   Sigmoid   :38
##  1st Qu.:366022   1st Qu.:1938-08-12   Male  :60   Cecum     :13
##  Median :486181   Median :1946-04-06               Ascending :10
##  Mean   :483835   Mean   :1945-06-12               Descending: 8
##  3rd Qu.:623617   3rd Qu.:1951-01-06               Transverse: 7
##  Max.   :769690   Max.   :1981-03-17               (Other)   :15
##                                                    NA's      : 6
##    StT       StN      StM     StStage     LNAff          LNRes
##  1   : 9   0   :51   0:80   1:22    Min.   : 0.0   Min.   : 7.00
##  2   :19   1   :28   1:17   2:25    1st Qu.: 0.0   1st Qu.:14.00
##  3   :35   2   :15          3:33    Median : 0.0   Median :17.00
##  4   :32   NA's: 3          4:17    Mean   : 1.5   Mean   :20.33
##  NA's: 2                            3rd Qu.: 2.0   3rd Qu.:26.00
##                                     Max.   :10.0   Max.   :53.00
##                                     NA's   :3      NA's   :5
```

```
##         MSI.x               MutSt             MutNRAS
##  MLH1 y PMS2: 7   No (wild type):16   No (wild type):21
##  MSH2       : 0   Undetermined  : 2   Undetermined  : 3
##  No (all +) :78   Yes           : 7   Yes           : 1
##  PMS2       : 0   NA's          :72   NA's          :72
##  NA's       :12
##
##
##            MutRAF   MutCDX2              HisType   HisGrade.x InvVasc.x
##  No (wild type):23   0   : 1   Adenocarcinoma  :88   High: 7    No  :55
##  Undetermined  : 3   0,5 : 3   Medullary       : 0   Low :86    Yes :28
##  Yes           : 1   2   : 6   Mucinous        : 9   NA's: 4    NA's:14
##  NA's          :70   3   :69   Undifferentiated: 0
##                      NA's:18
##
##
##  InvPeri.x  TBud.x          ClObsPer      DoSurg
##  No  :60   No  :28   None       :97   Min.   :2006-06-01
##  Yes :23   Yes :28   Obstruction: 0   1st Qu.:2016-03-22
##  NA's:14   NA's:41                    Median :2016-07-21
##                                       Mean   :2016-07-01
##                                       3rd Qu.:2017-03-07
##                                       Max.   :2017-10-31
##                                       NA's   :2
##       DoLF             LFState      LFState2           DoD
##  Min.   :2016-11-14   Alive:91   Min.   :0.00000   Min.   :2016-11-14
##  1st Qu.:2017-12-05   Dead : 6   1st Qu.:0.00000   1st Qu.:2017-09-19
##  Median :2018-02-05              Median :0.00000   Median :2017-10-28
##  Mean   :2018-01-01              Mean   :0.05155   Mean   :2017-09-04
##  3rd Qu.:2018-02-27              3rd Qu.:0.00000   3rd Qu.:2017-11-19
##  Max.   :2018-03-27              Max.   :1.00000   Max.   :2018-02-02
##  NA's   :2                                         NA's   :92
##        ID            exprs.t           exprs.nt          T
##  Min.   :  1.00   Min.   :   0.110   Min.   :  0.110   1   : 9
##  1st Qu.: 25.00   1st Qu.:   9.344   1st Qu.:  0.110   2   :20
##  Median : 49.00   Median :  32.837   Median :  2.788   3   :35
##  Mean   : 49.48   Mean   :  81.429   Mean   : 17.974   4   :30
##  3rd Qu.: 74.00   3rd Qu.:  62.112   3rd Qu.: 12.392   NA's: 3
##  Max.   :101.00   Max.   :1598.285   Max.   :458.300
##
##        M              N          MSI.y         HisGrade.y InvVasc.y
##  Min.   :0.0000   0   :52   Min.   :0.00000   High: 7    No  :55
##  1st Qu.:0.0000   1   :28   1st Qu.:0.00000   Low :86    Yes :36
##  Median :0.0000   2   :14   Median :0.00000   NA's: 4    NA's: 6
##  Mean   :0.1649   NA's: 3   Mean   :0.08235
##  3rd Qu.:0.0000             3rd Qu.:0.00000
##  Max.   :1.0000             Max.   :1.00000
##                             NA's   :12
##  InvPeri.y  TBud.y     BirthDate               Age         Sex
##  No  :61   No  :29   Min.   :1922-05-13   Min.   :37   Female:35
##  Yes :30   Yes :29   1st Qu.:1939-01-04   1st Qu.:67   Male  :58
##  NA's: 6   NA's:39   Median :1946-04-06   Median :72   NA's  : 4
##                      Mean   :1945-07-01   Mean   :73
##                      3rd Qu.:1951-03-05   3rd Qu.:79
```

```
##                      Max.   :1981-03-17   Max.   :96
##                      NA's   :4            NA's   :4
##       Age70
##  (0,70]  :37
##  (70,Inf]:56
##  NA's    : 4
##
##
##
##
```

Note that for `exprs.t` and `exprs.nt` the means are clearly larger than the medians i.e. there is skewness to the right.

Is there a significant difference between expressions?

The usual approach is to compare the paired observed expression using a paired `t.test`.

```
t.test(yt$exprs.t,yt$exprs.nt,paired=TRUE)
```

```
##
##  Paired t-test
##
## data:  yt$exprs.t and yt$exprs.nt
## t = 2.8765, df = 96, p-value = 0.004955
## alternative hypothesis: true difference in means is not equal to 0
## 95 percent confidence interval:
##   19.66655 107.24319
## sample estimates:
## mean of the differences
##                63.45487
```

The means and standard deviation of each variable are

```
mean(yt$exprs.t)
```

```
## [1] 81.42855
```

```
sd(yt$exprs.t)
```

```
## [1] 210.6317
```

```
mean(yt$exprs.nt)
```

```
## [1] 17.97368
```

```
sd(yt$exprs.nt)
```

```
## [1] 53.57996
```

There is a significant difference between the means.

```
df= data.frame(Exprs.tumor = yt$exprs.t,Exprs.control = yt$exprs.nt)
df1 = reshape2::melt(df)
```

```
## No id variables; using all as measure variables
```

```
levels(df1[,"variable"]) = c("t","nt")
names(df1) = c("tissue","expression")
```

```
png("figures/incliva_bp_jitter_200.png")
ggplot(df1,aes(x=tissue,y = expression)) + geom_boxplot() +
    geom_jitter(width = 0.2) +  ylim(c(0,200)) + theme_bw() + xlab("") + ylab("")
dev.off()

png("figures/incliva_bp_jitter.png")
ggplot(df1,aes(x=tissue,y = expression)) + geom_jitter(width = 0.2)+ theme_bw()
dev.off()

png("figures/incliva_density_color.png")
ggplot(df1,aes(x=expression,color=tissue)) + geom_density()+ theme_bw()
dev.off()

png("figures/incliva_density_color_0_200.png")
ggplot(df1,aes(x=expression,color=tissue)) + geom_density()+ theme_bw()+
  xlim(0,200)
dev.off()

png("figures/incliva_density_color_0_150.png")
ggplot(df1,aes(x=expression,color=tissue)) + geom_density()+ theme_bw()+
  xlim(0,150)
dev.off()

mu = ddply(df1, "tissue", summarise, grp.mean=mean(expression))

png("figures/incliva_density_color_means.png")
ggplot(df1,aes(x=expression,color=tissue)) + geom_density() +
    geom_vline(data=mu, aes(xintercept=grp.mean))+ theme_bw()
dev.off()

png("figures/incliva_density_grey_means.png")
ggplot(df1,aes(x=expression,color=tissue)) + geom_density() +
    geom_vline(data=mu, aes(xintercept=grp.mean, color=tissue),
               linetype="dashed") + scale_color_grey()+ theme_bw()
dev.off()
```

Quantification of the differences between expressions in both conditions

This difference could be quantified as a difference of the expressions or as their fold-change.

```
exprs.dif = yt$exprs.t - yt$exprs.nt
fc = (yt$exprs.t+.5)/(yt$exprs.nt+.5) ## Adding 0.5 to avoid null values
yt = data.frame(yt,exprs.dif,fc)
```

We are going to describe these differences and the each variable separately by taking into account some important phenotypic covariables. First we define some functions needed to perform these descriptive studies.

```
summary0 = function(x0)  aa = sum(!is.na(x0))

summary1 = function(x0,mu0=0){
    aa = t.test(x0,mu=mu0)
    aa$conf.int[1]
}
```

```r
summary2 = function(x0,mu0=0){
    aa = t.test(x0,mu=mu0)
    aa$conf.int[2]
}

summary3 = function(x0,mu0=0){
  aa = t.test(x0,mu=mu0)
    aa$p.value
}

summary4 = function(x0,x1){
  aa = t.test(x0,x1,na.rm=TRUE)
    aa$p.value
}

## When there is  no mean `mu0` we use the mean of the first level of `y0`
gen.descriptive = function(x0,y0,mu0=0,x1=NULL){
  if(nlevels(as.factor(y0)) == 2){
    if(is.null(x1))
    result = data.frame(number=aggregate(x0,by= list(y0),FUN = summary0),
                        mean=aggregate(x0,by= list(y0),FUN = mean),
                        lower_ci=aggregate(x0,by= list(y0),FUN = summary1,mu0)$x,
                        upper_ci = aggregate(x0,by= list(y0),FUN = summary2,mu0)$x,
                        p.value=aggregate(x0,by= list(y0),FUN = summary3,mu0)$x)
    else
      result = data.frame(number=aggregate(x0,by= list(y0),FUN = summary0),
                       mean= aggregate(x0,by= list(y0),FUN = mean),
                       lower_ci=aggregate(x0,by= list(y0),FUN = summary1,mu0)$x,
                       upper_ci = aggregate(x0,by= list(y0),FUN = summary2,mu0)$x,
                       p.value=aggregate(x0,by= list(y0),FUN = summary4,x1)$x)

        result = result[,-c(1,3)]
    colnames(result) = c("number","mean","lower CI","upper CI","p.value")
    per_group_results = result
    df = na.omit(data.frame(x0,y0))
    aa = t.test(x0 ~ as.factor(y0),data=df)
    global_results = data.frame(statistic= aa$statistic, p.value = aa$p.value)
    output = list(Per_group_descriptives = per_group_results,Global_descriptives = global_results)
  }
   if(nlevels(as.factor(y0)) > 2){
         if(is.null(x1))
    result = data.frame(number=aggregate(x0,by= list(y0),FUN = summary0),
                        mean=aggregate(x0,by= list(y0),FUN = mean),
                       lower_ci=aggregate(x0,by= list(y0),FUN = summary1,mu0)$x,
                       upper_ci=aggregate(x0,by= list(y0),FUN = summary2,mu0)$x,
                       p.value=aggregate(x0,by= list(y0),FUN = summary3,mu0)$x)
    else
      result = data.frame(aggregate(x0,by= list(y0),FUN = summary0),
                       mean= aggregate(x0,by= list(y0),FUN = mean),
                       lower_ci=aggregate(x0,by= list(y0),FUN = summary1,mu0)$x,
                       upper_ci=aggregate(x0,by= list(y0),FUN = summary2,mu0)$x,
                       p.value=aggregate(x0,by= list(y0),FUN = summary4,x1)$x)
```

```
    result = result[,-c(1,3)]
    colnames(result) = c("number","mean","lower CI","upper CI","p.value")
    per_group_results = result
    df = na.omit(data.frame(x0,y0))
    aa1 = aov(x0 ~y0,data=df)
    aa2 = aov(x0 ~1 ,data=df)
    bb = anova(aa1,aa2)
    global_results = data.frame(statistic = bb[2,5],p.value= bb[2,6])
    output = list(Per_group_descriptives = per_group_results,
                  Global_descriptives = global_results)
   }
  output
}
```

Descriptives using sign and Mann-Whitney tests.

```
summary_median0 = function(x0)  aa = sum(!is.na(x0))

summary_median1 = function(x0) min(x0,na.rm=TRUE)

summary_median2 = function(x0) max(x0,na.rm=TRUE)

summary_median3 = function(x0,md0=0){
  aa = SIGN.test(x0,md=md0)
    aa$p.value
}

summary_median4 = function(x0,x1){
  aa = wilcox.test(x1,x0)
    aa$p.value
}

## When there is  no mean `mu0` we use the mean of the first level of `y0`
gen.descriptive.quantile = function(x0,y0,x1=NULL){
  if(nlevels(as.factor(y0)) == 2){
    if(is.null(x1))
result = data.frame(number=aggregate(x0,by= list(y0),FUN = summary_median0),
median=aggregate(x0,by= list(y0),FUN = median,na.rm=TRUE),
lower_range=aggregate(x0,by= list(y0),FUN = min,na.rm=TRUE)$x,
upper_range = aggregate(x0,by= list(y0),FUN = max,na.rm=TRUE)$x,
p.value=aggregate(x0,by= list(y0),FUN = summary_median4,x1)$x)
    else
result = data.frame(number=aggregate(x0,by= list(y0),FUN = summary_median0),
median = aggregate(x0,by= list(y0),FUN = median,na.rm=TRUE),
min=aggregate(x0,by= list(y0),FUN = min)$x,
max = aggregate(x0,by= list(y0),FUN = max)$x,
p.value=aggregate(x0,by= list(y0),FUN = summary_median4,x1)$x)
        result = result[,-c(1,3)]
    colnames(result) = c("number","median","min","max","p.value")
    per_group_results = result
    output = list(Per_group_descriptives = per_group_results)
  }
   if(nlevels(as.factor(y0)) > 2){
        if(is.null(x1))
```

```
  result = data.frame(number=aggregate(x0,by= list(y0),FUN = summary_median0),
median=aggregate(x0,by= list(y0),FUN = median),
min=aggregate(x0,by= list(y0),FUN = min)$x,
max=aggregate(x0,by= list(y0),FUN = max)$x,
p.value=aggregate(x0,by= list(y0),FUN = summary_median4,x1)$x)
    else
result = data.frame(aggregate(x0,by= list(y0),FUN = summary_median0),
median= aggregate(x0,by= list(y0),FUN = median),
min=aggregate(x0,by= list(y0),FUN = min)$x,
max=aggregate(x0,by= list(y0),FUN = max)$x,
p.value=aggregate(x0,by= list(y0),FUN = summary_median4,x1)$x)

    result = result[,-c(1,3)]
    colnames(result) = c("number","median","min","max","p.value")
    per_group_results = result
    output = list(Per_group_descriptives = per_group_results)
   }
  output
}
```

Relationship between the *difference of expression* with respect to covariables

First some descriptives of the variable `exprs.dif` with respect to the important covariables.

```
gen.descriptive(exprs.dif,yt$StT,mu0 = 0)
```

```
## $Per_group_descriptives
##   number      mean   lower CI  upper CI     p.value
## 1      9  36.45733   4.558807  68.35585 0.029918404
## 2     19 171.33239 -28.112486 370.77726 0.087865149
## 3     35  45.83259 -14.276902 105.94209 0.130507333
## 4     32  29.69735   7.884349  51.51034 0.009231989
##
## $Global_descriptives
##   statistic   p.value
## 1  1.961692 0.1253138
```

```
gen.descriptive(exprs.dif,yt$StN,mu0 = 0)
```

```
## $Per_group_descriptives
##   number     mean   lower CI  upper CI    p.value
## 1     51 44.43663   1.447281  87.42598 0.04303839
## 2     28 95.26540 -20.815976 211.34678 0.10373095
## 3     15 80.23430 -58.817024 219.28562 0.23623115
##
## $Global_descriptives
##   statistic   p.value
## 1 0.5158778 0.5987116
```

```
gen.descriptive(exprs.dif,yt$StM,mu0 = 0)
```

```
## $Per_group_descriptives
##   number      mean  lower CI upper CI      p.value
## 1     80  33.08415  22.49910  43.6692 2.202575e-08
## 2     17 206.37591 -48.49823 461.2500 1.053596e-01
```

```
##
## $Global_descriptives
##   statistic   p.value
## t -1.439939 0.1690903
```

```r
gen.descriptive(exprs.dif,yt$StStage,mu0 = 0)
```

```
## $Per_group_descriptives
##   number      mean   lower CI  upper CI      p.value
## 1     22  40.77637   9.962813  71.58993 1.194531e-02
## 2     25  28.83891  13.302361  44.37546 8.067088e-04
## 3     33  31.17210  18.413636  43.93056 2.126162e-05
## 4     17 206.37591 -48.498233 461.25005 1.053596e-01
##
## $Global_descriptives
##   statistic    p.value
## 1  3.190921 0.02723938
```

```r
gen.descriptive(exprs.dif,yt$InvVasc.y,mu0 = 0)
```

```
## $Per_group_descriptives
##   number      mean lower CI  upper CI    p.value
## 1     55  46.46583 8.908856  84.02281 0.01626987
## 2     36 108.64531 5.916894 211.37372 0.03879799
##
## $Global_descriptives
##   statistic   p.value
## t -1.152357 0.2552995
```

```r
gen.descriptive(exprs.dif,yt$InvPeri.y,mu0 = 0)
```

```
## $Per_group_descriptives
##   number      mean    lower CI  upper CI     p.value
## 1     61  45.16897 11.34462437  78.99331 0.009714654
## 2     30 123.71816  0.02664703 247.40968 0.049953835
##
## $Global_descriptives
##   statistic   p.value
## t -1.250832 0.2196329
```

```r
gen.descriptive(exprs.dif,yt$HisGrade.y,mu0 = 0)
```

```
## $Per_group_descriptives
##   number     mean  lower CI upper CI     p.value
## 1      7 23.46520 -63.26505 110.1954 0.532545211
## 2     86 71.46981  22.63547 120.3042 0.004614471
##
## $Global_descriptives
##   statistic   p.value
## t -1.113204 0.2858808
```

```r
gen.descriptive(exprs.dif,yt$Sex,mu0 = 0)
```

```
## $Per_group_descriptives
##   number     mean   lower CI  upper CI    p.value
## 1     35 93.77068 -13.102739 200.64410 0.08350203
## 2     58 41.97684   5.124597  78.82909 0.02630973
##
```

```
## $Global_descriptives
##   statistic   p.value
## t 0.9296041 0.3578315
```

```
gen.descriptive(exprs.dif,Age70,mu0 = 0)
```

```
## $Per_group_descriptives
##   number     mean  lower CI  upper CI      p.value
## 1     37 38.15742 19.460758  56.85408 0.0002005032
## 2     56 76.87154  1.794001 151.94908 0.0449488441
##
## $Global_descriptives
##   statistic   p.value
## t  -1.00346 0.3195695
```

Relationship between the *fold change* with respect to covariables

The analogous description for the fold change.

```
gen.descriptive(fc,yt$StT,mu0 = 1)
```

```
## $Per_group_descriptives
##   number      mean   lower CI  upper CI     p.value
## 1      9  32.00304   5.920048  58.08603 0.025410436
## 2     19 247.12131 -81.637571 575.88019 0.133170453
## 3     35  65.05691 -24.580329 154.69416 0.155587553
## 4     32  41.00470  17.528742  64.48066 0.001530293
##
## $Global_descriptives
##   statistic   p.value
## 1  1.674631 0.1779961
```

```
gen.descriptive(fc,yt$StN,mu0 = 1)
```

```
## $Per_group_descriptives
##   number     mean    lower CI upper CI    p.value
## 1     51  59.1938   -5.056601 123.4442 0.07486824
## 2     28 135.3189  -55.382066 326.0199 0.15991106
## 3     15 117.5614 -100.124641 335.2474 0.27003179
##
## $Global_descriptives
##   statistic   p.value
## 1 0.4716616 0.6254814
```

```
gen.descriptive(fc,yt$StM,mu0 = 1)
```

```
## $Per_group_descriptives
##   number     mean  lower CI  upper CI      p.value
## 1     80  31.1996  19.00078  43.39841 4.493264e-06
## 2     17 358.9400 -41.32433 759.20423 7.620424e-02
##
## $Global_descriptives
##   statistic   p.value
## t -1.734886 0.1019388
```

```r
gen.descriptive(fc,yt$StStage,mu0 = 1)
```

```
## $Per_group_descriptives
##   number      mean   lower CI  upper CI     p.value
## 1     22  31.70712   8.023655  55.39058 0.013518769
## 2     25  28.26184   3.607884  52.91579 0.031634442
## 3     33  33.08682  14.125034  52.04861 0.001607364
## 4     17 358.93995 -41.324330 759.20423 0.076204244
##
## $Global_descriptives
##   statistic     p.value
## 1  4.700758 0.004224199
```

```r
gen.descriptive(fc,yt$InvVasc.y,mu0 = 1)
```

```
## $Per_group_descriptives
##   number      mean   lower CI upper CI    p.value
## 1     55  53.05683  -5.941466 112.0551 0.08254214
## 2     36 156.96368 -10.639118 324.5665 0.06718274
##
## $Global_descriptives
##   statistic   p.value
## t -1.185524 0.2421739
```

```r
gen.descriptive(fc,yt$InvPeri.y,mu0 = 1)
```

```
## $Per_group_descriptives
##   number      mean   lower CI upper CI    p.value
## 1     61  51.94861  -1.210545 105.1078 0.05998800
## 2     30 179.99844 -21.924878 381.9218 0.08019085
##
## $Global_descriptives
##   statistic   p.value
## t -1.252405 0.2191547
```

```r
gen.descriptive(fc,yt$HisGrade.y,mu0 = 1)
```

```
## $Per_group_descriptives
##   number     mean  lower CI  upper CI   p.value
## 1      7 25.25217 -18.68333  69.18768 0.2255101
## 2     86 97.63451  19.24079 176.02823 0.0162991
##
## $Global_descriptives
##   statistic    p.value
## t -1.670717 0.09883778
```

```r
gen.descriptive(fc,yt$Sex,mu0 = 1)
```

```
## $Per_group_descriptives
##   number      mean   lower CI upper CI    p.value
## 1     35 147.79857 -27.106519 322.7037 0.09719049
## 2     58  51.95237  -1.886676 105.7914 0.06315467
##
## $Global_descriptives
##   statistic p.value
## t  1.062987 0.29405
```

```r
gen.descriptive(fc,Age70,mu0 = 1)
```

```
## $Per_group_descriptives
##   number      mean  lower CI  upper CI     p.value
## 1     37  29.47085 12.937881  46.00382 0.001285561
## 2     56 126.71011  6.371382 247.04884 0.040929123
##
## $Global_descriptives
##   statistic   p.value
## t -1.604641 0.1140981
```

Relationship between the expression in tumor and no tumor with respect to covariables

gen.descriptive.quantile = function(x0,y0,md0=0,x1=NULL){

First a function needed.

```r
do.table1temp = function(yt,name0,name1,type=c("mean","median")){
  x0 = yt[,name0]
  y0 = yt[,name1]
  if(type == "mean")
  gd = gen.descriptive(x0,y0,x1=x0[y0==levels(y0)[1]])
  if(type == "median")
    gd = gen.descriptive.quantile(x0,y0,x1=x0[y0==levels(y0)[1]])

  table.temp =   gd$Per_group_descriptives
  rownames(table.temp) = paste(name1,levels(y0))
  table.temp
}

do.table1 = function(yt,name0,all.name1,type){
  table1 = NULL
for(name1 in all.name1){
  table.temp =  do.table1temp(yt,name0=name0,name1=name1,type)
  table1 = rbind(table1,table.temp)
}
colnames(table1) = c("No. of patients","Expression level","Lower CI","Upper CI",
                     "p-value")
table1
}
```

This is the table 1 for expression in **tumor** using means.

```r
all.name1 = c("Age70","Sex","InvVasc.x","InvPeri.x","StStage","StT","StN","StM")
table1.t = do.table1(yt,name0="exprs.t",all.name1=all.name1,type="mean")
table1.t
```

```
##                 No. of patients Expression level    Lower CI  Upper CI
## Age70 (0,70]                 37         47.80613  25.6195394  69.99273
## Age70 (70,Inf]               56        100.56172  28.4315203 172.69191
## Sex Female                   35        105.61784   0.2312487 211.00444
## Sex Male                     58         63.85618  29.4991540  98.21321
## InvVasc.x No                 55         45.51692  30.4122848  60.62155
## InvVasc.x Yes                28         38.39837  21.7671645  55.02957
```

```
## InvPeri.x No                 60         43.92473  29.8986769  57.95079
## InvPeri.x Yes                23         41.00439  21.4225177  60.58626
## StStage 1                    22         54.33305  18.9872513  89.67884
## StStage 2                    25         35.99235  21.3547912  50.62990
## StStage 3                    33         40.81220  28.0710604  53.55334
## StStage 4                    17        262.15478  25.9558253 498.35374
## StT 1                         9         46.95883  19.9141761  74.00348
## StT 2                        19        186.94229 -11.2138507 385.09843
## StT 3                        35         67.08684  11.9061133 122.26757
## StT 4                        32         48.60192  33.3608788  63.84297
## StN 0                        51         63.93237  23.4018632 104.46288
## StN 1                        28        111.44423  -2.8186487 225.70710
## StN 2                        15         99.59143 -33.9589711 233.14184
## StM 0                        80         43.02423  31.5733129  54.47514
## StM 1                        17        262.15478  25.9558253 498.35374
##                      p-value
## Age70 (0,70]   1.00000000
## Age70 (70,Inf] 0.16556987
## Sex Female     1.00000000
## Sex Male       0.44885414
## InvVasc.x No   1.00000000
## InvVasc.x Yes  0.52220146
## InvPeri.x No   1.00000000
## InvPeri.x Yes  0.80494345
## StStage 1      1.00000000
## StStage 2      0.32777990
## StStage 3      0.46183854
## StStage 4      0.08296715
## StT 1          1.00000000
## StT 2          0.15756632
## StT 3          0.49993288
## StT 4          0.90749603
## StN 0          1.00000000
## StN 1          0.42800586
## StN 2          0.59296070
## StM 0          1.00000000
## StM 1          0.06704033
```

The analogous table using medians.

```
all.name1 = c("Age70","Sex","InvVasc.x","InvPeri.x","StStage","StT","StN","StM")
table1.t = do.table1(yt,name0="exprs.t",all.name1=all.name1,type="median")
```

```
## Warning in wilcox.test.default(x1, x0): cannot compute exact p-value with
## ties

## Warning in wilcox.test.default(x1, x0): cannot compute exact p-value with
## ties

## Warning in wilcox.test.default(x1, x0): cannot compute exact p-value with
## ties

## Warning in wilcox.test.default(x1, x0): cannot compute exact p-value with
## ties
```

```
## Warning in wilcox.test.default(x1, x0): cannot compute exact p-value with
## ties

## Warning in wilcox.test.default(x1, x0): cannot compute exact p-value with
## ties

## Warning in wilcox.test.default(x1, x0): cannot compute exact p-value with
## ties

## Warning in wilcox.test.default(x1, x0): cannot compute exact p-value with
## ties

## Warning in wilcox.test.default(x1, x0): cannot compute exact p-value with
## ties
```

```
table1.t
```

```
##                 No. of patients Expression level Lower CI  Upper CI
## Age70 (0,70]                 37        28.767179 0.110000  367.3486
## Age70 (70,Inf]               56        34.202676 0.110000 1598.2848
## Sex Female                   35        28.767179 0.110000 1598.2848
## Sex Male                     58        32.306047 0.110000  935.9264
## InvVasc.x No                 55        31.774676 0.110000  367.3486
## InvVasc.x Yes                28        24.800174 0.110000  172.3866
## InvPeri.x No                 60        28.777237 0.110000  367.3486
## InvPeri.x Yes                23        24.732727 0.110000  172.3866
## StStage 1                    22        27.449652 0.110000  367.3486
## StStage 2                    25        28.787296 0.110000  172.3866
## StStage 3                    33        32.837418 0.110000  133.8930
## StStage 4                    17        54.940000 0.110000 1598.2848
## StT 1                         9        42.696131 4.849231  118.8372
## StT 2                        19        26.936151 0.110000 1598.2848
## StT 3                        35        27.828860 0.110000  935.9264
## StT 4                        32        46.858287 0.110000  158.9786
## StN 0                        51        28.787296 0.110000  983.1652
## StN 1                        28        54.572111 0.110000 1598.2848
## StN 2                        15         9.428999 0.110000  935.9264
## StM 0                        80        28.777237 0.110000  367.3486
## StM 1                        17        54.940000 0.110000 1598.2848
##                    p-value
## Age70 (0,70]   1.00000000
## Age70 (70,Inf] 0.56918391
## Sex Female     1.00000000
## Sex Male       0.60335328
## InvVasc.x No   1.00000000
## InvVasc.x Yes  0.47582104
## InvPeri.x No   1.00000000
## InvPeri.x Yes  0.70645519
## StStage 1      1.00000000
## StStage 2      0.83940244
## StStage 3      0.91784681
## StStage 4      0.21247020
## StT 1          1.00000000
## StT 2          0.73044630
## StT 3          0.33663460
```

```
## StT 4          0.98765969
## StN 0          1.00000000
## StN 1          0.07192069
## StN 2          0.54027193
## StM 0          1.00000000
## StM 1          0.10971349
```

This is the table 1 for expression in **no tumor**.

```
all.name1 = c("Age70","Sex","InvVasc.x","InvPeri.x","StStage","StT","StN","StM")
table1.nt = do.table1(yt,name0="exprs.nt",all.name1,type="mean")
table1.nt
```

```
##                No. of patients Expression level    Lower CI   Upper CI
## Age70 (0,70]                37         9.648715   2.9615855  16.335844
## Age70 (70,Inf]              56        23.690178   5.4659954  41.914362
## Sex Female                  35        11.847165  -0.7372463  24.431577
## Sex Male                    58        21.879339   5.3321048  38.426573
## InvVasc.x No                55        20.559711   3.4432947  37.676127
## InvVasc.x Yes               28         4.147511   1.9844496   6.310573
## InvPeri.x No                60        17.721650   2.0449640  33.398337
## InvPeri.x Yes               23         7.983278   2.5639815  13.402575
## StStage 1                   22        13.556671   3.8315700  23.281773
## StStage 2                   25         7.153436   1.5817696  12.725103
## StStage 3                   33         9.640099   3.9373849  15.342813
## StStage 4                   17        55.778875  -4.9069315 116.464682
## StT 1                        9        10.501498  -0.6715954  21.674592
## StT 2                       19        15.609904   3.4719947  27.747814
## StT 3                       35        21.254248  -5.3594489  47.867946
## StT 4                       32        18.904577   3.4629393  34.346215
## StN 0                       51        19.495739   1.1000371  37.891441
## StN 1                       28        16.178824   5.4392046  26.918444
## StN 2                       15        19.357138 -10.2345722  48.948848
## StM 0                       80         9.940074   6.1233499  13.756799
## StM 1                       17        55.778875  -4.9069315 116.464682
##                   p-value
## Age70 (0,70]   1.00000000
## Age70 (70,Inf] 0.15116766
## Sex Female     1.00000000
## Sex Male       0.33386227
## InvVasc.x No   1.00000000
## InvVasc.x Yes  0.06157001
## InvPeri.x No   1.00000000
## InvPeri.x Yes  0.24229840
## StStage 1      1.00000000
## StStage 2      0.24389509
## StStage 3      0.47706851
## StStage 4      0.16386671
## StT 1          1.00000000
## StT 2          0.50438894
## StT 3          0.44570372
## StT 4          0.35588327
## StN 0          1.00000000
## StN 1          0.75408186
## StN 2          0.99338244
```

```
## StM 0          1.00000000
## StM 1          0.12950004
```

```
all.name1 = c("Age70","Sex","InvVasc.x","InvPeri.x","StStage","StT","StN","StM")
table1.nt = do.table1(yt,name0="exprs.nt",all.name1,type="median")
```

```
## Warning in wilcox.test.default(x1, x0): cannot compute exact p-value with
## ties

## Warning in wilcox.test.default(x1, x0): cannot compute exact p-value with
## ties

## Warning in wilcox.test.default(x1, x0): cannot compute exact p-value with
## ties

## Warning in wilcox.test.default(x1, x0): cannot compute exact p-value with
## ties

## Warning in wilcox.test.default(x1, x0): cannot compute exact p-value with
## ties

## Warning in wilcox.test.default(x1, x0): cannot compute exact p-value with
## ties

## Warning in wilcox.test.default(x1, x0): cannot compute exact p-value with
## ties

## Warning in wilcox.test.default(x1, x0): cannot compute exact p-value with
## ties

## Warning in wilcox.test.default(x1, x0): cannot compute exact p-value with
## ties

## Warning in wilcox.test.default(x1, x0): cannot compute exact p-value with
## ties
```

```
table1.nt
```

```
##                 No. of patients Expression level Lower CI  Upper CI
## Age70 (0,70]                 37        1.2283671     0.11  87.23629
## Age70 (70,Inf]               56        5.2732478     0.11 458.30000
## Sex Female                   35        2.3000000     0.11 211.38533
## Sex Male                     58        3.4712765     0.11 458.30000
## InvVasc.x No                 55        3.3900000     0.11 458.30000
## InvVasc.x Yes                28        0.9144834     0.11  18.50000
## InvPeri.x No                 60        2.5440956     0.11 458.30000
## InvPeri.x Yes                23        2.1693158     0.11  51.89648
## StStage 1                    22        4.9160808     0.11  87.23629
## StStage 2                    25        3.2442351     0.11  67.64516
## StStage 3                    33        1.7189667     0.11  66.23617
## StStage 4                    17        3.3900000     0.11 458.30000
## StT 1                         9        1.2283671     0.11  39.25172
## StT 2                        19        3.4853671     0.11  87.23629
## StT 3                        35        3.2442351     0.11 458.30000
## StT 4                        32        2.2346579     0.11 211.38533
## StN 0                        51        3.3556214     0.11 458.30000
```

```
## StN 1                        28        0.7925996     0.11 118.46000
## StN 2                        15        6.3000000     0.11 211.38533
## StM 0                        80        2.4787535     0.11  87.23629
## StM 1                        17        3.3900000     0.11 458.30000
##                    p-value
## Age70 (0,70]     1.00000000
## Age70 (70,Inf] 0.11108315
## Sex Female     1.00000000
## Sex Male       0.21155871
## InvVasc.x No   1.00000000
## InvVasc.x Yes  0.08612828
## InvPeri.x No   1.00000000
## InvPeri.x Yes  0.77256582
## StStage 1      1.00000000
## StStage 2      0.59256876
## StStage 3      0.49208657
## StStage 4      0.71547977
## StT 1          1.00000000
## StT 2          0.83760976
## StT 3          0.92871880
## StT 4          0.96062020
## StN 0          1.00000000
## StN 1          0.76162127
## StN 2          0.90637159
## StM 0          1.00000000
## StM 1          0.37981061
```

Marginal analysis for the fold-change

```
all.name1 = c("Age70","Sex","InvVasc.x","InvPeri.x","StStage","StT","StN","StM")
table1.fc = do.table1(yt,name0="fc",all.name1,type="mean")
table1.fc
```

```
##                No. of patients Expression level    Lower CI  Upper CI
## Age70 (0,70]                37         29.47085   12.937881  46.00382
## Age70 (70,Inf]              56        126.71011    6.371382 247.04884
## Sex Female                  35        147.79857  -27.106519 322.70366
## Sex Male                    58         51.95237   -1.886676 105.79142
## InvVasc.x No                55         24.00746   12.661240  35.35368
## InvVasc.x Yes               28         42.90727   15.241326  70.57321
## InvPeri.x No                60         26.16823   15.089195  37.24727
## InvPeri.x Yes               23         41.37912    8.545963  74.21227
## StStage 1                   22         31.70712    8.023655  55.39058
## StStage 2                   25         28.26184    3.607884  52.91579
## StStage 3                   33         33.08682   14.125034  52.04861
## StStage 4                   17        358.93995  -41.324330 759.20423
## StT 1                        9         32.00304    5.920048  58.08603
## StT 2                       19        247.12131  -81.637571 575.88019
## StT 3                       35         65.05691  -24.580329 154.69416
## StT 4                       32         41.00470   17.528742  64.48066
## StN 0                       51         59.19380   -5.056601 123.44419
## StN 1                       28        135.31893  -55.382066 326.01993
## StN 2                       15        117.56137 -100.124641 335.24737
```

```
## StM 0                       80         31.19960   19.000784  43.39841
## StM 1                       17        358.93995  -41.324330 759.20423
##                    p-value
## Age70 (0,70]   1.0000000
## Age70 (70,Inf] 0.1140981
## Sex Female     1.0000000
## Sex Male       0.2940500
## InvVasc.x No   1.0000000
## InvVasc.x Yes  0.2042566
## InvPeri.x No   1.0000000
## InvPeri.x Yes  0.3723126
## StStage 1      1.0000000
## StStage 2      0.8355837
## StStage 3      0.9256824
## StStage 4      0.1027367
## StT 1          1.0000000
## StT 2          0.1870176
## StT 3          0.4723535
## StT 4          0.5817600
## StN 0          1.0000000
## StN 1          0.4440790
## StN 2          0.5905466
## StM 0          1.0000000
## StM 1          0.1019388
```

```
all.name1 = c("Age70","Sex","InvVasc.x","InvPeri.x","StStage","StT","StN","StM")
table1.fc = do.table1(yt,name0="fc",all.name1,type="median")
```

```
## Warning in wilcox.test.default(x1, x0): cannot compute exact p-value with
## ties

## Warning in wilcox.test.default(x1, x0): cannot compute exact p-value with
## ties

## Warning in wilcox.test.default(x1, x0): cannot compute exact p-value with
## ties

## Warning in wilcox.test.default(x1, x0): cannot compute exact p-value with
## ties

## Warning in wilcox.test.default(x1, x0): cannot compute exact p-value with
## ties

## Warning in wilcox.test.default(x1, x0): cannot compute exact p-value with
## ties

## Warning in wilcox.test.default(x1, x0): cannot compute exact p-value with
## ties

## Warning in wilcox.test.default(x1, x0): cannot compute exact p-value with
## ties
```

```
table1.fc
```

```
##                  No. of patients Expression level    Lower CI   Upper CI
## Age70 (0,70]                  37         8.034515 0.76656159  227.51769
```

```
## Age70 (70,Inf]                56          3.871796 0.02706792 2620.95863
## Sex Female                    35          3.989284 0.15305993 2620.95863
## Sex Male                      58          6.997014 0.02706792 1535.12526
## InvVasc.x No                  55          5.050141 0.01134369  227.51769
## InvVasc.x Yes                 28          8.684475 0.06662699  283.42068
## InvPeri.x No                  60          5.777884 0.01134369  227.51769
## InvPeri.x Yes                 23          7.090374 0.06662699  283.42068
## StStage 1                     22          5.076087 0.01134369  227.51769
## StStage 2                     25          5.349389 0.56126509  283.42068
## StStage 3                     33          9.499602 0.06662699  220.31636
## StStage 4                     17          2.304201 0.02706792 2620.95863
## StT 1                          9          8.769232 0.48957825   79.43169
## StT 2                         19          4.192662 0.01134369 2620.95863
## StT 3                         35          3.541427 0.27833479 1535.12526
## StT 4                         32          7.562444 0.02706792  241.44943
## StN 0                         51          5.050141 0.01134369 1612.56593
## StN 1                         28         11.567051 0.02706792 2620.95863
## StN 2                         15          7.090374 0.06662699 1535.12526
## StM 0                         80          6.481704 0.01134369  283.42068
## StM 1                         17          2.304201 0.02706792 2620.95863
##                    p-value
## Age70 (0,70]     1.0000000
## Age70 (70,Inf] 0.2666444
## Sex Female       1.0000000
## Sex Male         0.8304374
## InvVasc.x No     1.0000000
## InvVasc.x Yes    0.3729289
## InvPeri.x No     1.0000000
## InvPeri.x Yes    0.7952680
## StStage 1        1.0000000
## StStage 2        0.9066430
## StStage 3        0.7246736
## StStage 4        0.9774041
## StT 1            1.0000000
## StT 2            0.7354501
## StT 3            0.3667363
## StT 4            0.7451785
## StN 0            1.0000000
## StN 1            0.2323751
## StN 2            0.6461762
## StM 0            1.0000000
## StM 1            0.7977769
```

```
all.name1 = c("Age70","Sex","InvVasc.x","InvPeri.x","StStage","StT","StN","StM")
table1.dif = do.table1(yt,name0="exprs.dif",all.name1,type="mean")
table1.dif
```

```
##                  No. of patients Expression level   Lower CI  Upper CI
## Age70 (0,70]                  37         38.15742  19.460758  56.85408
## Age70 (70,Inf]                56         76.87154   1.794001 151.94908
## Sex Female                    35         93.77068 -13.102739 200.64410
## Sex Male                      58         41.97684   5.124597  78.82909
## InvVasc.x No                  55         24.95721   6.318212  43.59620
## InvVasc.x Yes                 28         34.25086  17.181993  51.31972
## InvPeri.x No                  60         26.20308   9.062005  43.34416
```

```
## InvPeri.x Yes             23         33.02111  12.389663  53.65256
## StStage 1                 22         40.77637   9.962813  71.58993
## StStage 2                 25         28.83891  13.302361  44.37546
## StStage 3                 33         31.17210  18.413636  43.93056
## StStage 4                 17        206.37591 -48.498233 461.25005
## StT 1                      9         36.45733   4.558807  68.35585
## StT 2                     19        171.33239 -28.112486 370.77726
## StT 3                     35         45.83259 -14.276902 105.94209
## StT 4                     32         29.69735   7.884349  51.51034
## StN 0                     51         44.43663   1.447281  87.42598
## StN 1                     28         95.26540 -20.815976 211.34678
## StN 2                     15         80.23430 -58.817024 219.28562
## StM 0                     80         33.08415  22.499104  43.66920
## StM 1                     17        206.37591 -48.498233 461.25005
##                    p-value
## Age70 (0,70]    1.0000000
## Age70 (70,Inf] 0.3195695
## Sex Female      1.0000000
## Sex Male        0.3578315
## InvVasc.x No    1.0000000
## InvVasc.x Yes   0.4585709
## InvPeri.x No    1.0000000
## InvPeri.x Yes   0.6055914
## StStage 1       1.0000000
## StStage 2       0.4778953
## StStage 3       0.5551817
## StStage 4       0.1899690
## StT 1           1.0000000
## StT 2           0.1761045
## StT 3           0.7754328
## StT 4           0.7034088
## StN 0           1.0000000
## StN 1           0.4064529
## StN 2           0.6067594
## StM 0           1.0000000
## StM 1           0.1690903
```

```
all.name1 = c("Age70","Sex","InvVasc.x","InvPeri.x","StStage","StT","StN","StM")
table1.dif = do.table1(yt,name0="exprs.dif",all.name1,type="median")
```

```
## Warning in wilcox.test.default(x1, x0): cannot compute exact p-value with
## ties

## Warning in wilcox.test.default(x1, x0): cannot compute exact p-value with
## ties

## Warning in wilcox.test.default(x1, x0): cannot compute exact p-value with
## ties

## Warning in wilcox.test.default(x1, x0): cannot compute exact p-value with
## ties

## Warning in wilcox.test.default(x1, x0): cannot compute exact p-value with
## ties
```

```
## Warning in wilcox.test.default(x1, x0): cannot compute exact p-value with
## ties

## Warning in wilcox.test.default(x1, x0): cannot compute exact p-value with
## ties

## Warning in wilcox.test.default(x1, x0): cannot compute exact p-value with
## ties
```

```
table1.dif
```

```
##                 No. of patients Expression level    Lower CI  Upper CI
## Age70 (0,70]                 37        21.388617   -6.413196  280.1123
## Age70 (70,Inf]               56        20.202695 -331.100000 1598.1748
## Sex Female                   35        19.990579 -154.702412 1598.1748
## Sex Male                     58        20.901714 -331.100000  935.8164
## InvVasc.x No                 55        20.558688 -331.100000  280.1123
## InvVasc.x Yes                28        24.690174   -9.156243  172.2766
## InvPeri.x No                 60        23.073119 -331.100000  280.1123
## InvPeri.x Yes                23        18.477231  -10.246145  172.2766
## StStage 1                    22        24.205920  -53.164384  280.1123
## StStage 2                    25        25.688914  -29.897660  172.2766
## StStage 3                    33        24.622727  -10.246145  133.7830
## StStage 4                    17         6.207845 -331.100000 1598.1748
## StT 1                         9        42.586131  -20.290145  117.6089
## StT 2                        19        20.558688  -53.164384 1598.1748
## StT 3                        35        15.888073 -331.100000  935.8164
## StT 4                        32        26.717459 -154.702412  146.6741
## StN 0                        51        20.558688 -331.100000  983.0552
## StN 1                        28        31.862752 -115.740000 1598.1748
## StN 2                        15         5.184757 -154.702412  935.8164
## StM 0                        80        24.690174  -53.164384  280.1123
## StM 1                        17         6.207845 -331.100000 1598.1748
##                   p-value
## Age70 (0,70]    1.0000000
## Age70 (70,Inf]  0.5533814
## Sex Female      1.0000000
## Sex Male        0.9367850
## InvVasc.x No    1.0000000
## InvVasc.x Yes   0.8510079
## InvPeri.x No    1.0000000
## InvPeri.x Yes   0.6288560
## StStage 1       1.0000000
## StStage 2       0.8561780
## StStage 3       0.9520481
## StStage 4       0.9548264
## StT 1           1.0000000
## StT 2           1.0000000
## StT 3           0.4317863
## StT 4           0.7925186
## StN 0           1.0000000
## StN 1           0.2055055
## StN 2           0.3585343
## StM 0           1.0000000
## StM 1           0.8272219
```

Linear models with difference of expressions as response

```
df = data.frame(exprs.dif,StT = yt$StT,StN=yt$StN,StStage = yt$StStage,
                StM = yt$StM)
df = na.omit(df)
```

```
fitTNMS.lm = lm(exprs.dif ~ StT + StN + StM + StStage ,data=df)
summary(fitTNMS.lm)
```

```
##
## Call:
## lm(formula = exprs.dif ~ StT + StN + StM + StStage, data = df)
##
## Residuals:
##     Min      1Q  Median      3Q     Max
## -416.27  -39.06   -1.61   39.70  924.69
##
## Coefficients: (1 not defined because of singularities)
##             Estimate Std. Error t value Pr(>|t|)
## (Intercept)   36.457     63.031   0.578 0.564523
## StT2           7.309     81.997   0.089 0.929181
## StT3        -412.727    124.757  -3.308 0.001378 **
## StT4        -447.820    123.712  -3.620 0.000500 ***
## StN1         301.412    121.100   2.489 0.014762 *
## StN2         325.991    125.905   2.589 0.011316 *
## StM1         328.305    117.898   2.785 0.006604 **
## StStage2     416.338    111.329   3.740 0.000333 ***
## StStage3      77.730    137.513   0.565 0.573389
## StStage4          NA         NA      NA       NA
## ---
## Signif. codes:  0 '***' 0.001 '**' 0.01 '*' 0.05 '.' 0.1 ' ' 1
##
## Residual standard error: 189.1 on 85 degrees of freedom
## Multiple R-squared:  0.3278, Adjusted R-squared:  0.2645
## F-statistic: 5.181 on 8 and 85 DF,  p-value: 2.79e-05
```

Can we remove some of the predictors? We apply a stepwise variable selection minimizing the Akaike information criterium.

```
fitTNMS.step = stepAIC(fitTNMS.lm)
```

```
## Start:  AIC=994.08
## exprs.dif ~ StT + StN + StM + StStage
##
##
## Step:  AIC=994.08
## exprs.dif ~ StT + StN + StStage
##
##           Df Sum of Sq     RSS     AIC
## <none>                 3039306  994.08
## - StN      2    248187 3287493  997.46
## - StT      3    872426 3911732 1011.80
## - StStage  3   1038410 4077716 1015.71
```

```r
summary(fitTNMS.step)
```

```
##
## Call:
## lm(formula = exprs.dif ~ StT + StN + StStage, data = df)
##
## Residuals:
##     Min      1Q  Median      3Q     Max
## -416.27  -39.06   -1.61   39.70  924.69
##
## Coefficients:
##             Estimate Std. Error t value Pr(>|t|)
## (Intercept)   36.457     63.031   0.578 0.564523
## StT2           7.309     81.997   0.089 0.929181
## StT3        -412.727    124.757  -3.308 0.001378 **
## StT4        -447.820    123.712  -3.620 0.000500 ***
## StN1         301.412    121.100   2.489 0.014762 *
## StN2         325.991    125.905   2.589 0.011316 *
## StStage2     416.338    111.329   3.740 0.000333 ***
## StStage3      77.730    137.513   0.565 0.573389
## StStage4     328.305    117.898   2.785 0.006604 **
## ---
## Signif. codes:  0 '***' 0.001 '**' 0.01 '*' 0.05 '.' 0.1 ' ' 1
##
## Residual standard error: 189.1 on 85 degrees of freedom
## Multiple R-squared:  0.3278, Adjusted R-squared:  0.2645
## F-statistic: 5.181 on 8 and 85 DF,  p-value: 2.79e-05
```

Note that all predictors remains in the model except StM.

We are goint to fit the model removing each predictor.

```r
fitNMS.lm = lm(exprs.dif ~  StN + StM + StStage ,data=df)
fitTMS.lm = lm(exprs.dif ~ StT +  StM + StStage ,data=df)
fitTNS.lm = lm(exprs.dif ~ StT + StN +  StStage ,data=df)
fitTNM.lm = lm(exprs.dif ~ StT + StN + StM ,data=df)
```

We can evaluate the effect of removing one variable from the model with the four predictors.

Removing StT.

```r
anova(fitTNMS.lm,fitNMS.lm)
```

```
## Analysis of Variance Table
##
## Model 1: exprs.dif ~ StT + StN + StM + StStage
## Model 2: exprs.dif ~ StN + StM + StStage
##   Res.Df     RSS Df Sum of Sq     F    Pr(>F)
## 1     85 3039306
## 2     88 3911732 -3   -872426 8.133 8.004e-05 ***
## ---
## Signif. codes:  0 '***' 0.001 '**' 0.01 '*' 0.05 '.' 0.1 ' ' 1
```

Removing StN.

```r
anova(fitTNMS.lm,fitTMS.lm)
```

```
## Analysis of Variance Table
```

```
##
## Model 1: exprs.dif ~ StT + StN + StM + StStage
## Model 2: exprs.dif ~ StT + StM + StStage
##   Res.Df     RSS Df Sum of Sq      F  Pr(>F)
## 1     85 3039306
## 2     87 3287493 -2   -248187 3.4705 0.03558 *
## ---
## Signif. codes:  0 '***' 0.001 '**' 0.01 '*' 0.05 '.' 0.1 ' ' 1
```

Removing StM.

```
anova(fitTNMS.lm,fitTNS.lm)
```

```
## Analysis of Variance Table
##
## Model 1: exprs.dif ~ StT + StN + StM + StStage
## Model 2: exprs.dif ~ StT + StN + StStage
##   Res.Df     RSS Df Sum of Sq F Pr(>F)
## 1     85 3039306
## 2     85 3039306  0         0
```

Removing StStage.

```
anova(fitTNMS.lm,fitTNM.lm)
```

```
## Analysis of Variance Table
##
## Model 1: exprs.dif ~ StT + StN + StM + StStage
## Model 2: exprs.dif ~ StT + StN + StM
##   Res.Df     RSS Df Sum of Sq      F    Pr(>F)
## 1     85 3039306
## 2     87 3603645 -2   -564339 7.8914 0.0007184 ***
## ---
## Signif. codes:  0 '***' 0.001 '**' 0.01 '*' 0.05 '.' 0.1 ' ' 1
```

From now on, we use the model without `StM` according with the previous selection.

```
fitTNS.lm = lm(exprs.dif ~  StT + StN + StStage,data=df)
summary(fitTNS.lm)
```

```
##
## Call:
## lm(formula = exprs.dif ~ StT + StN + StStage, data = df)
##
## Residuals:
##     Min      1Q  Median      3Q     Max
## -416.27  -39.06   -1.61   39.70  924.69
##
## Coefficients:
##             Estimate Std. Error t value Pr(>|t|)
## (Intercept)   36.457     63.031   0.578 0.564523
## StT2           7.309     81.997   0.089 0.929181
## StT3        -412.727    124.757  -3.308 0.001378 **
## StT4        -447.820    123.712  -3.620 0.000500 ***
## StN1         301.412    121.100   2.489 0.014762 *
## StN2         325.991    125.905   2.589 0.011316 *
## StStage2     416.338    111.329   3.740 0.000333 ***
## StStage3      77.730    137.513   0.565 0.573389
```

```
## StStage4     328.305    117.898   2.785 0.006604 **
## ---
## Signif. codes:  0 '***' 0.001 '**' 0.01 '*' 0.05 '.' 0.1 ' ' 1
##
## Residual standard error: 189.1 on 85 degrees of freedom
## Multiple R-squared:  0.3278, Adjusted R-squared:  0.2645
## F-statistic: 5.181 on 8 and 85 DF,  p-value: 2.79e-05
```

Now we are going to calculate the p-value to remove each single categorical predictor.

```
fitNS.lm = lm(exprs.dif ~  StN + StStage ,data=df)
fitTS.lm = lm(exprs.dif ~ StT  + StStage,data=df)
fitTN.lm = lm(exprs.dif ~ StT  + StN,data=df)
```

We can compare the models with and without the categorical predictors.

```
anova(fitTNS.lm,fitNS.lm)
```

```
## Analysis of Variance Table
##
## Model 1: exprs.dif ~ StT + StN + StStage
## Model 2: exprs.dif ~ StN + StStage
##   Res.Df     RSS Df Sum of Sq     F    Pr(>F)
## 1     85 3039306
## 2     88 3911732 -3   -872426 8.133 8.004e-05 ***
## ---
## Signif. codes:  0 '***' 0.001 '**' 0.01 '*' 0.05 '.' 0.1 ' ' 1
```

```
anova(fitTNS.lm,fitTS.lm)
```

```
## Analysis of Variance Table
##
## Model 1: exprs.dif ~ StT + StN + StStage
## Model 2: exprs.dif ~ StT + StStage
##   Res.Df     RSS Df Sum of Sq      F  Pr(>F)
## 1     85 3039306
## 2     87 3287493 -2   -248187 3.4705 0.03558 *
## ---
## Signif. codes:  0 '***' 0.001 '**' 0.01 '*' 0.05 '.' 0.1 ' ' 1
```

```
anova(fitTNS.lm,fitTN.lm)
```

```
## Analysis of Variance Table
##
## Model 1: exprs.dif ~ StT + StN + StStage
## Model 2: exprs.dif ~ StT + StN
##   Res.Df     RSS Df Sum of Sq      F    Pr(>F)
## 1     85 3039306
## 2     88 4077716 -3  -1038410 9.6804 1.454e-05 ***
## ---
## Signif. codes:  0 '***' 0.001 '**' 0.01 '*' 0.05 '.' 0.1 ' ' 1
```

Note that the three test are significant. The minimum p-value correspond to the stage, then the StT and finally StN.

Linear models with the fold change as response

We calculate the ratio between the expressions using fc or fold-change.

```
summary(fc)
```

```
##      Min.   1st Qu.    Median      Mean   3rd Qu.      Max.
##    0.0113    1.0000    5.9595   88.6386   41.3651 2620.9586
```

```
df = data.frame(fc,StT = yt$StT,StN=yt$StN,StStage = yt$StStage,StM = yt$StM)
df = na.omit(df)
fitTNMS.fc = lm(fc ~ StT + StN +StM + StStage ,data=df)
```

Let us see a summary of both fits.

```
summary(fitTNMS.fc)
```

```
##
## Call:
## lm(formula = fc ~ StT + StN + StM + StStage, data = df)
##
## Residuals:
##     Min      1Q  Median      3Q     Max
## -715.36  -36.81  -19.80   49.77 1601.36
##
## Coefficients: (1 not defined because of singularities)
##              Estimate Std. Error t value Pr(>|t|)
## (Intercept)   32.0030    99.4524   0.322 0.748400
## StT2          -0.5008   129.3763  -0.004 0.996921
## StT3        -625.7107   196.8444  -3.179 0.002064 **
## StT4        -670.9964   195.1953  -3.438 0.000911 ***
## StN1         303.2946   191.0747   1.587 0.116156
## StN2         344.0797   198.6566   1.732 0.086895 .
## StM1         684.7980   186.0217   3.681 0.000407 ***
## StStage2     636.4609   175.6581   3.623 0.000494 ***
## StStage3     276.3966   216.9717   1.274 0.206177
## StStage4           NA         NA      NA       NA
## ---
## Signif. codes:  0 '***' 0.001 '**' 0.01 '*' 0.05 '.' 0.1 ' ' 1
##
## Residual standard error: 298.4 on 85 degrees of freedom
## Multiple R-squared:  0.3375, Adjusted R-squared:  0.2751
## F-statistic: 5.412 on 8 and 85 DF,  p-value: 1.633e-05
```

```
aggregate(df$fc,by = list(df$StStage),FUN=var)
```

```
##   Group.1          x
## 1       1   2853.302
## 2       2   3567.274
## 3       3   2859.690
## 4       4 711550.979
```

```
aggregate(df$fc,by = list(df$StStage),FUN=IQR)
```

```
##   Group.1          x
## 1       1  62.224610
## 2       2   9.542797
## 3       3  35.063306
```

```
## 4       4 190.173101
```

Clearly, we have to use the first fit corresponding to fc. Can we remove some of the predictors?

```
fitTNMS.step = stepAIC(fitTNMS.fc)
```

```
## Start:  AIC=1079.82
## fc ~ StT + StN + StM + StStage
##
##
## Step:  AIC=1079.82
## fc ~ StT + StN + StStage
##
##           Df Sum of Sq      RSS    AIC
## - StN      2    269602  7836042 1079.1
## <none>                  7566440 1079.8
## - StT      3   1900434  9466874 1094.9
## - StStage  3   2896392 10462832 1104.3
##
## Step:  AIC=1079.11
## fc ~ StT + StStage
##
##           Df Sum of Sq      RSS    AIC
## <none>                  7836042 1079.1
## - StT      3   1651165  9487207 1091.1
## - StStage  3   2992628 10828669 1103.5
```

```
summary(fitTNMS.step)
```

```
##
## Call:
## lm(formula = fc ~ StT + StStage, data = df)
##
## Residuals:
##     Min      1Q  Median      3Q     Max
## -878.52  -32.22   -2.27   39.70 1741.50
##
## Coefficients:
##             Estimate Std. Error t value Pr(>|t|)
## (Intercept)  32.0030   100.0386   0.320  0.74981
## StT2         -0.5008   130.1389  -0.004  0.99694
## StT3       -550.8278   190.9154  -2.885  0.00493 **
## StT4       -581.4092   189.1816  -3.073  0.00283 **
## StStage2    556.8726   169.0302   3.295  0.00143 **
## StStage3    517.6359   155.8801   3.321  0.00131 **
## StStage4    847.9515   155.8801   5.440 4.84e-07 ***
## ---
## Signif. codes:  0 '***' 0.001 '**' 0.01 '*' 0.05 '.' 0.1 ' ' 1
##
## Residual standard error: 300.1 on 87 degrees of freedom
## Multiple R-squared:  0.3138, Adjusted R-squared:  0.2665
## F-statistic: 6.632 on 6 and 87 DF,  p-value: 8.428e-06
```

The variables StN and STM has been removed from the model. Now we are going to calculate the p-value to remove each single categorical predictor.

```r
fitT.ft = lm(fc ~ StN + StM + StStage ,data=df)
fitN.ft = lm(fc ~ StT + StM + StStage,data=df)
fitM.ft = lm(fc ~ StT + StN + StStage,data=df)
fitStage.ft = lm(fc ~ StT + StN + StM,data=df)
```

We can compare the models with and without the categorical predictors.

```r
anova(fitTNMS.fc,fitT.ft)
```

```
## Analysis of Variance Table
##
## Model 1: fc ~ StT + StN + StM + StStage
## Model 2: fc ~ StN + StM + StStage
##   Res.Df     RSS Df Sum of Sq      F    Pr(>F)
## 1     85 7566440
## 2     88 9466874 -3  -1900434 7.1164 0.0002538 ***
## ---
## Signif. codes:  0 '***' 0.001 '**' 0.01 '*' 0.05 '.' 0.1 ' ' 1
```

```r
anova(fitTNMS.fc,fitN.ft)
```

```
## Analysis of Variance Table
##
## Model 1: fc ~ StT + StN + StM + StStage
## Model 2: fc ~ StT + StM + StStage
##   Res.Df     RSS Df Sum of Sq      F Pr(>F)
## 1     85 7566440
## 2     87 7836042 -2   -269602 1.5143 0.2258
```

```r
anova(fitTNMS.fc,fitM.ft)
```

```
## Analysis of Variance Table
##
## Model 1: fc ~ StT + StN + StM + StStage
## Model 2: fc ~ StT + StN + StStage
##   Res.Df     RSS Df Sum of Sq F Pr(>F)
## 1     85 7566440
## 2     85 7566440  0         0
```

```r
anova(fitTNMS.fc,fitStage.ft)
```

```
## Analysis of Variance Table
##
## Model 1: fc ~ StT + StN + StM + StStage
## Model 2: fc ~ StT + StN + StM
##   Res.Df     RSS Df Sum of Sq     F   Pr(>F)
## 1     85 7566440
## 2     87 8755350 -2  -1188910 6.678 0.002024 **
## ---
## Signif. codes:  0 '***' 0.001 '**' 0.01 '*' 0.05 '.' 0.1 ' ' 1
```

```r
df1 = data.frame(fc,StT = yt$StT)
fitT.ft = lm(fc ~ StT ,data=df1)
summary(fitT.ft)
```

```
##
## Call:
```

```
## lm(formula = fc ~ StT, data = df1)
##
## Residuals:
##     Min      1Q  Median      3Q     Max
## -247.11  -64.06  -40.48  -21.42 2373.84
##
## Coefficients:
##             Estimate Std. Error t value Pr(>|t|)
## (Intercept)   32.003    114.995   0.278    0.781
## StT2         215.118    139.598   1.541    0.127
## StT3          33.054    128.935   0.256    0.798
## StT4           9.002    130.165   0.069    0.945
##
## Residual standard error: 345 on 91 degrees of freedom
##   (2 observations deleted due to missingness)
## Multiple R-squared:  0.05232,    Adjusted R-squared:  0.02108
## F-statistic: 1.675 on 3 and 91 DF,  p-value: 0.178
```

```
summary(aov(fc ~yt$StT))
```

```
##             Df   Sum Sq Mean Sq F value Pr(>F)
## yt$StT       3   597915  199305   1.675  0.178
## Residuals   91 10830302  119014
## 2 observations deleted due to missingness
```

```
summary(aov(fc ~yt$StN))
```

```
##             Df   Sum Sq Mean Sq F value Pr(>F)
## yt$StN       2   117169   58585   0.472  0.625
## Residuals   91 11303043  124209
## 3 observations deleted due to missingness
```

```
summary(aov(fc ~yt$StM))
```

```
##             Df  Sum Sq Mean Sq F value  Pr(>F)
## yt$StM       1 1506007 1506007    14.4 0.00026 ***
## Residuals   95 9934210  104571
## ---
## Signif. codes:  0 '***' 0.001 '**' 0.01 '*' 0.05 '.' 0.1 ' ' 1
```

```
summary(aov(fc ~yt$StStage))
```

```
##             Df  Sum Sq Mean Sq F value  Pr(>F)
## yt$StStage   3 1506346  502115   4.701 0.00422 **
## Residuals   93 9933871  106816
## ---
## Signif. codes:  0 '***' 0.001 '**' 0.01 '*' 0.05 '.' 0.1 ' ' 1
```

Survival analysis for Incliva data

Calculating the survival time

The first time to event to be evaluated is the time from the beginning of the treatment to the death.

The beginning of the treatment is measured as the time of surgery.

The important variables are DoSurg, DoD, DoLF, LFState. We calculate the number of days between the time of surgery and the time of death. The analogous difference is calculated from the time of the surgery and the time to the last follow up. The survival time will be calculated as the maximum of the just calculated numbers of days.

```
t1 = yt$DoLF - yt$DoSurg
t2 = yt$DoD  - yt$DoSurg
tt = apply(cbind(t1,t2),1,max,na.rm=TRUE)
```

```
## Warning in FUN(newX[, i], ...): ningun argumento finito para max;
## retornando -Inf

## Warning in FUN(newX[, i], ...): ningun argumento finito para max;
## retornando -Inf

## Warning in FUN(newX[, i], ...): ningun argumento finito para max;
## retornando -Inf

## Warning in FUN(newX[, i], ...): ningun argumento finito para max;
## retornando -Inf
```

```
tt[tt == "-Inf"] = NA
```

Now we create a data.frame with the relevant variables.

```
ytt = data.frame(yt,tt,censored=(yt$LFState == "Dead")*1)
```

Global survival without covariables

```
surv0 = Surv(ytt$tt,ytt$censored)
fit = survfit(surv0~1,data=ytt)
```

First the Kaplan-Meir estimate of the survival function.

```
ggsurvplot(fit)
```

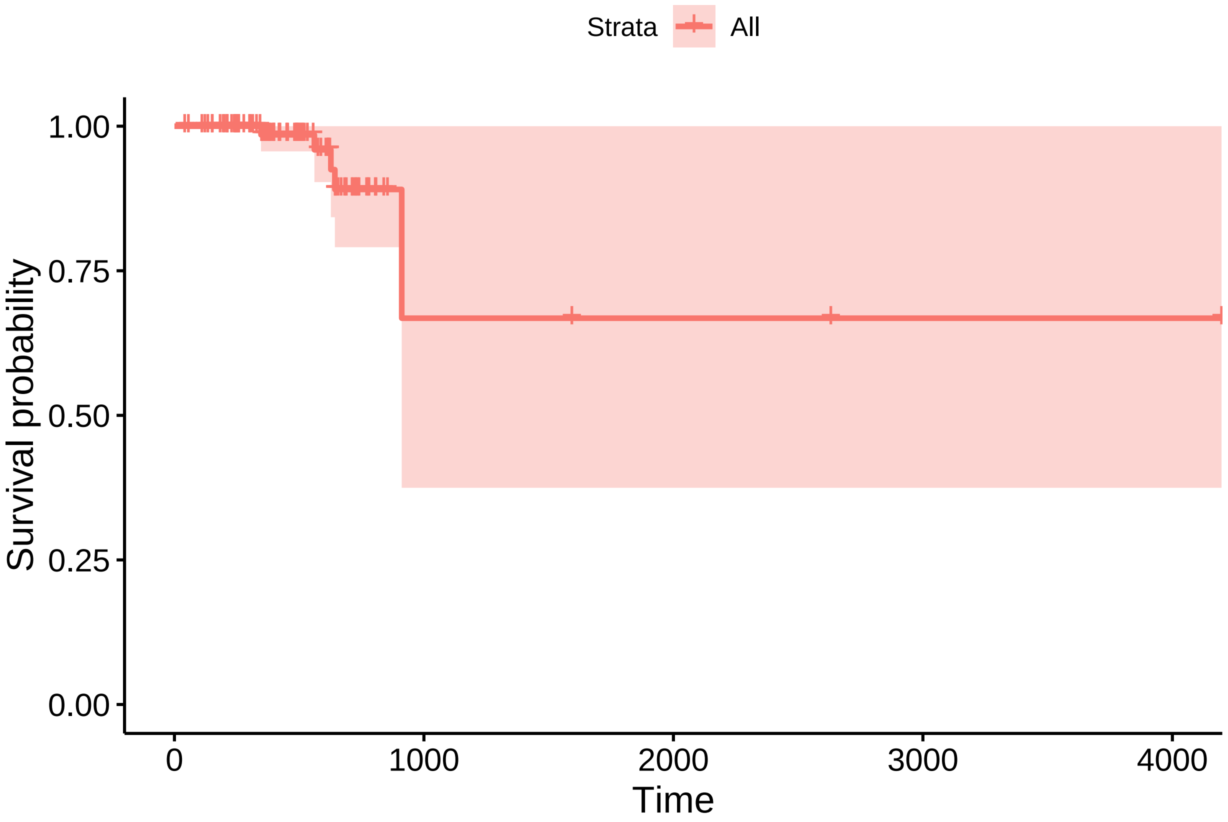

Global survival with exprs.t, exprs.nt, StT, StN, StStage

Is there dependence of the survival time with respect to the gene expresssion? We use a Cox regression.

```
fit1 = coxph(surv0 ~ exprs.t + exprs.nt + StT + StN + StM + StStage,data=ytt,
            iter=1000)
```

```
## Warning in fitter(X, Y, strats, offset, init, control, weights = weights, :
## Loglik converged before variable 7 ; beta may be infinite.
```

There is problems with the convergence in order to obtain the estimators. A summary of the fit is shown.

```
summary(fit1)
```

```
## Call:
## coxph(formula = surv0 ~ exprs.t + exprs.nt + StT + StN + StM +
##     StStage, data = ytt, iter = 1000)
##
##   n= 92, number of events= 5
##    (5 observations deleted due to missingness)
##
##                coef  exp(coef)   se(coef)      z Pr(>|z|)
## exprs.t   4.496e-03  1.005e+00  5.709e-03  0.788    0.431
## exprs.nt  2.803e-02  1.028e+00  2.246e-02  1.248    0.212
## StT2     -5.868e-02  9.430e-01  5.615e+00 -0.010    0.992
## StT3      7.526e-01  2.122e+00  1.429e+00  0.527    0.599
## StT4             NA         NA  0.000e+00     NA       NA
## StN1      7.390e-01  2.094e+00  4.723e+00  0.156    0.876
## StN2     -2.270e+01  1.386e-10  1.786e+04 -0.001    0.999
```

```
## StM1      2.023e+01  6.116e+08  2.084e+00  9.707  < 2e-16 ***
## StStage2  2.303e+01  1.004e+10  4.982e+00  4.622  3.8e-06 ***
## StStage3         NA         NA  0.000e+00     NA       NA
## StStage4         NA         NA  0.000e+00     NA       NA
## ---
## Signif. codes:  0 '***' 0.001 '**' 0.01 '*' 0.05 '.' 0.1 ' ' 1
##
##          exp(coef) exp(-coef) lower .95 upper .95
## exprs.t  1.005e+00  9.955e-01 9.933e-01 1.016e+00
## exprs.nt 1.028e+00  9.724e-01 9.841e-01 1.075e+00
## StT2     9.430e-01  1.060e+00 1.566e-05 5.680e+04
## StT3     2.122e+00  4.712e-01 1.289e-01 3.495e+01
## StT4            NA         NA        NA        NA
## StN1     2.094e+00  4.776e-01 2.000e-04 2.192e+04
## StN2     1.386e-10  7.214e+09 0.000e+00       Inf
## StM1     6.116e+08  1.635e-09 1.029e+07 3.635e+10
## StStage2 1.004e+10  9.965e-11 5.761e+05 1.748e+14
## StStage3        NA         NA        NA        NA
## StStage4        NA         NA        NA        NA
##
## Concordance= 0.873  (se = 0.156 )
## Rsquare= 0.149   (max possible= 0.291 )
## Likelihood ratio test= 14.83  on 8 df,   p=0.06
## Wald test            = 296.9  on 8 df,   p=<2e-16
## Score (logrank) test = 29.52  on 8 df,   p=3e-04
```

Global survival with exprs.t, exprs.nt, StStage

Now, we are going to evaluate the joint behaviour of exprs.t and exprs.nt with StStage.

```
fit2 = coxph(surv0 ~ exprs.t + exprs.nt + StStage,data=ytt,iter=1000)
```

```
## Warning in fitter(X, Y, strats, offset, init, control, weights = weights, :
## Loglik converged before variable 3,4,5 ; beta may be infinite.
```

A summary of the fit shows that there is no clear dependence of the Stage when the expression in tumour and control tissue is considered.

```
summary(fit2)
```

```
## Call:
## coxph(formula = surv0 ~ exprs.t + exprs.nt + StStage, data = ytt,
##     iter = 1000)
##
##   n= 93, number of events= 5
##    (4 observations deleted due to missingness)
##
##               coef exp(coef)  se(coef)     z Pr(>|z|)
## exprs.t  3.144e-03 1.003e+00 2.001e-03 1.571   0.1161
## exprs.nt 1.474e-02 1.015e+00 6.968e-03 2.115   0.0344 *
## StStage2 1.875e+01 1.391e+08 1.429e+04 0.001   0.9990
## StStage3 1.781e+01 5.453e+07 1.429e+04 0.001   0.9990
## StStage4 1.676e+01 1.905e+07 1.429e+04 0.001   0.9991
## ---
## Signif. codes:  0 '***' 0.001 '**' 0.01 '*' 0.05 '.' 0.1 ' ' 1
```

```
##
##            exp(coef) exp(-coef) lower .95 upper .95
## exprs.t    1.003e+00  9.969e-01    0.9992     1.007
## exprs.nt   1.015e+00  9.854e-01    1.0011     1.029
## StStage2   1.391e+08  7.190e-09    0.0000       Inf
## StStage3   5.453e+07  1.834e-08    0.0000       Inf
## StStage4   1.905e+07  5.250e-08    0.0000       Inf
##
## Concordance= 0.811  (se = 0.157 )
## Rsquare= 0.094   (max possible= 0.289 )
## Likelihood ratio test= 9.15  on 5 df,   p=0.1
## Wald test            = 1.06  on 5 df,   p=1
## Score (logrank) test = 15.91  on 5 df,   p=0.007
```

The comparison of the survival time using `StT`, `StN`, `StM`, `StStage` can be performed using the log-rank test. There is no significant difference between the survival functions.

```
fit3 = survfit(surv0~StT,data=ytt)
ggsurvplot(fit3,risk.table=TRUE,pval=TRUE)
```

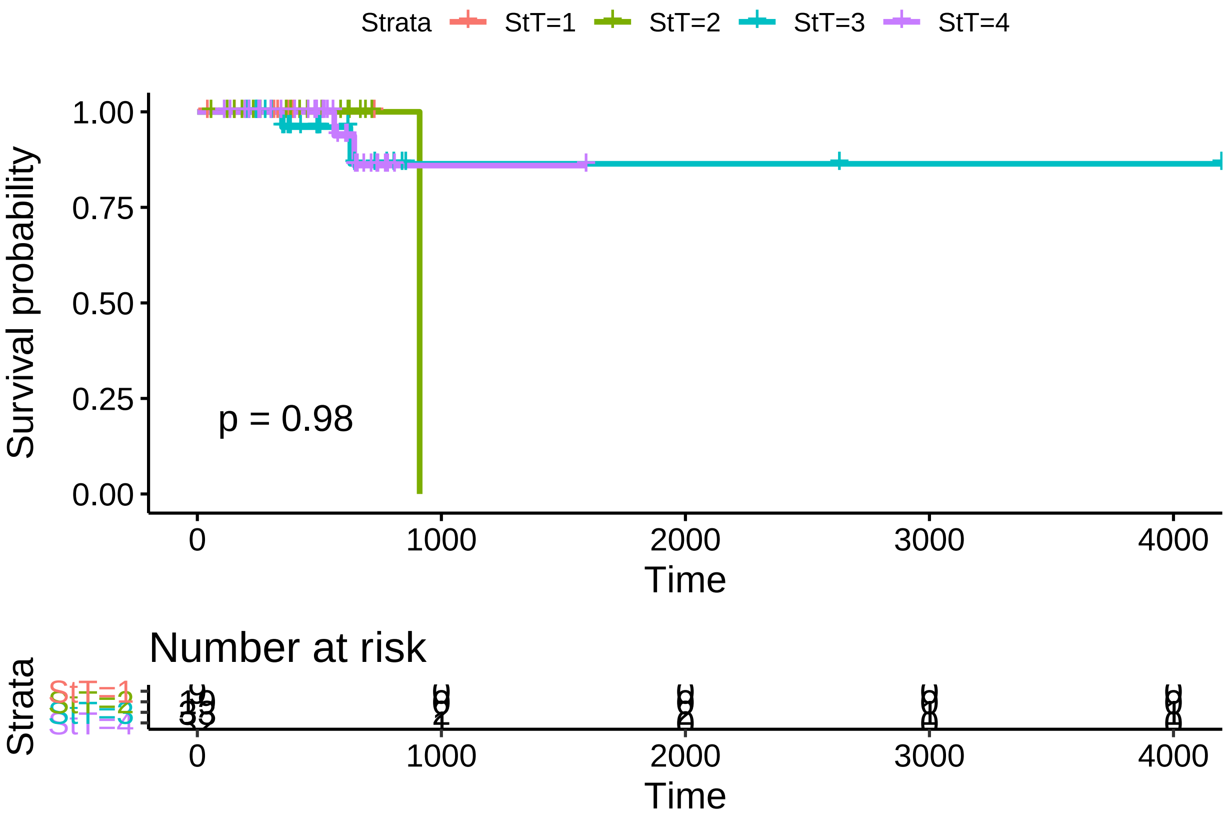


```
fit3 = survfit(surv0~StN,data=ytt)
ggsurvplot(fit3,risk.table=TRUE,pval=TRUE)
```

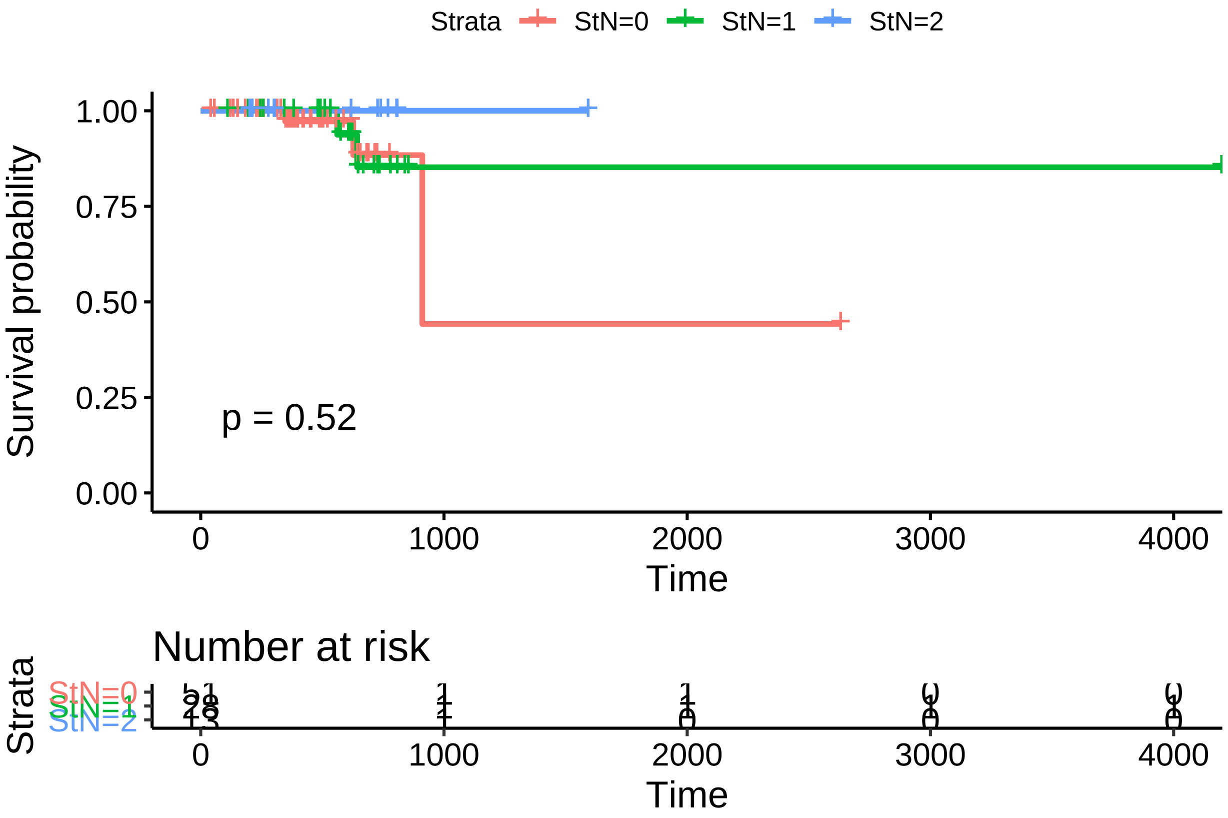


```
fit3 = survfit(surv0~StM,data=ytt)
ggsurvplot(fit3,risk.table=TRUE,pval=TRUE)
```

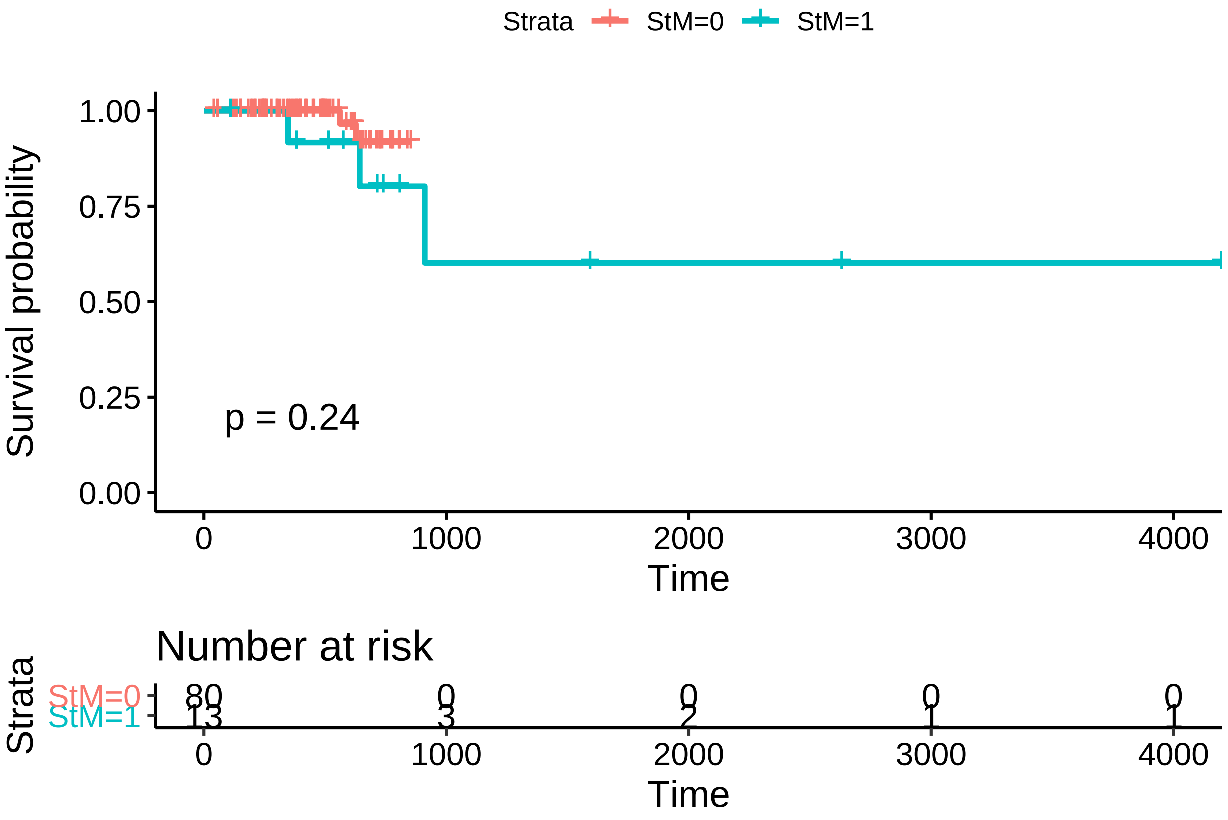

```r
fit3 = survfit(surv0~StStage,data=ytt)
ggsurvplot(fit3,risk.table=TRUE,pval=TRUE)
```

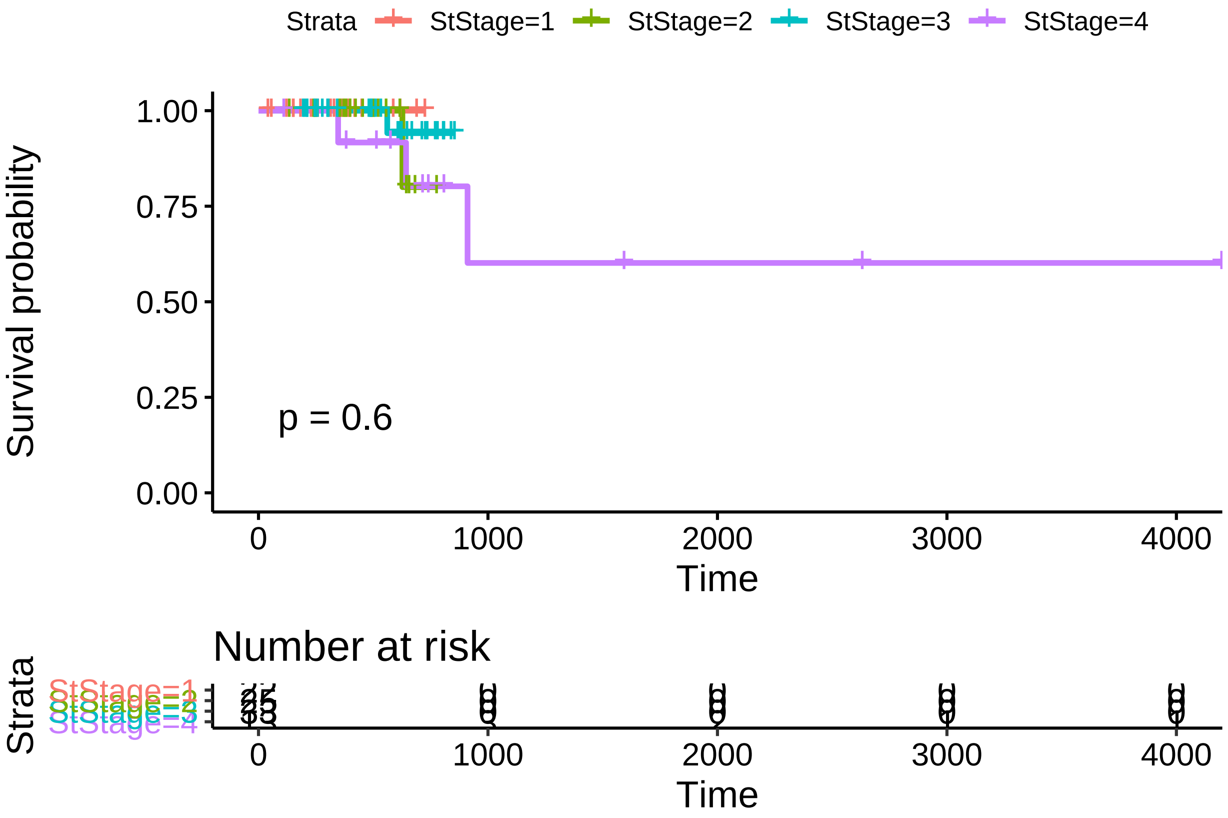


```r
fit3 = survfit(surv0~StStage,data=ytt)
ggsurvplot(fit3,risk.table=TRUE,pval=TRUE)
```

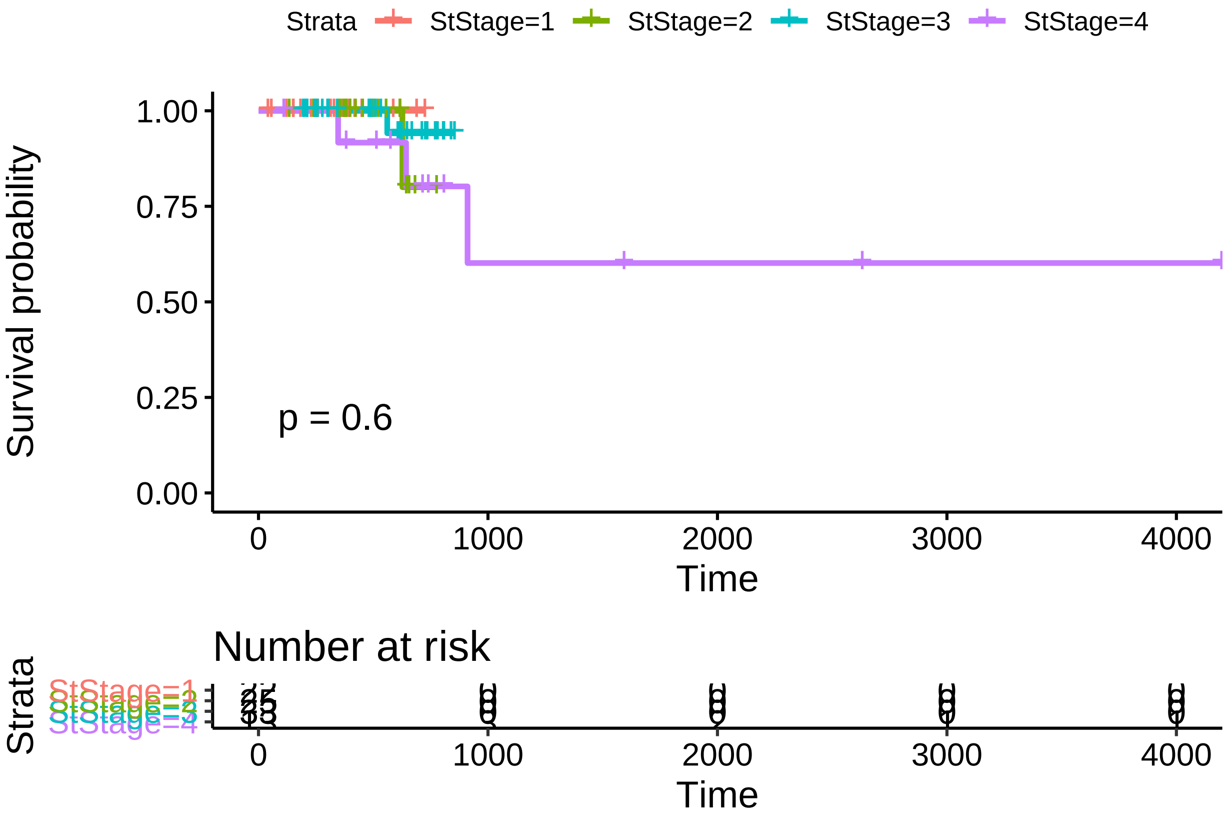

Global survival with `exprs.t`, `exprs.nt`, `StT`

Now, we are going to evaluate the joint behaviour of `exprs.t` and `exprs.nt` with `StStage`.

```
fit2 = coxph(surv0 ~ exprs.t + exprs.nt + StT,data=ytt,iter=1000)
```

```
## Warning in fitter(X, Y, strats, offset, init, control, weights = weights, :
## Loglik converged before variable 3,4,5 ; beta may be infinite.
```

A summary of the fit shows that there is no clear dependence of the Stage **given** the expression.

```
summary(fit2)
```

```
## Call:
## coxph(formula = surv0 ~ exprs.t + exprs.nt + StT, data = ytt,
##     iter = 1000)
##
##   n= 93, number of events= 5
##    (4 observations deleted due to missingness)
##
##               coef exp(coef)  se(coef)     z Pr(>|z|)
## exprs.t  3.525e-03 1.004e+00 2.867e-03 1.230   0.2188
## exprs.nt 1.086e-02 1.011e+00 4.356e-03 2.493   0.0127 *
## StT2     1.580e+01 7.264e+06 1.981e+04 0.001   0.9994
## StT3     1.757e+01 4.262e+07 1.981e+04 0.001   0.9993
## StT4     1.710e+01 2.677e+07 1.981e+04 0.001   0.9993
## ---
## Signif. codes:  0 '***' 0.001 '**' 0.01 '*' 0.05 '.' 0.1 ' ' 1
##
```

```
##          exp(coef) exp(-coef) lower .95 upper .95
## exprs.t  1.004e+00  9.965e-01    0.9979     1.009
## exprs.nt 1.011e+00  9.892e-01    1.0023     1.020
## StT2     7.264e+06  1.377e-07    0.0000       Inf
## StT3     4.262e+07  2.346e-08    0.0000       Inf
## StT4     2.677e+07  3.736e-08    0.0000       Inf
##
## Concordance= 0.786  (se = 0.157 )
## Rsquare= 0.085   (max possible= 0.289 )
## Likelihood ratio test= 8.23  on 5 df,   p=0.1
## Wald test            = 0.44  on 5 df,   p=1
## Score (logrank) test = 17.41  on 5 df,   p=0.004
```

The comparison of the survival time using `StT` can be performed using the log-rank test.

```
fit3 = survfit(surv0~StT,data=ytt)
ggsurvplot(fit3,risk.table=TRUE,pval=TRUE)
```

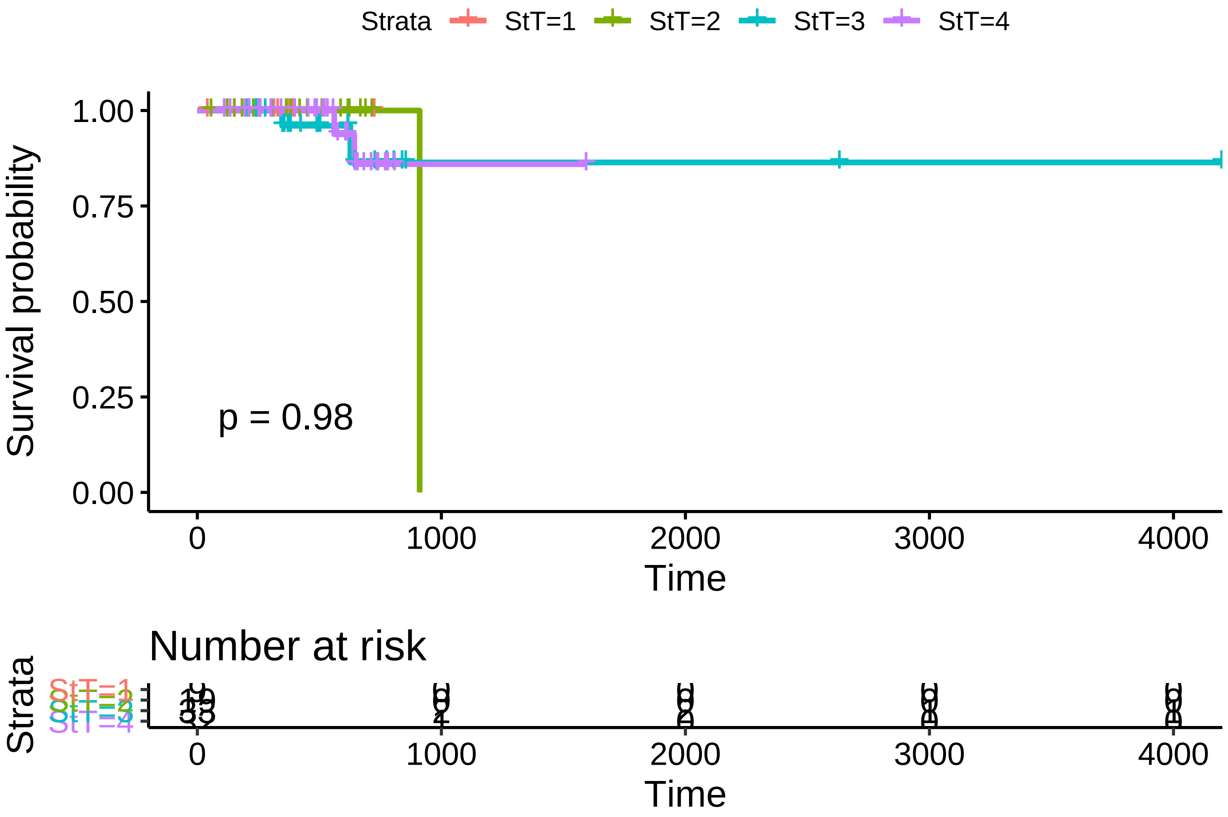

Global survival with `exprs.t`, `exprs.nt`, `StN`

Now, we are going to evaluate the joint behaviour of `exprs.t` and `exprs.nt` with `StStage`.

```
fit2 = coxph(surv0 ~ exprs.t + exprs.nt + StN,data=ytt,iter=1000)
```

```
## Warning in fitter(X, Y, strats, offset, init, control, weights = weights, :
## Loglik converged before variable 4 ; beta may be infinite.
```

A summary of the fit shows that there is no clear dependence of the Stage **given** the expression.

```r
summary(fit2)
```

```
## Call:
## coxph(formula = surv0 ~ exprs.t + exprs.nt + StN, data = ytt,
##     iter = 1000)
##
##   n= 92, number of events= 5
##    (5 observations deleted due to missingness)
##
##                coef  exp(coef)   se(coef)      z Pr(>|z|)
## exprs.t   1.853e-03  1.002e+00  1.691e-03  1.096    0.273
## exprs.nt  1.979e-02  1.020e+00  1.247e-02  1.587    0.112
## StN1     -5.312e-02  9.483e-01  1.310e+00 -0.041    0.968
## StN2     -2.161e+01  4.132e-10  1.424e+04 -0.002    0.999
##
##          exp(coef) exp(-coef) lower .95 upper .95
## exprs.t  1.002e+00  9.981e-01   0.99854     1.005
## exprs.nt 1.020e+00  9.804e-01   0.99537     1.045
## StN1     9.483e-01  1.055e+00   0.07279    12.354
## StN2     4.132e-10  2.420e+09   0.00000       Inf
##
## Concordance= 0.81  (se = 0.156 )
## Rsquare= 0.127   (max possible= 0.291 )
## Likelihood ratio test= 12.51  on 4 df,   p=0.01
## Wald test            = 0  on 4 df,   p=1
## Score (logrank) test = 22.28  on 4 df,   p=2e-04
```

The comparison of the survival time using `StN` can be performed using the log-rank test.

```r
fit3 = survfit(surv0~StN,data=ytt)
ggsurvplot(fit3,risk.table=TRUE,pval=TRUE)
```

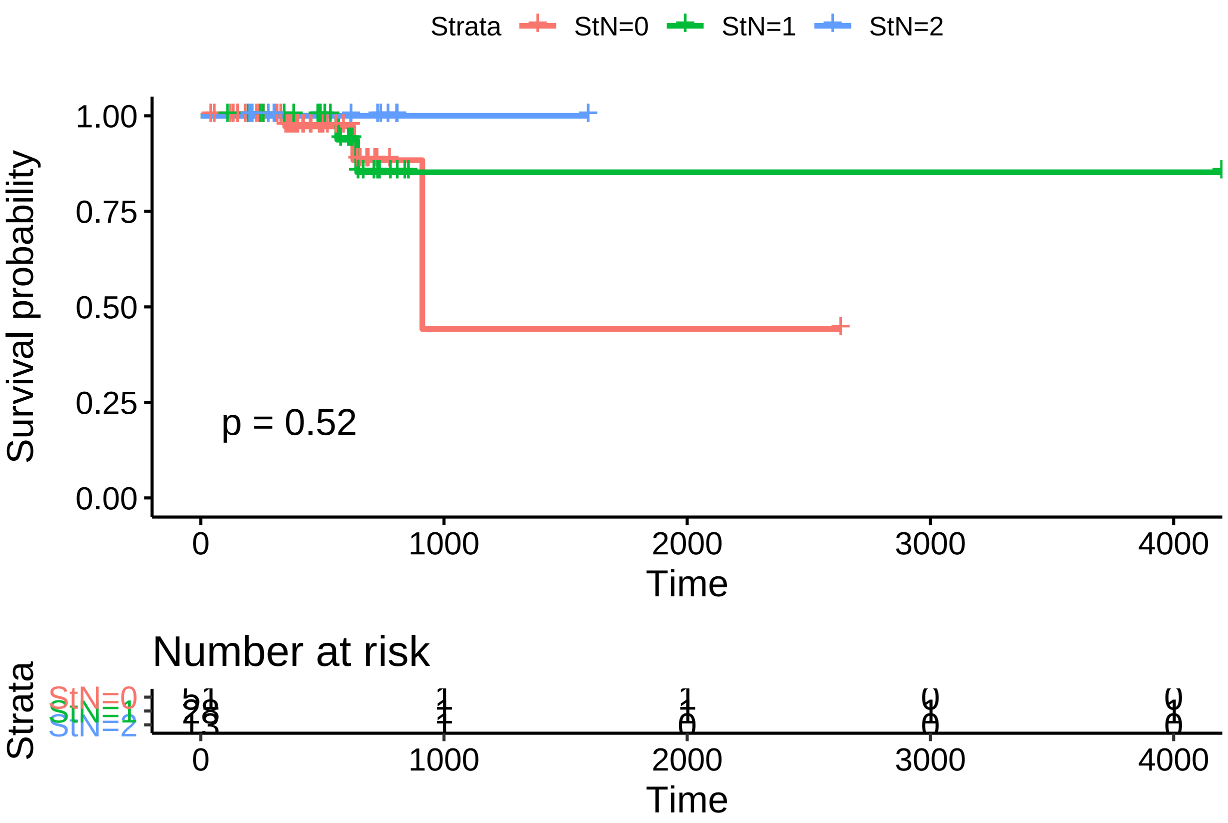

Global survival with `exprs.t`, `exprs.nt`

Now, we are going to evaluate the joint behaviour of `exprs.t` and `exprs.nt` with `StStage`.

```
fit2 = coxph(surv0 ~ exprs.t + exprs.nt,data=ytt,iter=1000)
```

A summary of the fit shows that there is no clear dependence of the Stage **given** the expression.

```
summary(fit2)
```

```
## Call:
## coxph(formula = surv0 ~ exprs.t + exprs.nt, data = ytt, iter = 1000)
##
##   n= 93, number of events= 5
##    (4 observations deleted due to missingness)
##
##              coef exp(coef) se(coef)     z Pr(>|z|)
## exprs.t  0.002226  1.002229 0.001293 1.722   0.0851 .
## exprs.nt 0.011243  1.011307 0.004067 2.765   0.0057 **
## ---
## Signif. codes:  0 '***' 0.001 '**' 0.01 '*' 0.05 '.' 0.1 ' ' 1
##
##          exp(coef) exp(-coef) lower .95 upper .95
## exprs.t      1.002     0.9978    0.9997     1.005
## exprs.nt     1.011     0.9888    1.0033     1.019
##
## Concordance= 0.78  (se = 0.157 )
## Rsquare= 0.078   (max possible= 0.289 )
```

```
## Likelihood ratio test= 7.52  on 2 df,   p=0.02
## Wald test            = 7.95  on 2 df,   p=0.02
## Score (logrank) test = 15.03  on 2 df,   p=5e-04
```

Looking for a threshold level for expression

It is clear that the survival time is function of the gene expression in normal and tumor tissue. High values of both variables are associated with a lesser survival time. Let us define the sum of both variables and look for a optimal threshold value.

```
exprs.total = yt$exprs.t + yt$exprs.nt
threshold.values = seq(200,700)
threshold.p.values = rep(NA,length(threshold.values))
for(i in 1:length(threshold.values)){
  fit2 = coxph(surv0 ~ I(exprs.total > threshold.values[i]))
  threshold.p.values[i] = summary(fit2)$logtest[3]
}

df = data.frame(threshold = threshold.values,p.value=threshold.p.values)
p = ggplot(df,aes(x=threshold.values,y=threshold.p.values)) + geom_line()
p + xlab("Threshold") + ylab("p-value")
```

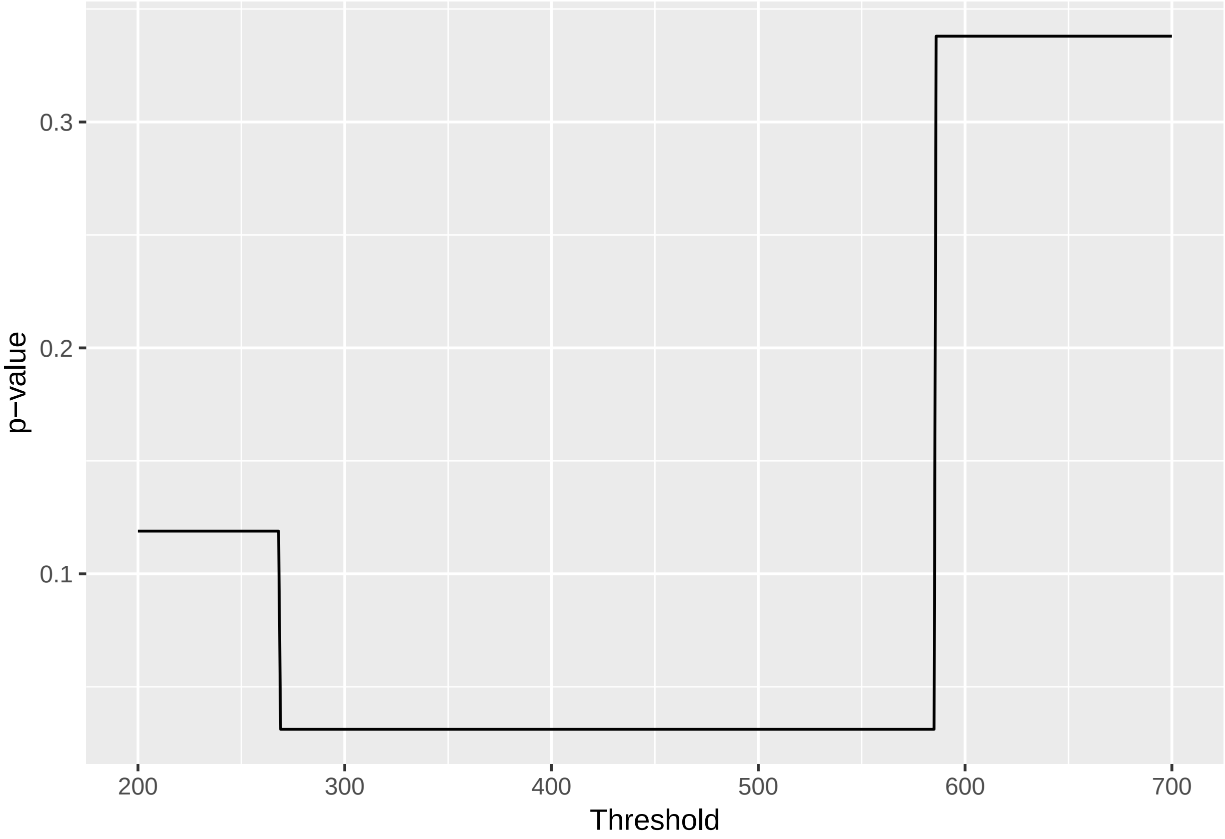

We take as threshold value 300.

```
fit = survfit(surv0~ I(exprs.total > 300) ,data=ytt)
ggsurvplot(fit,risk.table=TRUE,pval=TRUE)
```

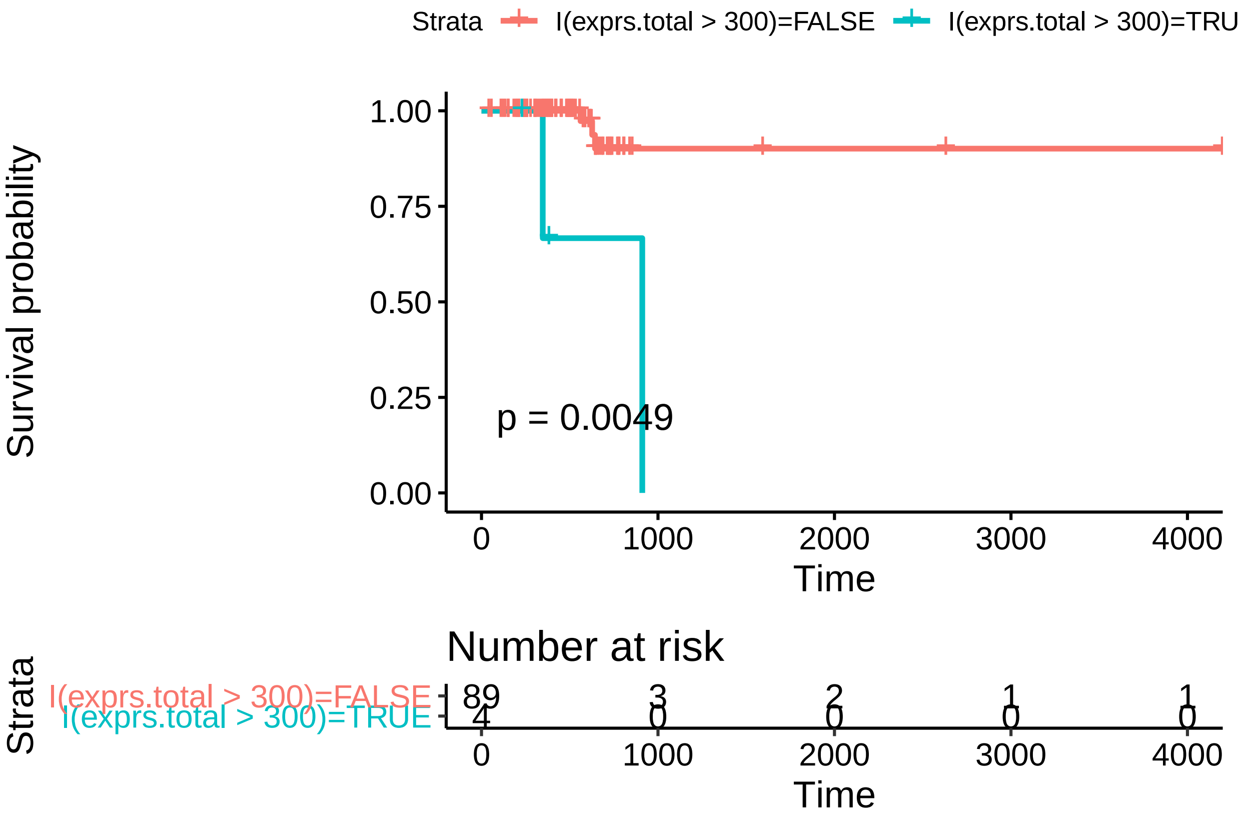

Fold change

Let us consider the dependence with respect to the fold-change.

```r
fit2 = coxph(surv0 ~fc,data=ytt)
summary(fit2)
```

```
## Call:
## coxph(formula = surv0 ~ fc, data = ytt)
##
##   n= 93, number of events= 5
##    (4 observations deleted due to missingness)
##
##         coef exp(coef)  se(coef)     z Pr(>|z|)
## fc 0.0006763 1.0006765 0.0007393 0.915     0.36
##
##    exp(coef) exp(-coef) lower .95 upper .95
## fc     1.001     0.9993    0.9992     1.002
##
## Concordance= 0.296  (se = 0.157 )
## Rsquare= 0.007   (max possible= 0.289 )
## Likelihood ratio test= 0.67  on 1 df,   p=0.4
## Wald test            = 0.84  on 1 df,   p=0.4
## Score (logrank) test = 0.89  on 1 df,   p=0.3
```

Note that there is no dependence. Is there dependence with a thresholded version of fc? The answer is no.

```r
summary(fc)
valth = seq(1,2500,length.out = 2500)
pval.fc = sapply(valth,function(th){
fit2 = coxph(surv0 ~I(fc>th),data=ytt)
aa = summary(fit2)
aa$coefficients[5]
}
)
save(pval.fc,file="pval.fc.rda")
```

```r
load("pval.fc.rda")
summary(pval.fc)
```

```
##    Min. 1st Qu.  Median    Mean 3rd Qu.    Max.
##  0.2894  0.2894  0.2894  0.5864  0.9992  0.9992
```

It seems that two cases with very large survival times could be influential observations. Let us remove them from the study.

```r
ytt1 = ytt[ytt$tt <= 700,]
surv1 = Surv(ytt1$tt,ytt1$LFState2)
```

First we evaluate the effect of both expressions without any transformation.

```r
fit = coxph(surv1 ~ exprs.t + exprs.nt ,data=ytt1,iter=1000)
summary(fit)
```

```
## Call:
## coxph(formula = surv1 ~ exprs.t + exprs.nt, data = ytt1, iter = 1000)
##
##   n= 74, number of events= 3
##    (4 observations deleted due to missingness)
##
##                coef exp(coef)  se(coef)      z Pr(>|z|)
## exprs.t  -0.006368  0.993653  0.019139 -0.333   0.7394
## exprs.nt  0.018431  1.018602  0.010539  1.749   0.0803 .
## ---
## Signif. codes:  0 '***' 0.001 '**' 0.01 '*' 0.05 '.' 0.1 ' ' 1
##
##          exp(coef) exp(-coef) lower .95 upper .95
## exprs.t     0.9937     1.0064    0.9571     1.032
## exprs.nt    1.0186     0.9817    0.9978     1.040
##
## Concordance= 0.792  (se = 0.209 )
## Rsquare= 0.121   (max possible= 0.214 )
## Likelihood ratio test= 9.57  on 2 df,   p=0.008
## Wald test            = 3.65  on 2 df,   p=0.2
## Score (logrank) test = 38.08  on 2 df,   p=5e-09
```

In silico data

Data

```
x = read.csv("../data/inSilico/ALL_data_TCGA_colonyrectum_2.csv",
             header=TRUE,sep=";")
```

A summary of the data.

```
summary(x)
```

```
##                                                File_name
##  00106523-5b1d-44ad-a9f1-7d84db08722c.htseq.counts.gz:  1
##  00589871-e54f-492f-988f-502670edd606.htseq.counts.gz:  1
##  00cc9b4d-a847-464e-979a-7751e1a87ae3.htseq.counts.gz:  1
##  00f768f9-9e6c-4e84-bdba-c19368f7e522.htseq.counts.gz:  1
##  0134d0cc-e66b-4fda-804b-c4434ec00bd2.htseq.counts.gz:  1
##  020aa019-a3a4-4055-92ee-be824a597501.htseq.counts.gz:  1
##  (Other)                                             :667
##         Case_ID         pair                       Sample_Type  tumor_stage
##  TCGA-A6-2671:  2   Min.   :  1.0   Primary Tumor      :622   I   :113
##  TCGA-A6-2675:  2   1st Qu.:136.0   Solid Tissue Normal: 51   II  :251
##  TCGA-A6-2678:  2   Median :296.0                             III :188
##  TCGA-A6-2679:  2   Mean   :300.6                             IV  : 97
##  TCGA-A6-2680:  2   3rd Qu.:457.0                             NA's: 24
##  TCGA-A6-2682:  2   Max.   :624.0
##  (Other)     :661
##  vital_status days_to_last_follow_up days_to_death    year_of_death
##       :  3    Min.   :   0.0         Min.   :   0.0   Min.   :1998
##  alive:527    1st Qu.: 365.0         1st Qu.: 214.0   1st Qu.:2003
##  dead :143    Median : 638.0         Median : 494.0   Median :2007
##               Mean   : 809.1         Mean   : 719.6   Mean   :2006
##               3rd Qu.:1080.5         3rd Qu.:1126.0   3rd Qu.:2009
##               Max.   :4502.0         Max.   :3042.0   Max.   :2012
##               NA's   :62             NA's   :530      NA's   :601
##     gender    year_of_birth  age_at_diagnosis
##        :  3   Min.   :1912   Min.   :11381
##  female:317   1st Qu.:1931   1st Qu.:21392
##  male  :353   Median :1940   Median :24936
##               Mean   :1941   Mean   :24462
##               3rd Qu.:1951   3rd Qu.:28164
##               Max.   :1980   Max.   :32872
##               NA's   :3      NA's   :5
```

Expression data.

```
load("../data/x1.rda")
row0 = which(data.frame(rowData(x1)) == "ENSG00000086289")
x2 = assay(x1)[row0,]
```

Survival time

```r
censored = is.na(x$days_to_death)
time = x$days_to_death
```

```r
table(is.na(x$days_to_last_follow_up))
```

```
##
## FALSE  TRUE
##   611    62
```

```r
time[censored] = x$days_to_last_follow_up[censored]
summary(time)
```

```
##    Min. 1st Qu.  Median    Mean 3rd Qu.    Max.    NA's
##       0     376     669     836    1096    4502       4
```

```r
x3 = data.frame(exprs=x2,time,censored,
                x[,c("pair","Sample_Type","tumor_stage","gender",
                     "age_at_diagnosis")])
```

Fitting a survival object.

```r
surv0 = Surv(x3$time,x3$censored)
fit = survfit(surv0~1,data=x3)
```

The Kaplan-Meier curve can be obtained with

```r
ggsurvplot(fit)
```

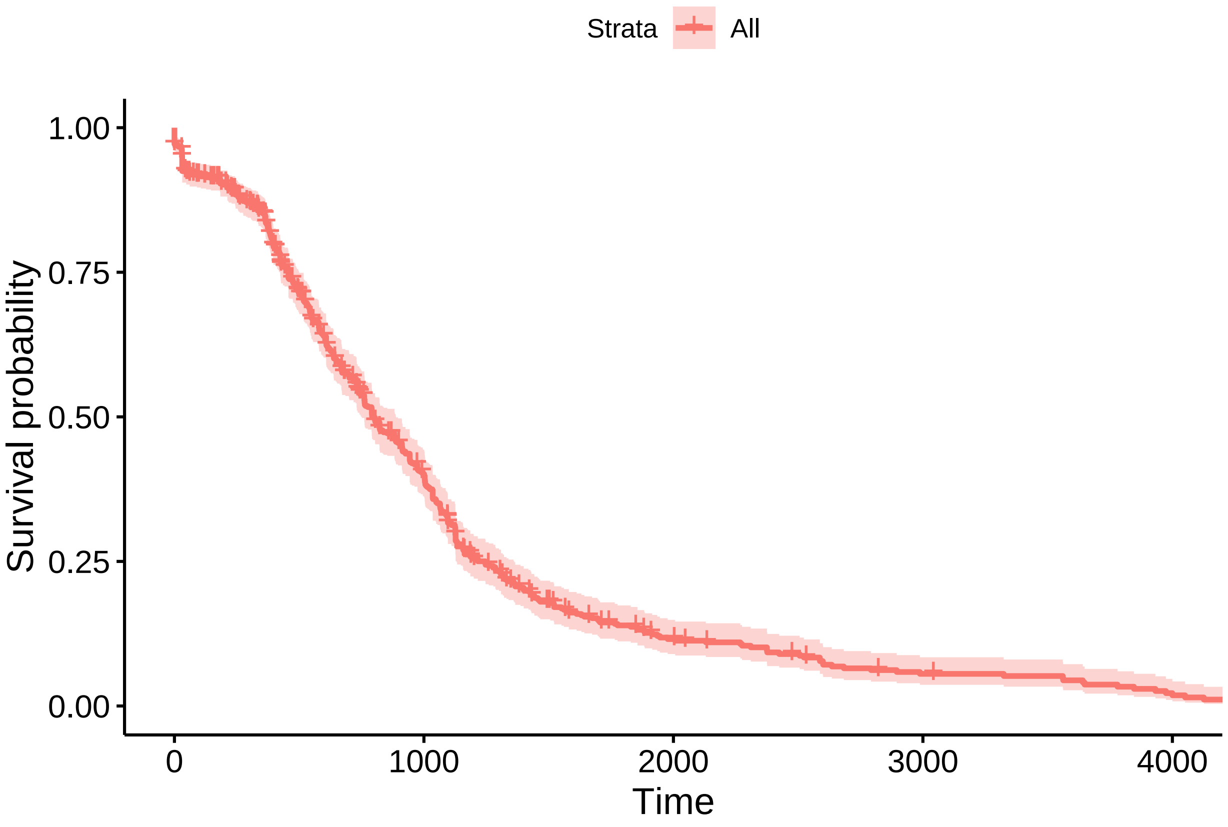

Is there a dependence of the tumor stage?

First a summary of the absolute frequencies.

```r
table(x3$tumor_stage)
```

```
##
##   I  II III  IV
## 113 251 188  97
```

```r
fit1 = survfit(surv0~tumor_stage,data=x3)
ggsurvplot(fit1,data=x,risk.table=TRUE,pval=TRUE)
```

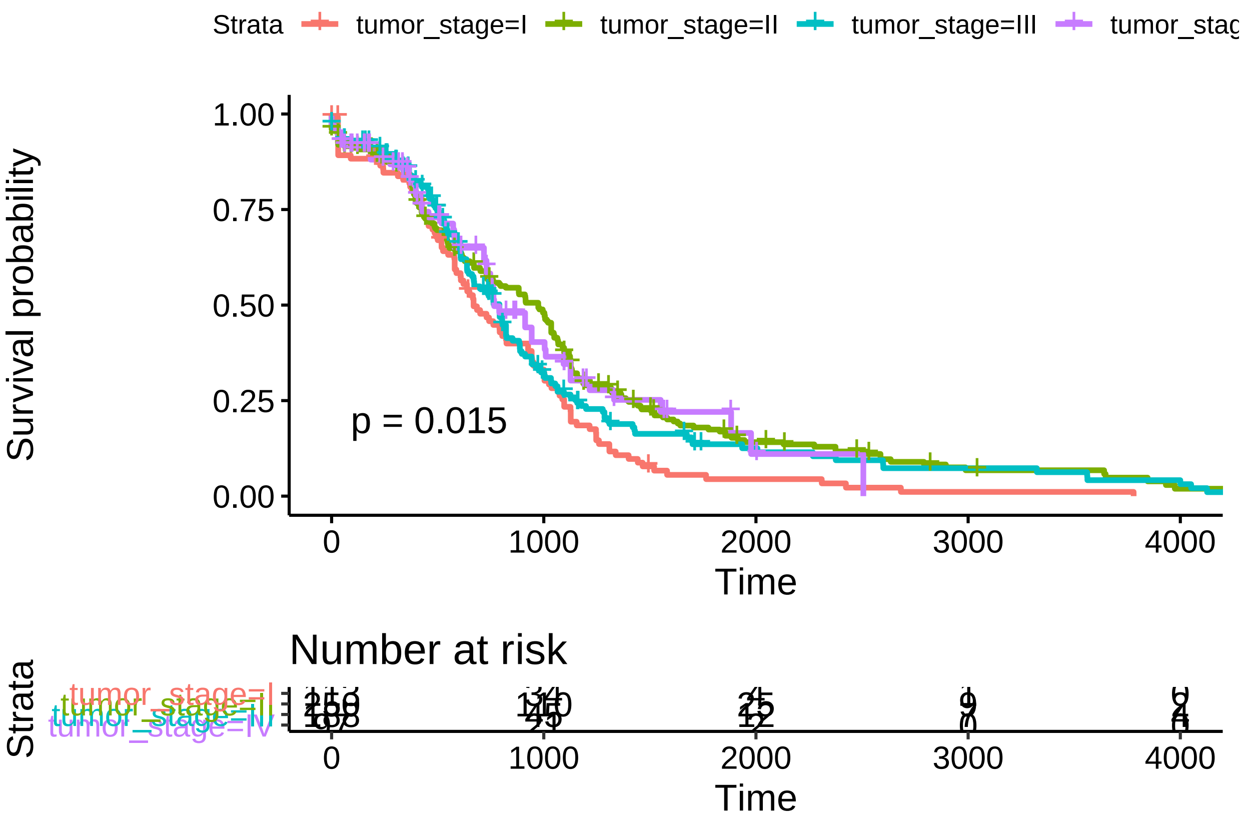


```r
names(x3)
```

```
## [1] "exprs"           "time"            "censored"
## [4] "pair"            "Sample_Type"     "tumor_stage"
## [7] "gender"          "age_at_diagnosis"
```

```r
levels(x3$Sample_Type)
```

```
## [1] "Primary Tumor"       "Solid Tissue Normal"
```

```r
fit2 = coxph(surv0~exprs+ Sample_Type + tumor_stage,data=x3)
summary(fit2)
```

```
## Call:
## coxph(formula = surv0 ~ exprs + Sample_Type + tumor_stage, data = x3)
##
##   n= 648, number of events= 514
##    (25 observations deleted due to missingness)
##
##                                  coef  exp(coef)   se(coef)      z
## exprs                      -8.495e-05  9.999e-01  4.212e-05 -2.017
```

```
## Sample_TypeSolid Tissue Normal -1.517e-01  8.593e-01  1.754e-01 -0.865
## tumor_stageII                   -3.702e-01  6.906e-01  1.210e-01 -3.061
## tumor_stageIII                  -2.191e-01  8.032e-01  1.305e-01 -1.679
## tumor_stageIV                   -3.004e-01  7.405e-01  1.708e-01 -1.759
##                                 Pr(>|z|)
## exprs                            0.04368 *
## Sample_TypeSolid Tissue Normal   0.38702
## tumor_stageII                    0.00221 **
## tumor_stageIII                   0.09307 .
## tumor_stageIV                    0.07864 .
## ---
## Signif. codes:  0 '***' 0.001 '**' 0.01 '*' 0.05 '.' 0.1 ' ' 1
##
##                                 exp(coef) exp(-coef) lower .95 upper .95
## exprs                              0.9999      1.000    0.9998    1.0000
## Sample_TypeSolid Tissue Normal     0.8593      1.164    0.6093    1.2117
## tumor_stageII                      0.6906      1.448    0.5448    0.8753
## tumor_stageIII                     0.8032      1.245    0.6220    1.0373
## tumor_stageIV                      0.7405      1.350    0.5298    1.0350
##
## Concordance= 0.535  (se = 0.015 )
## Rsquare= 0.022   (max possible= 1 )
## Likelihood ratio test= 14.59  on 5 df,   p=0.01
## Wald test            = 14.94  on 5 df,   p=0.01
## Score (logrank) test = 15.04  on 5 df,   p=0.01
```

```
fit = coxph(surv0~exprs + tumor_stage,data=x3)
summary(fit)
```

```
## Call:
## coxph(formula = surv0 ~ exprs + tumor_stage, data = x3)
##
##   n= 648, number of events= 514
##    (25 observations deleted due to missingness)
##
##                      coef  exp(coef)   se(coef)      z Pr(>|z|)
## exprs          -8.070e-05  9.999e-01  4.184e-05 -1.929  0.05378 .
## tumor_stageII  -3.774e-01  6.856e-01  1.207e-01 -3.127  0.00177 **
## tumor_stageIII -2.173e-01  8.047e-01  1.305e-01 -1.665  0.09589 .
## tumor_stageIV  -3.086e-01  7.344e-01  1.706e-01 -1.809  0.07039 .
## ---
## Signif. codes:  0 '***' 0.001 '**' 0.01 '*' 0.05 '.' 0.1 ' ' 1
##
##                exp(coef) exp(-coef) lower .95 upper .95
## exprs             0.9999      1.000    0.9998    1.0000
## tumor_stageII     0.6856      1.459    0.5412    0.8686
## tumor_stageIII    0.8047      1.243    0.6231    1.0392
## tumor_stageIV     0.7344      1.362    0.5257    1.0260
##
## Concordance= 0.532  (se = 0.015 )
## Rsquare= 0.021   (max possible= 1 )
## Likelihood ratio test= 13.81  on 4 df,   p=0.008
## Wald test            = 14.17  on 4 df,   p=0.007
## Score (logrank) test = 14.28  on 4 df,   p=0.006
```

```r
fit = coxph(surv0~ tumor_stage,data=x3)
summary(fit)
```

```
## Call:
## coxph(formula = surv0 ~ tumor_stage, data = x3)
##
##   n= 648, number of events= 514
##    (25 observations deleted due to missingness)
##
##                     coef exp(coef) se(coef)      z Pr(>|z|)
## tumor_stageII  -0.3830    0.6818   0.1207 -3.173  0.00151 **
## tumor_stageIII -0.2508    0.7782   0.1296 -1.936  0.05289 .
## tumor_stageIV  -0.3351    0.7152   0.1700 -1.971  0.04870 *
## ---
## Signif. codes:  0 '***' 0.001 '**' 0.01 '*' 0.05 '.' 0.1 ' ' 1
##
##                exp(coef) exp(-coef) lower .95 upper .95
## tumor_stageII     0.6818      1.467    0.5382    0.8638
## tumor_stageIII    0.7782      1.285    0.6037    1.0031
## tumor_stageIV     0.7152      1.398    0.5125    0.9981
##
## Concordance= 0.526  (se = 0.014 )
## Rsquare= 0.015   (max possible= 1 )
## Likelihood ratio test= 9.91  on 3 df,   p=0.02
## Wald test            = 10.41  on 3 df,   p=0.02
## Score (logrank) test = 10.5  on 3 df,   p=0.01
```

```r
fit = coxph(surv0~ exprs + Sample_Type,data=x3)
summary(fit)
```

```
## Call:
## coxph(formula = surv0 ~ exprs + Sample_Type, data = x3)
##
##   n= 669, number of events= 526
##    (4 observations deleted due to missingness)
##
##                                       coef  exp(coef)   se(coef)      z
## exprs                           -9.374e-05  9.999e-01  4.151e-05 -2.258
## Sample_TypeSolid Tissue Normal -2.124e-01  8.087e-01  1.743e-01 -1.219
##                                Pr(>|z|)
## exprs                            0.0239 *
## Sample_TypeSolid Tissue Normal   0.2230
## ---
## Signif. codes:  0 '***' 0.001 '**' 0.01 '*' 0.05 '.' 0.1 ' ' 1
##
##                                exp(coef) exp(-coef) lower .95 upper .95
## exprs                             0.9999      1.000    0.9998     1.000
## Sample_TypeSolid Tissue Normal    0.8087      1.237    0.5747     1.138
##
## Concordance= 0.525  (se = 0.015 )
## Rsquare= 0.009   (max possible= 1 )
## Likelihood ratio test= 6.3  on 2 df,   p=0.04
## Wald test            = 6.02  on 2 df,   p=0.05
## Score (logrank) test = 6.02  on 2 df,   p=0.05
```

Expression

It is interesting to compare the expression between the groups defined by `Sample_Type`.

```
t.test(exprs ~ Sample_Type,data=x3)
```

```
##
##  Welch Two Sample t-test
##
## data:  exprs by Sample_Type
## t = 8.5174, df = 195.69, p-value = 4.306e-15
## alternative hypothesis: true difference in means is not equal to 0
## 95 percent confidence interval:
##  413.6707 662.9588
## sample estimates:
##       mean in group Primary Tumor mean in group Solid Tissue Normal
##                         1338.1383                          799.8235
```

The means in both groups are significant different at any usual level of significance. We are going to compare the expression at different levels of `tumor_stage`.

```
summary(aov(exprs ~ tumor_stage,data=x3))
```

```
##              Df    Sum Sq Mean Sq F value Pr(>F)
## tumor_stage   3  11636206 3878735   3.253 0.0214 *
## Residuals   645 768986449 1192227
## ---
## Signif. codes:  0 '***' 0.001 '**' 0.01 '*' 0.05 '.' 0.1 ' ' 1
## 24 observations deleted due to missingness
```

The pairwise comparisons can be performed using the Benjamini-Hochberg correction.

```
pairwise.t.test(x3$exprs,x3$tumor_stage,p.adjust.method = "BH")
```

```
##
##  Pairwise comparisons using t tests with pooled SD
##
## data:  x3$exprs and x3$tumor_stage
##
##     I     II    III
## II  0.717 -     -
## III 0.049 0.049 -
## IV  0.049 0.049 0.717
##
## P value adjustment method: BH
```

If we use a false discovery rate of .05 then it can be considered that there exist a significant difference between for the following pairs (I,III) (I,IV), (II,III), (II,IV). The following groups will have no significant differences within the group: $\{I, II\}$, $\{III, IV\}$.

oriGene data

Reading the data

Now, we read the data.

```r
xo = read.xlsx("../data/Incliva/OriGeneEPDR1.xlsx", 1)
levels(xo$stage) = c("0","I","II","II","III","III","III","III","IV")
summary(xo)
```

```
##      sample          tipo     stage       gender     EPDR1.total
##  Min.   : 1.00   Normal: 5   0  : 5   Female:27   Min.   :0.00697
##  1st Qu.:12.75   Tumor :43   I  :10   Male  :21   1st Qu.:0.12948
##  Median :24.50               II :13               Median :0.31109
##  Mean   :24.50               III:14               Mean   :0.53832
##  3rd Qu.:36.25               IV : 6               3rd Qu.:0.59421
##  Max.   :48.00                                    Max.   :4.18448
##     EPDR1.2
##  Min.   :0.00000
##  1st Qu.:0.00000
##  Median :0.02496
##  Mean   :0.04897
##  3rd Qu.:0.06219
##  Max.   :0.43712
```

Let us see the frequencies of the variable stage for this data set.

```r
table(xo[,"stage"])
```

```
##
##   0   I  II III  IV
##   5  10  13  14   6
```

Total expression of EPDR1

First we will do some violin plots describing the data.

```r
png("figures/EPDR1_total_tipo.png")
ggplot(xo, aes(x = tipo , y = EPDR1.total)) +  geom_violin() +
  ylab("Total expression")
dev.off()
png("figures/EPDR1_total_stage.png")
ggplot(xo, aes(x = stage , y = EPDR1.total)) +  geom_violin() +
   ylab("Total expression")
dev.off()
png("figures/EPDR1_total_gender.png")
ggplot(xo, aes(x =  gender, y = EPDR1.total)) +  geom_violin() +
   ylab("Total expression")
dev.off()
```

We compare the expressions using the usual anova approach and multiple comparisons.

```r
summary(aov(EPDR1.total ~ stage,data=xo))
```

```
##             Df Sum Sq Mean Sq F value Pr(>F)
## stage        4  4.759  1.1897   2.736 0.0409 *
## Residuals   43 18.697  0.4348
## ---
## Signif. codes:  0 '***' 0.001 '**' 0.01 '*' 0.05 '.' 0.1 ' ' 1
```

The pairwise comparisons can be performed using the Benjamini-Hochberg correction.

```r
pairwise.t.test(xo[,"EPDR1.total"],xo[,"stage"],p.adjust.method = "BH")
```

```
##
##  Pairwise comparisons using t tests with pooled SD
##
## data:  xo[, "EPDR1.total"] and xo[, "stage"]
##
##     0     I     II    III
## I   0.593 -     -     -
## II  0.069 0.081 -     -
## III 0.327 0.593 0.189 -
## IV  0.493 0.777 0.228 0.842
##
## P value adjustment method: BH
```

No pairwise difference is considered significant. Let us use a permutation approach for the same problem. The permutation test (instead the asymptotic approach assuming normality assumption) is

```r
independence_test(EPDR1.total ~ stage,data = xo)
```

```
##
##  Asymptotic General Independence Test
##
## data:  EPDR1.total by stage (0, I, II, III, IV)
## maxT = 2.7651, p-value = 0.02738
## alternative hypothesis: two.sided
```

We can see the p-value is lesser than the observed p-value using the anova approach. Now the pairwise permutation tests.

```r
pairwisePermutationTest(EPDR1.total ~ stage,data = xo,method="BH")
```

```
##      Comparison    Stat p.value p.adjust
## 1      0 - I = 0  -1.708  0.0877   0.1754
## 2     0 - II = 0  -1.777 0.07563   0.1754
## 3    0 - III = 0   -2.51 0.01208   0.1208
## 4     0 - IV = 0  -2.064 0.03899   0.1754
## 5     I - II = 0   -1.77 0.07668   0.1754
## 6    I - III = 0  -1.338   0.181   0.2586
## 7     I - IV = 0 -0.7441  0.4568   0.5076
## 8   II - III = 0   1.525  0.1272   0.2120
## 9    II - IV = 0   1.161  0.2455   0.3069
## 10 III - IV = 0  0.3806  0.7035   0.7035
```

The comparisons between the group 0 are close to be significant but they are not using the Benjamini-Hochberg correction.

Isoform EPDR1.2

First we will do some violin plots describing the data.

```r
png("figures/EPDR1_2_tipo.png")
ggplot(xo, aes(x = tipo , y = EPDR1.2)) +  geom_violin() +
  ylab("Isoform 2")
dev.off()
png("figures/EPDR1_2_stage.png")
```

```
ggplot(xo, aes(x = stage , y = EPDR1.2)) +  geom_violin() +
   ylab("Isoform 2")
dev.off()
png("figures/EPDR1_2_gender.png")
ggplot(xo, aes(x =  gender, y = EPDR1.2)) +  geom_violin() +
   ylab("Isoform 2")
dev.off()
```

First the anova.

```
summary(aov(EPDR1.2 ~ stage,data=xo))
```

```
##             Df  Sum Sq  Mean Sq F value Pr(>F)
## stage        4 0.05062 0.012654   2.244   0.08 .
## Residuals   43 0.24247 0.005639
## ---
## Signif. codes:  0 '***' 0.001 '**' 0.01 '*' 0.05 '.' 0.1 ' ' 1
```

The pairwise comparisons can be performed using the Benjamini-Hochberg correction.

```
pairwise.t.test(xo[,"EPDR1.2"],xo[,"stage"],p.adjust.method = "BH")
```

```
##
##  Pairwise comparisons using t tests with pooled SD
##
## data:  xo[, "EPDR1.2"] and xo[, "stage"]
##
##     0    I    II   III
## I   0.60 -    -    -
## II  0.16 0.20 -    -
## III 0.60 0.85 0.16 -
## IV  0.79 0.79 0.16 0.80
##
## P value adjustment method: BH
```

Now the permutation test.

```
independence_test(EPDR1.2 ~ stage,data = xo)
```

```
##
##  Asymptotic General Independence Test
##
## data:  EPDR1.2 by stage (0, I, II, III, IV)
## maxT = 2.6511, p-value = 0.0382
## alternative hypothesis: two.sided
```

The pairwise comparisons are

```
pairwisePermutationTest(EPDR1.2 ~ stage,data = xo,method="BH")
```

```
##      Comparison   Stat p.value p.adjust
## 1     0 - I = 0 -1.485  0.1375   0.2750
## 2    0 - II = 0 -1.606  0.1082   0.2705
## 3   0 - III = 0 -2.304 0.02122   0.2122
## 4    0 - IV = 0 -1.774 0.07614   0.2705
## 5    I - II = 0  -1.27  0.2042   0.2917
## 6   I - III = 0 0.3224  0.7472   0.7472
## 7    I - IV = 0  0.743  0.4575   0.5083
```

```
## 8  II - III = 0  1.704 0.08837   0.2705
## 9   II - IV = 0  1.372  0.1702   0.2837
## 10 III - IV = 0 0.9341  0.3503   0.4379
```

Similar comments to the total EPDR1. It is better the permutation approach. The results are not significant but some of them are close.

Is there difference in the distribution for EPDR1.2?

```
ks.results = NULL
for( i in 1:(nlevels(xo$stage)-1)){
  for(j in (i+1):nlevels(xo$stage)){
    cat("i, j ",i,j,"\n")
    a = ks.test(xo$EPDR1.2[xo$stage == levels(xo$stage)[i]],
        xo$EPDR1.2[xo$stage == levels(xo$stage)[j]],alternative="greater",data = xo)
    ks.results = rbind(ks.results,c(levels(xo$stage)[i], levels(xo$stage)[j],a$p.value))
  }
}
```

```
## i, j  1 2
## i, j  1 3
## i, j  1 4
## i, j  1 5
## i, j  2 3
## i, j  2 4
## i, j  2 5
## i, j  3 4
## i, j  3 5
## i, j  4 5
```

```
ks.results
```

```
##       [,1]  [,2]  [,3]
##  [1,] "0"   "I"   "0.0140284668601351"
##  [2,] "0"   "II"  "0.00567922686437719"
##  [3,] "0"   "III" "0.0105791290066811"
##  [4,] "0"   "IV"  "0.0885451733162887"
##  [5,] "I"   "II"  "0.228186506547294"
##  [6,] "I"   "III" "0.668725944419122"
##  [7,] "I"   "IV"  "0.967216100482006"
##  [8,] "II"  "III" "0.676293996696751"
##  [9,] "II"  "IV"  "0.978639005309246"
## [10,] "III" "IV"  "1"
```

```
p.adjust(ks.results[,3],method = "BH")
```

```
##  [1] 0.04676156 0.04676156 0.04676156 0.22136293 0.45637301 0.96613428
##  [7] 1.00000000 0.96613428 1.00000000 1.00000000
```

```
ks.results = NULL
for( i in 1:(nlevels(xo$stage)-1)){
  for(j in (i+1):nlevels(xo$stage)){
    cat("i, j ",i,j,"\n")
    a = ks.test(xo$EPDR1.2[xo$stage == levels(xo$stage)[i]],
        xo$EPDR1.2[xo$stage == levels(xo$stage)[j]],alternative="less",data = xo)
    ks.results = rbind(ks.results,c(levels(xo$stage)[i], levels(xo$stage)[j],a$p.value))
```

```
  }
}
```

```
## i, j  1 2
## i, j  1 3
## i, j  1 4
## i, j  1 5
## i, j  2 3
## i, j  2 4
## i, j  2 5
## i, j  3 4
## i, j  3 5
## i, j  4 5
```

```
ks.results
```

```
##       [,1]  [,2]  [,3]
##  [1,] "0"   "I"   "1"
##  [2,] "0"   "II"  "1"
##  [3,] "0"   "III" "1"
##  [4,] "0"   "IV"  "1"
##  [5,] "I"   "II"  "0.958094019344632"
##  [6,] "I"   "III" "0.889881770988024"
##  [7,] "I"   "IV"  "0.586646219510032"
##  [8,] "II"  "III" "0.128517647211735"
##  [9,] "II"  "IV"  "0.173949917049035"
## [10,] "III" "IV"  "0.295511472445308"
```

It is clear that, when the Kolmogorov-Smirnov test is applied we reject the equality of the cumulative distribution function when we compare the group “0” with the groups “I”, “II” and “III”. There exist no other significant test.
